# Supplementary material for: Genome-wide analyses identify a role for SLC17A4 and AADAT in thyroid hormone regulation
Source: Nat Commun. 2018 Oct 26;9:4455. doi: 10.1038/s41467-018-06356-1 (PMC6203810; doi:10.1038/s41467-018-06356-1)
Supplement: Supplementary file 1 — Supplementary Information [file 41467_2018_6356_MOESM1_ESM.pdf]

Genome-wide analyses identify a role for SLC17A4 and AADAT in thyroid hormone regulation

Teumer *et al.*

SUPPLEMENTARY INFORMATION

Table of Contents

Supplementary Note 1: Detailed results of mouse candidate analyses

Supplementary Note 2: Pathway analyses results

Supplementary Note 3: Lookup for pleiotropy of novel loci

Supplementary Note 4: Extended acknowledgements

Supplementary Figure 1: Schematic of the project

Supplementary Figure 2: Regional association plots for TSH

Supplementary Figure 3: Regional association plots for FT4

Supplementary Figure 4: Forest plots for TSH

Supplementary Figure 5: Forest plots for FT4

Supplementary Figure 6: Manhattan plots for GWAS meta-analyses in the sex-stratified samples

Supplementary Figure 7: Genes related to abnormal thyroid morphology in genetically manipulated mice are enriched for SNPs significantly associated with TSH concentrations in humans

Supplementary Figure 8: Manhattan plot for GWAS meta-analyses for hypo- vs. hyperthyroidism

Supplementary Figure 9: Thyroid hormone transport by MCT8 in comparison to hSLC17A4

Supplementary Figure 10: QQ plots for all GWAS meta-analyses in the overall samples

Supplementary Figure 11: QQ plots for all GWAS meta-analyses in the sex-stratified samples

Supplementary Figure 12: Results of pathway analyses

Supplementary Table 1: Genome-wide significant index SNPs for TSH after final stage

Supplementary Table 2: Genome-wide significant index SNPs for FT4 after final stage

Supplementary Table 3: Distribution of sex and age group for hypo- and hyperthyroidism GWAS cases and controls

Supplementary Table 4: Genome-wide significant index SNPs for hypo- and hyperthyroidism

Supplementary Table 5: Lookup of TSH associated SNPs in Graves' disease and TPOab positivity

Supplementary Table 6: Association of SLC17A4 and AADAT hits with T3 and T3/T4 ratio

Supplementary Table 7: GRS association with hypo- and hyperthyroidism

Supplementary Table 8: Association results of TSH-based genetic risk score

Supplementary Table 9: Association results of FT4-based genetic risk score

Supplementary Table 10: Replication results of GWAS SNPs

Supplementary References

Supplementary Note 1: Detailed results of mouse candidate analyses

In this candidate gene approach we selected genes that are linked to abnormal thyroid physiology (i.e., hormone levels, n=26) or morphology (n=51) in mice to assess whether their human homologs contained SNPs significantly associated with FT4 and TSH in our GWAS. For FT4, variants in 6 and 3 genes and for TSH in 4 and 5 genes (**Supplementary Data 2**) from the abnormal murine thyroid physiology and morphology list, respectively, showed significant association (**Supplementary Figure 7A**). Four of them (*TPO*, *CGA*, *TRHR*, and *FOXE1*) did not reach genome-wide significance in the full TSH or FT4 GWAS. Notably, *CGA* and *TRHR* were never been detected by previous GWAS of thyroid function or thyroid diseases. Both *CGA* and *TRHR* represent excellent biological candidates; *CGA* codes for the alpha chain of TSH, and *TRHR* for the thyrotropin (TSH) releasing hormone receptor expressed in the pituitary. To validate these four additional associations, we sought replication of the variants in 9,011 (TSH) and 4,532 (FT4) independent samples. As shown in **Supplementary Figure 7B and C,** both *CGA* and *TPO* achieved genome-wide significance for TSH ($p<5x10^{-8}$), whereas *TRHR* approached this threshold for FT4 ($p=5.7x10^{-8}$, **Supplementary Data 2**).

Supplementary Note 2: Pathway analyses results

Top canonical pathways identified for the TSH hits included G-protein signaling and ephrin receptor signaling, both important for neurogenesis and neuronal sustainability during development, as well as cardiac beta-adrenergic signaling (**Supplemental Figure 12A**), which overlap with the top pathways revealed for hyperthyroidism. The top canonical pathways identified for FT4 did not overlap with those for TSH but partially with those for hypothyroidism and included thyroid hormone metabolism and biosynthesis, and thyroid hormone receptor/retinoid-X receptor activation. The canonical pathway related to tRNA splicing overlapped with both hypo- and hyperthyroidism, whereas other pathways like

cAMP-mediated signaling and T-cell receptor signaling were specific for hyperthyroidism and hypothyroidism, respectively. For TSH, the top networks incorporated genes involved in embryonic development, cell cycle control and cell faith decision. For FT4, the top networks incorporated genes encoding proteins involved in endocrine system development and function as well as molecular transport and small molecule biochemistry.

The DEPICT results assessing tissue-specificity of the GWAS results did not reveal any associations that passed the significance level after multiple testing correction (all false discovery rates were ≥0.2). However, the only thyroid tissue related association with a nominal significant p-value was found for the TSH GWAS results with thyroid gland, which was also the strongest tissue enrichment for this trait (**Supplemental Figure 12B**). Additionally, nominally significant tissue enrichment was revealed in the pancreas in which also AADAT and SLC17A4 co-localized.

Supplementary Note 3: Lookup for pleiotropy of novel loci

Pleiotropic effects of the novel loci associated with TSH included body height (*ADCY9, MIR365A*), waist circumference (*ADCY9*), type II diabetes (*IGF2BP2*), as well as age at menarche (*MIR365A*) and ovarian cancer (*NSF*), and intracranial volume and Parkinson's disease (*NSF*). Loci associated with FT4 were also associated with body height, body mass index, proinsulin and high-density lipoprotein (*FNBP4*), with blood metabolite levels (*ACMSD*: N1-methyl-3-pyridone-4-carboxamide, *SLCO1B1*: isoleucine), and serum urate and hemoglobin levels (*SLC17A4*). The loci associated with hypo- or hyperthyroidism were almost exclusively additionally associated with thyroid related traits including TSH levels, thyroid volume and thyroid cancer. The detailed results of genome-wide significant pleiotropic effects are provided in **Supplementary Data 5,** and a description of the new loci with respect to gene function is provided in **Supplementary Data 6**.

Supplementary Note 4: Extended acknowledgements

The Genotype-Tissue Expression (GTEx) Project was supported by the Common Fund of the Office of the Director of the National Institutes of Health, and by NCI, NHGRI, NHLBI, NIDA, NIMH, and NINDS.

ARIC

The Atherosclerosis Risk in Communities Study is carried out as a collaborative study supported by National Heart, Lung, and Blood Institute contracts (HHSN268201100005C, HHSN268201100006C, HHSN268201100007C, HHSN268201100008C, HHSN268201100009C, HHSN268201100010C, HHSN268201100011C, and HHSN268201100012C), R01HL087641, R01HL59367 and R01HL086694; National Human Genome Research Institute contract U01HG004402; and National Institutes of Health contract HHSN268200625226C. The authors thank the staff and participants of the ARIC study for their important contributions. Infrastructure was partly supported by Grant Number UL1RR025005, a component of the National Institutes of Health and NIH Roadmap for Medical Research. Reagents for the thyroid assays in the ARIC study were donated by Roche Diagnostics. Dr. Selvin was supported by NIH/NIDDK grants K24DK106414 and R01DK089174. Dr. Köttgen was supported by DFG grants KO 3598/3-1 (Heisenberg professorship), CRC 1140 and CRC 992. The work of Dr. Li was supported by DFG grant KO 3598/4-1.

ASKLEPIOS

The Asklepios study was supported by grants from the Research Foundation Flanders (FWO G.0427.03 and G.0838.10N). A full list of Asklepios investigators can be found with Rietzschel et al.[1]

BHS

The 1994/1995 Busselton Health Survey was supported and funded by the Health Promotion

Foundation of Western Australia.

BioBank Japan Project

This research was supported by the Tailor-Made Medical Treatment program (the BioBank Japan Project) of the Ministry of Education, Culture, Sports, Science, and Technology (MEXT) and the Japan Agency for Medical Research and Development (AMED), and a grant from the Ministry of Health, Welfare, and Labour for the Scientific Research Project for Longevity (NH: 1999-2003). Yukinori Okada was supported by the Japan Society for the Promotion of Science (JSPS) KAKENHI (15H05670, 15H05907, 15H05911, 15K14429, 16H03269, and 16K15738), AMED (16km0405206h0001, 16gm6010001h0001, and 17ek0410041h0001), Takeda Science Foundation, the Uehara Memorial Foundation, the Naito Foundation, Daiichi Sankyo Foundation of Life Science, and Senri Life Science Foundation.

CHRIS

The CHRIS (Cooperative Health Research In South Tyrol) study is a collaborative effort between the Eurac Research Institute for Biomedicine and the Healthcare System of the Autonomous Province of Bolzano. The study investigators wish to thank all study participants, all general practitioners of the middle and upper Vinschgau/Val Venosta, the personnel of the hospital of Schlanders/Silandro, and the personnel of the department of laboratory medicine of the hospital of Meran/Merano for their support. Full acknowledgments are reported here: https://translational-medicine.biomedcentral.com/articles/10.1186/s12967-015-0704-9#Declarations. The CHRIS study was funded by the Department of Innovation, Research and University of the Autonomous Province of Bolzano-South Tyrol.

CHS

This research was supported by contracts HHSN268201200036C, HHSN268200800007C,

N01HC55222, N01HC85079, N01HC85080, N01HC85081, N01HC85082, N01HC85083, N01HC85086, and grants U01HL080295 U01HL130114 and R01AG027058 from the National Heart, Lung, and Blood Institute (NHLBI), with additional contribution from the National Institute of Neurological Disorders and Stroke (NINDS). Additional support was provided by R01AG023629 from the National Institute on Aging (NIA). Support for the Cardiovascular Health Study Whole Genome Study was provided by NHLBI grant HL087652. Additional support for infrastructure was provided by HL105756 and additional genotyping among the African-American cohort was supported in part by HL085251. DNA handling and genotyping at Cedars-Sinai Medical Center was supported in part by National Center for Research Resources grant UL1RR033176, now at the National Center for Advancing Translational Technologies CTSI grant UL1TR001881; in addition to the National Institute of Diabetes and Digestive and Kidney Diseases grant DK063491 to the Southern California Diabetes Endocrinology Research Center. A full list of principal CHS investigators and institutions can be found at CHS-NHLBI.org. The content is solely the responsibility of the authors and does not necessarily represent the official views of the National Institutes of Health.

GARP

We thank all study participants of the GARP study. This research in GARP was supported by the Leiden University Medical Centre, the Dutch Arthritis Foundation (DAF-10-1-402; DAF-15-4-401) and Pfizer Inc., Groton, CT, USA. Additional funding contributions came from the Netherlands Organization of Scientific Research (MW 904-61-095, 911-03-016, 917-66-344 and 911-03-012; ZonMW VICI scheme 91816631/528), the “Centre of Medical System Biology” and the “Netherlands Consortium of Healthy Aging” in the framework of the Netherlands Genomics Initiative (NGI).

GCKD

We thank the patients, study personnel and the large number of nephrologists who all contribute to the GCKD study (http://www.gckd.org). The GCKD study is funded by grants from the German Ministry of Education and Research (BMBF, grant number 01ER0804), the KfH Foundation for Preventive Medicine and corporate partners. Genotyping was supported by Bayer Pharma AG. The work of Dr. Schultheiss was funded by the CRC 1140 of the German Research Foundation (DFG) and by the Else Kroener Fresenius Foundation (EKFK NAKSYS). The work of J. Jing was funded by the Chinese Scholarship Council.

HBCS

We thank all study participants as well as everybody involved in the Helsinki Birth Cohort Study. Helsinki Birth Cohort Study has been supported by grants from the Academy of Finland, the Finnish Diabetes Research Society, Folkhälsan Research Foundation, Novo Nordisk Foundation, Finska Läkaresällskapet, Juho Vainio Foundation, Signe and Ane Gyllenberg Foundation, University of Helsinki, Ministry of Education, Ahokas Foundation, Emil Aaltonen Foundation.

Health2006

The Health2006 study was financially supported by grants from the Velux Foundation; the Danish Medical Research Council, Danish Agency for Science, Technology and Innovation; the Aase and Ejner Danielsens Foundation; ALK-Abello´ A/S (Hørsholm, Denmark), Timber Merchant Vilhelm Bangs Foundation, MEKOS Laboratories (Denmark) and Research Centre for Prevention and Health, the Capital Region of Denmark. The Novo Nordisk Foundation Center for Basic Metabolic Research is an independent Research Center at the University of Copenhagen partially funded by an unrestricted donation from the Novo Nordisk Foundation (www.metabol.ku.dk).

Inter99

We acknowledge all members of the Inter99 team and the steering committee of the Inter99 study: Torben Jørgensen, DMSc (PI), Knut Borch-Johnsen, DMSc (Co-PI), Hans Ibsen, DMSc, Troels Thomsen, Ph.D, Charlotta Pisinger, PhD and Charlotte Glümer, PhD. The study was initiated and analyzed by the investigators in this project. The work was supported economically by: The Danish Medical Research Council, The Danish Centre for Evaluation and Health Technology Assessment, Novo Nordisk, Copenhagen County, The Danish Heart Foundation, The Danish Pharmaceutical Association, Augustinus foundation, Ib Henriksen foundation and Becket foundation. The Novo Nordisk Foundation Center for Basic Metabolic Research is an independent Research Center at the University of Copenhagen partially funded by an unrestricted donation from the Novo Nordisk Foundation (www.metabol.ku.dk).

KORA

The KORA research platform was initiated and financed by the Helmholtz Center Munich, by the German Research Center for Environmental Health, which is funded by the German Federal Ministry of Education and Research, and by the State of Bavaria. The work of KORA is supported by the German Federal Ministry of Education and Research (BMBF) in the context of the German National Genome Research Network (NGFN-2 and NGFN-plus). Our research was supported within the Munich Center of Health Sciences (MC Health) as part of LMUinnovativ. Thyroid examinations were funded by Sanofi-Aventis within the framework of the Papillon Initiative.

LBC1921 and LBC1936

We thank the cohort participants and team members who contributed to these studies. Phenotype collection in the Lothian Birth Cohort 1921 was supported by the UK's Biotechnology and Biological Sciences Research Council (BBSRC), The Royal Society, and The

Chief Scientist Office of the Scottish Government. Phenotype collection in the Lothian Birth Cohort 1936 was supported by Age UK (The Disconnected Mind project). Genotyping of the cohorts was funded by the BBSRC (BB/F019394/1). The work was undertaken by The University of Edinburgh Centre for Cognitive Ageing and Cognitive Epidemiology, part of the cross council Lifelong Health and Wellbeing Initiative (MR/K026992/1). Funding from the BBSRC and Medical Research Council (MRC) is gratefully acknowledged.

LifeLines

The Lifelines Cohort Study, and generation and management of GWAS genotype data for the Lifelines Cohort Study is supported by the Netherlands Organization of Scientific Research NWO (grant 175.010.2007.006), the Economic Structure Enhancing Fund (FES) of the Dutch government, the Ministry of Economic Affairs, the Ministry of Education, Culture and Science, the Ministry for Health, Welfare and Sports, the Northern Netherlands Collaboration of Provinces (SNN), the Province of Groningen, University Medical Center Groningen, the University of Groningen, Dutch Kidney Foundation and Dutch Diabetes Research Foundation. We thank Behrooz Alizadeh, Annemieke Boesjes, Marcel Bruinenberg, Noortje Festen, Pim van der Harst, Ilja Nolte, Lude Franke, Mitra Valimohammadi for their help in creating the GWAS database, and Rob Bieringa, Joost Keers, René Oostergo, Rosalie Visser, Judith Vonk for their work related to data collection and validation. The authors are grateful to the study participants, the staff from the Lifelines Cohort Study and the contributing research centers delivering data to Lifelines and the participating general practitioners and pharmacists. Statistical analyses were carried out on the Genetic Cluster Computer (http://www.geneticcluster.org) hosted by SURFsara and financially supported by the Netherlands Scientific Organization (NWO 480-05-003 PI: Posthuma) along with a supplement from the Dutch Brain Foundation and the VU University Amsterdam.

Lifelines Cohort Study group authors: Behrooz Z Alizadeh, H Marike Boezen, Lude Franke, Pim van der Harst, Gerjan Navis, Marianne Rots, Harold Snieder, Ronald P Stolk, Morris Swertz, Bruce HR Wolffenbuttel, Cisca Wijmenga.

LLS

The Leiden Longevity Study has received funding from the European Union's Seventh Framework Programme (FP7/2007-2011) under grant agreement number 259679. This study was financially supported by the Innovation-Oriented Research Program on Genomics (SenterNovem IGE05007), the Centre for Medical Systems Biology and the Netherlands Consortium for Healthy Ageing (grant 050-060-810), all in the framework of the Netherlands Genomics Initiative, Netherlands Organization for Scientific Research (NWO), by Unilever Colworth and by BBMRI-NL, a Research Infrastructure financed by the Dutch government (NWO 184.021.007).

MICROS

The MICROS (Microisolates in South Tyrol) study investigators wish to thank all study participants, the general practitioners and the personnel of the Hospital of Silandro (Department of Laboratory Medicine) for their participation and collaboration in the research project. The study was supported by the Ministry of Health and Department of Educational Assistance, University and Research of the Autonomous Province of Bolzano, the South Tyrolean Sparkasse Foundation, and the European Union framework program 6 EUROSPAN project (contract no. LSHG-CT-2006-018947).

NBS

The Nijmegen Biomedical Study is a population-based survey conducted at the Department for Health Evidence and the Department of Laboratory Medicine of the Radboud university medical center. Principal investigators of the Nijmegen Biomedical Study are L.A.L.M.

Kiemeney, A.L.M. Verbeek, D.W. Swinkels en B. Franke.

PROSPER

The PROSPER study was supported by an investigator initiated grant obtained from Bristol-Myers Squibb. Prof. Dr. J. W. Jukema is an Established Clinical Investigator of the Netherlands Heart Foundation (grant 2001 D 032). Support for genotyping was provided by the seventh framework program of the European commission (grant 223004) and by the Netherlands Genomics Initiative (Netherlands Consortium for Healthy Aging grant 050-060-810).

Rotterdam Study

The Rotterdam Study[2] is funded by Erasmus Medical Center and Erasmus University, Rotterdam, Netherlands Organization for the Health Research and Development (ZonMw), the Research Institute for Diseases in the Elderly (RIDE), the Ministry of Education, Culture and Science, the Ministry for Health, Welfare and Sports, the European Commission (DG XII), and the Municipality of Rotterdam. The authors are grateful to the study participants, the staff from the Rotterdam Study and the participating general practitioners and pharmacists.

The Rotterdam Study has been approved by the Medical Ethics Committee of the Erasmus MC (registration number MEC 02.1015) and by the Dutch Ministry of Health, Welfare and Sport (Population Screening Act WBO, license number 1071272-159521-PG). The Rotterdam Study has been entered in the Netherlands National Trial Register (NTR; http://www.trialregister.nl) and in the WHO International Clinical Trials Registry Platform (ICTRP; http://www.who.int/ictrp/network/primary/en/) under shared catalogue number NTR6831. All participants provided written informed consent to participate in the study and to have their information obtained from treating physicians.

The generation and management of GWAS genotype data for the Rotterdam Study (RS I, RS

II, RS III) was executed by the Human Genotyping Facility of the Genetic Laboratory of the Department of Internal Medicine, Erasmus MC, Rotterdam, The Netherlands. The GWAS datasets are supported by the Netherlands Organisation of Scientific Research NWO Investments (nr. 175.010.2005.011, 911-03-012), the Genetic Laboratory of the Department of Internal Medicine, Erasmus MC, the Research Institute for Diseases in the Elderly (014-93-015; RIDE2), the Netherlands Genomics Initiative (NGI)/Netherlands Organisation for Scientific Research (NWO) Netherlands Consortium for Healthy Aging (NCHA), project nr. 050-060-810. We thank Pascal Arp, Mila Jhamai, Marijn Verkerk, Lizbeth Herrera and Marjolein Peters, MSc, and Carolina Medina-Gomez, MSc, for their help in creating the GWAS database, and Karol Estrada, PhD, Yurii Aulchenko, PhD, and Carolina Medina-Gomez, MSc, for the creation and analysis of imputed data.

This study makes use of sequence reference data generated by the UK10K Consortium, derived from samples from the ALSPAC and TwinsUK datasets. A full list of the investigators who contributed to the generation of the data is available from www.UK10K.org. Funding for UK10K was provided by the Wellcome Trust under award WT091310. We thank Jie Huang at the Wellcome Trust's Sanger Institute, at Hinxton, U.K. for the creation of imputed data, with the support of Marijn Verkerk, Carolina Medina-Gomez, MSc, and Anis Abuseiris and their input for the analysis setup.

This study makes use of an extended dataset of RS II and RS III samples based on Illumina Omni 2.5 and 5.0 GWAS genotype data, imputed to 1KG using the two-phase imputation method[3]. This dataset was funded by the Genetic Laboratory of the Department of Internal Medicine, the department of Forensic Molecular Biology, and the department of Dermatology, Erasmus MC, Rotterdam, The Netherlands. This work was supported by a grant from the Netherlands Organisation for Health Research and Development (project number

113303005) (to WEV) and from the Sherman Foundation (to WEV). We thank Linda Broer, PhD, for the creation of imputed data, with the support of Marijn Verkerk and Carolina Medina-Gomez, MSc, for the analysis setup.

We would like to thank Karol Estrada PhD, Fernando Rivadeneira PhD, and Anis Abuseiris (Erasmus MC Rotterdam, The Netherlands), for their help in creating GRIMP, and we thank BigGRID for access to their grid computing resources[4].

SardiNIA

The SardiNIA project is supported in part by the intramural program of the National Institute on Aging through contracts N01-AG-1-2109 and HHSN271201100005C to the Consiglio Nazionale delle Ricerche of Italy. The SardiNIA authors would like to thank the CRS4 HPC group in Italy for the computational infrastructure supporting this project, and all volunteers who generously participated in the study.

SHIP/SHIP-Trend

SHIP is part of the Community Medicine Research net of the University of Greifswald, Germany, which is funded by the Federal Ministry of Education and Research (grants no. 01ZZ9603, 01ZZ0103, and 01ZZ0403), the Ministry of Cultural Affairs as well as the Social Ministry of the Federal State of Mecklenburg-West Pomerania, and the network 'Greifswald Approach to Individualized Medicine (GANI_MED)' funded by the Federal Ministry of Education and Research (grant 03IS2061A). Genome-wide data have been supported by the Federal Ministry of Education and Research (grant no. 03ZIK012) and a joint grant from Siemens Healthineers, Erlangen, Germany and the Federal State of Mecklenburg-West Pomerania. The University of Greifswald is a member of the Caché Campus program of the InterSystems GmbH.

SIBLOS

The SIBLing Osteoporosis Study (SIBLOS) was designed to investigate the genetic and environmental determinants of bone metabolism and hormonal exposure in men. This study is supported by grants from the Fund for Scientific Research – Flanders (FWO-Vlaanderen grants #G.0662.07 and #G.0867.11). The authors are indebted to K Toye, K Mertens, E Vandersypt, and M Becque for their excellent technical assistance, and all the volunteers for participation in the study.

TwinsUK

TwinsUK is funded by the Wellcome Trust, Medical Research Council, European Union, the National Institute for Health Research (NIHR)-funded BioResource, Clinical Research Facility and Biomedical Research Centre based at Guy's and St Thomas' NHS Foundation Trust in partnership with King's College London. This research was also supported by the Australian National Health and Medical Research Council (Project Grant 1087407), the Sir Charles Gairdner Hospital RAC (SGW) and the iVEC/Pawsey Supercomputing Centre (Project Grants: Pawsey0162, Director2025 (SGW)).

Val Borbera

Val Borbera is an isolated valley in North Western Italy. We thank all the participants in the project, the San Raffaele Hospital MDs who contributed to clinical data collection, Prof. Clara Camaschella who coordinated the data collection, Corrado Masciullo and Massimiliano Cocca for the database informatics. The research was supported by funds from Compagnia di San Paolo, Torino, Italy; Fondazione Cariplo, Italy; Telethon Italy; Ministry of Health, Ricerca Finalizzata 2008 and 2011-2012 and Public Health Genomics Project 2010.

Supplementary Figure 1: Schematic of the project

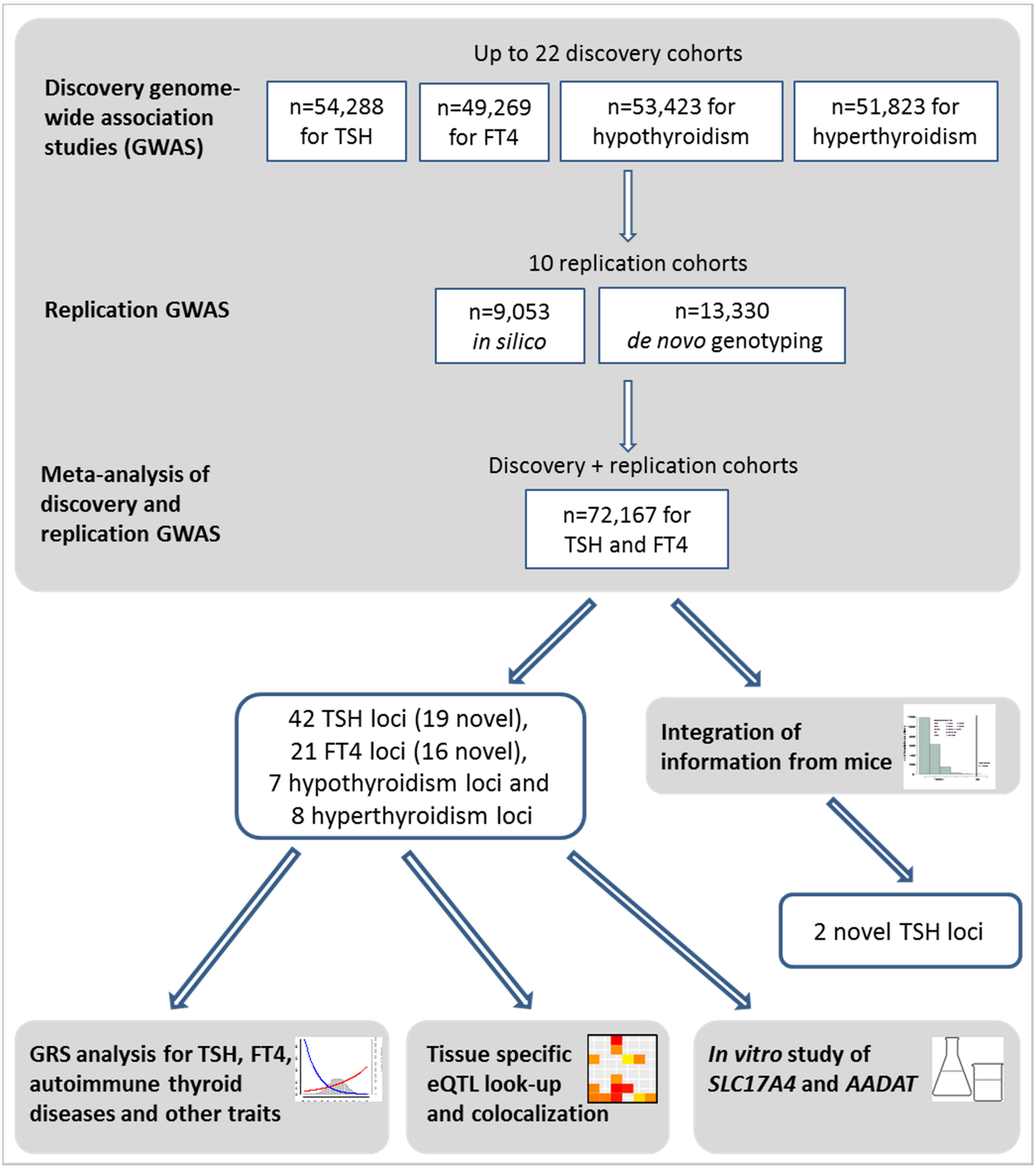

Overview of the workflow of the project showing its major sub-analyses.

Supplementary Figure 2: Regional association plots for TSH

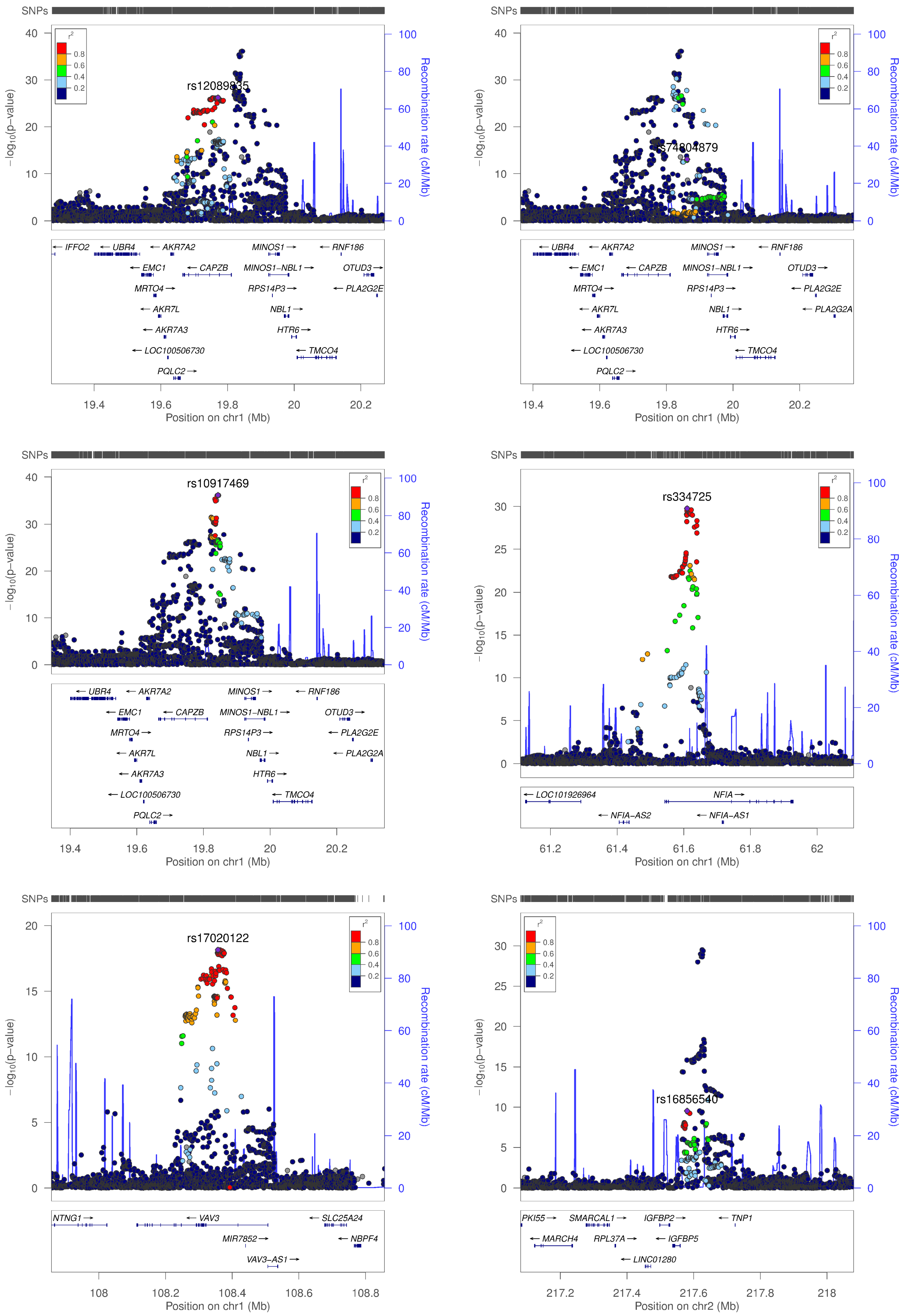
SNPs
rs12089835
r^2
0.8
0.6
0.4
0.2
−log10(p−value)
Recombination rate (cM/Mb)
IFFO2
UBR4
AKR7A2
EMC1
CAPZB
MRTO4
AKR7L
AKR7A3
LOC100506730
PQLC2
MINOS1
MINOS1−NBL1
RPS14P3
NBL1
HTR6
TMCO4
RNF186
OTUD3
PLA2G2E
Position on chr1 (Mb)
rs74804879
PLA2G2A
rs10917469
rs334725
LOC101926964
NFIA
NFIA−AS2
NFIA−AS1
rs17020122
NTNG1
VAV3
SLC25A24
MIR7852
NBPF4
VAV3−AS1
rs16856540
PKI55
SMARCAL1
IGFBP2
TNP1
MARCH4
RPL37A
IGFBP5
LINC01280
Position on chr2 (Mb)

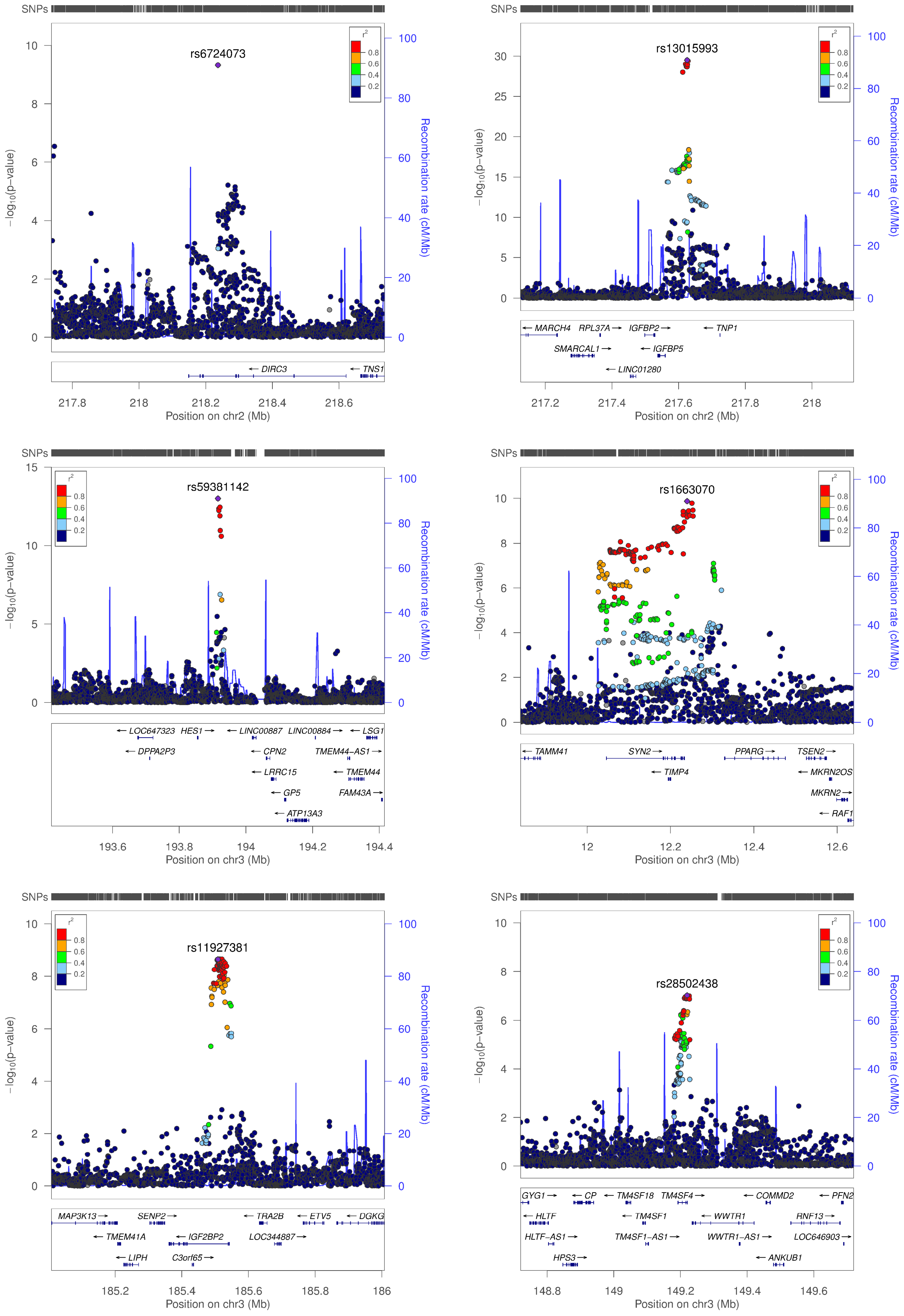

rs6724073
Position on chr2 (Mb)
DIRC3
TNS1
rs13015993
Position on chr2 (Mb)
MARCH4
RPL37A
IGFBP2
TNP1
SMARCAL1
IGFBP5
LINC01280
rs59381142
Position on chr3 (Mb)
LOC647323
HES1
LINC00887
LINC00884
LSG1
DPPA2P3
CPN2
TMEM44-AS1
LRRC15
TMEM44
GP5
FAM43A
ATP13A3
rs1663070
Position on chr3 (Mb)
TAMM41
SYN2
PPARG
TSEN2
TIMP4
MKRN2OS
MKRN2
RAF1
rs11927381
Position on chr3 (Mb)
MAP3K13
SENP2
TRA2B
ETV5
DGKG
TMEM41A
IGF2BP2
LOC344887
LIPH
C3orf65
rs28502438
Position on chr3 (Mb)
GYG1
CP
TM4SF18
TM4SF4
COMMD2
PFN2
HLTF
TM4SF1
WWTR1
RNF13
HLTF-AS1
TM4SF1-AS1
WWTR1-AS1
LOC646903
HPS3
ANKUB1
SNPs
−log10(p−value)
Recombination rate (cM/Mb)
r2

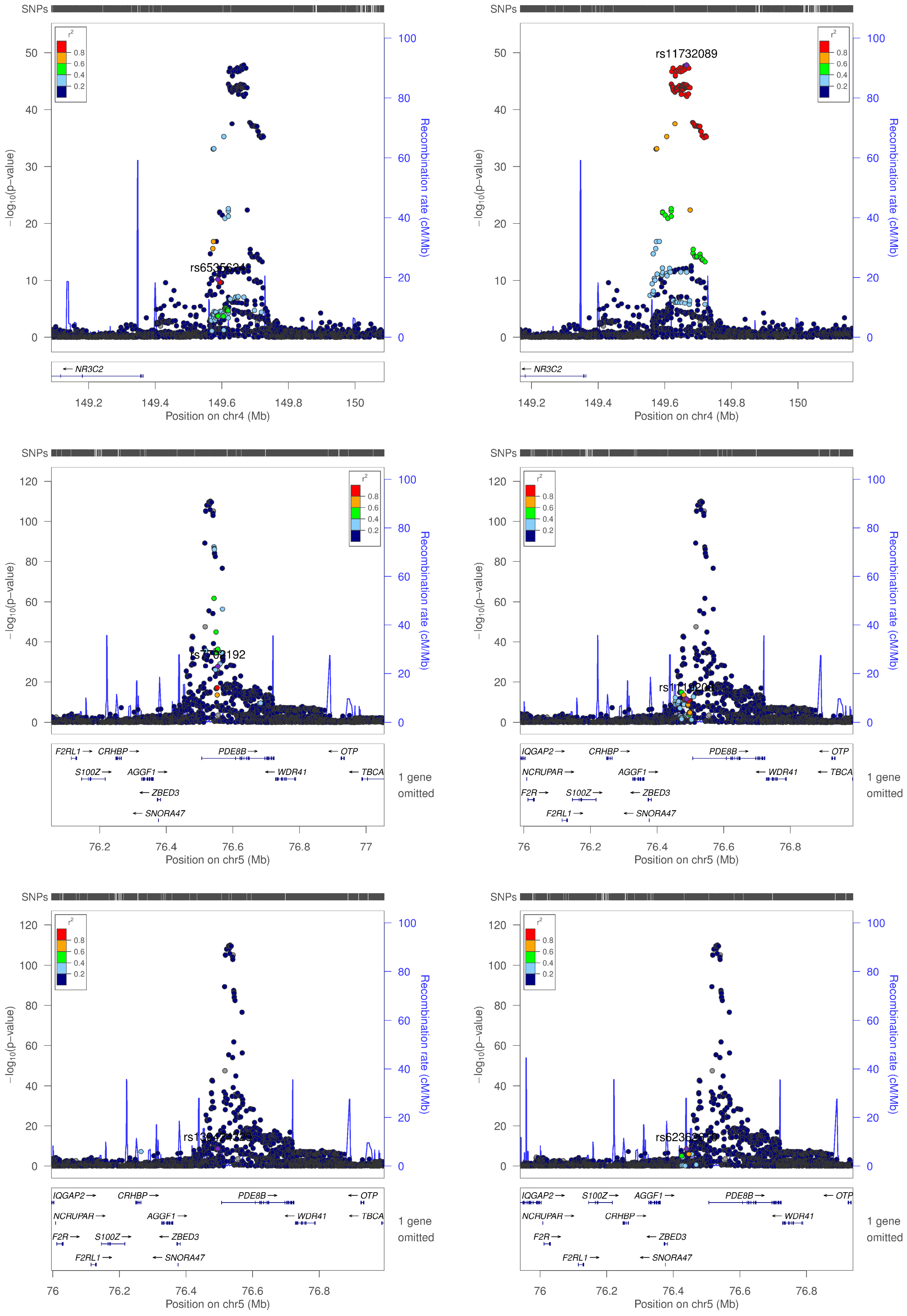
SNPs
−log10(p−value)
Recombination rate (cM/Mb)
NR3C2
Position on chr4 (Mb)
rs11732089
F2RL1
CRHBP
PDE8B
OTP
S100Z
AGGF1
WDR41
TBCA
ZBED3
SNORA47
IQGAP2
NCRUPAR
F2R
1 gene omitted
Position on chr5 (Mb)

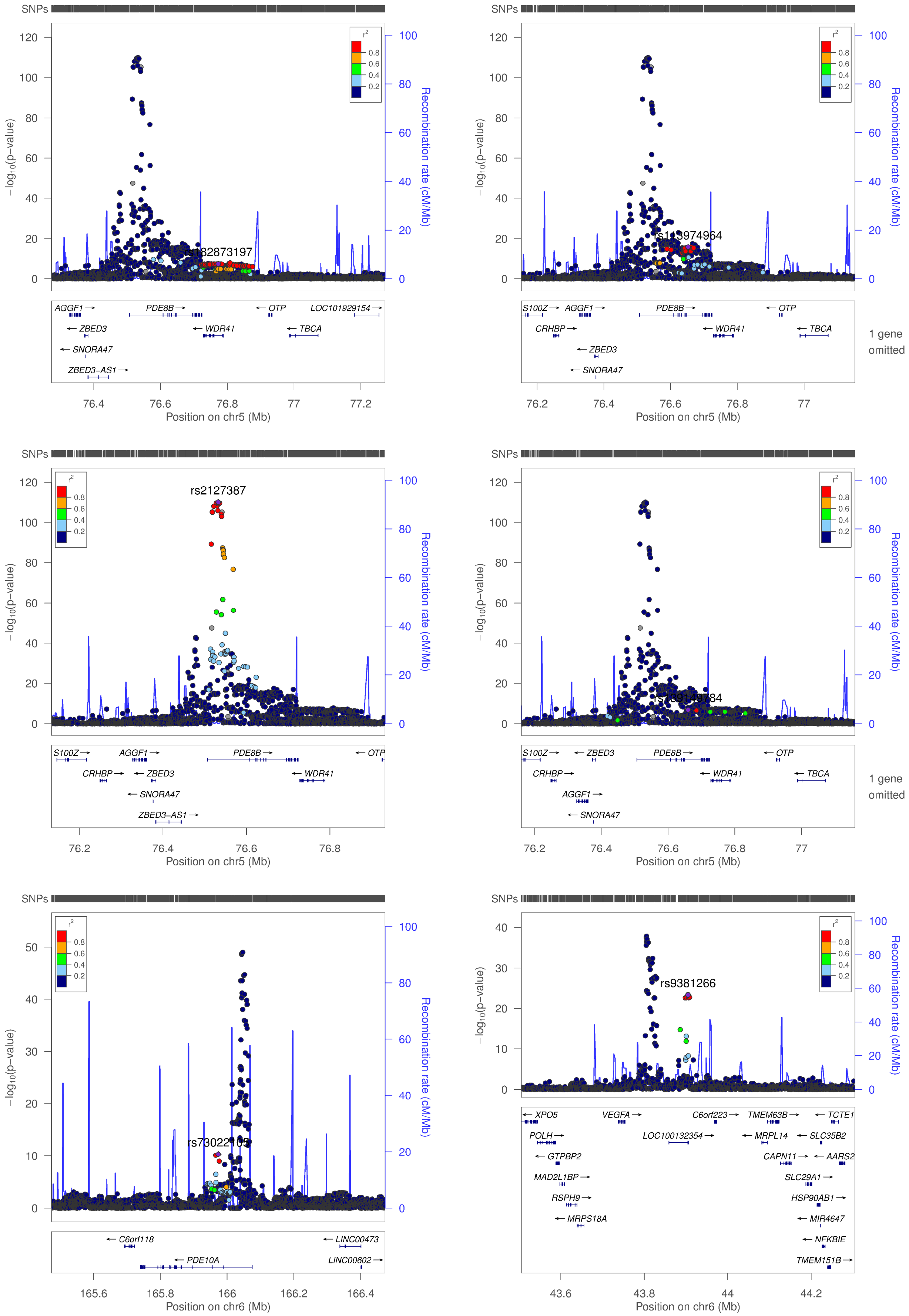

SNPs
−log10(p−value)
Recombination rate (cM/Mb)
r2
0.8
0.6
0.4
0.2
AGGF1
PDE8B
OTP
LOC101929154
ZBED3
WDR41
TBCA
SNORA47
ZBED3−AS1
S100Z
CRHBP
1 gene omitted
Position on chr5 (Mb)
rs2127387
C6orf118
LINC00473
PDE10A
LINC00602
Position on chr6 (Mb)
rs9381266
XPO5
VEGFA
C6orf223
TMEM63B
TCTE1
POLH
LOC100132354
MRPL14
SLC35B2
GTPBP2
CAPN11
AARS2
MAD2L1BP
SLC29A1
RSPH9
HSP90AB1
MRPS18A
MIR4647
NFKBIE
TMEM151B

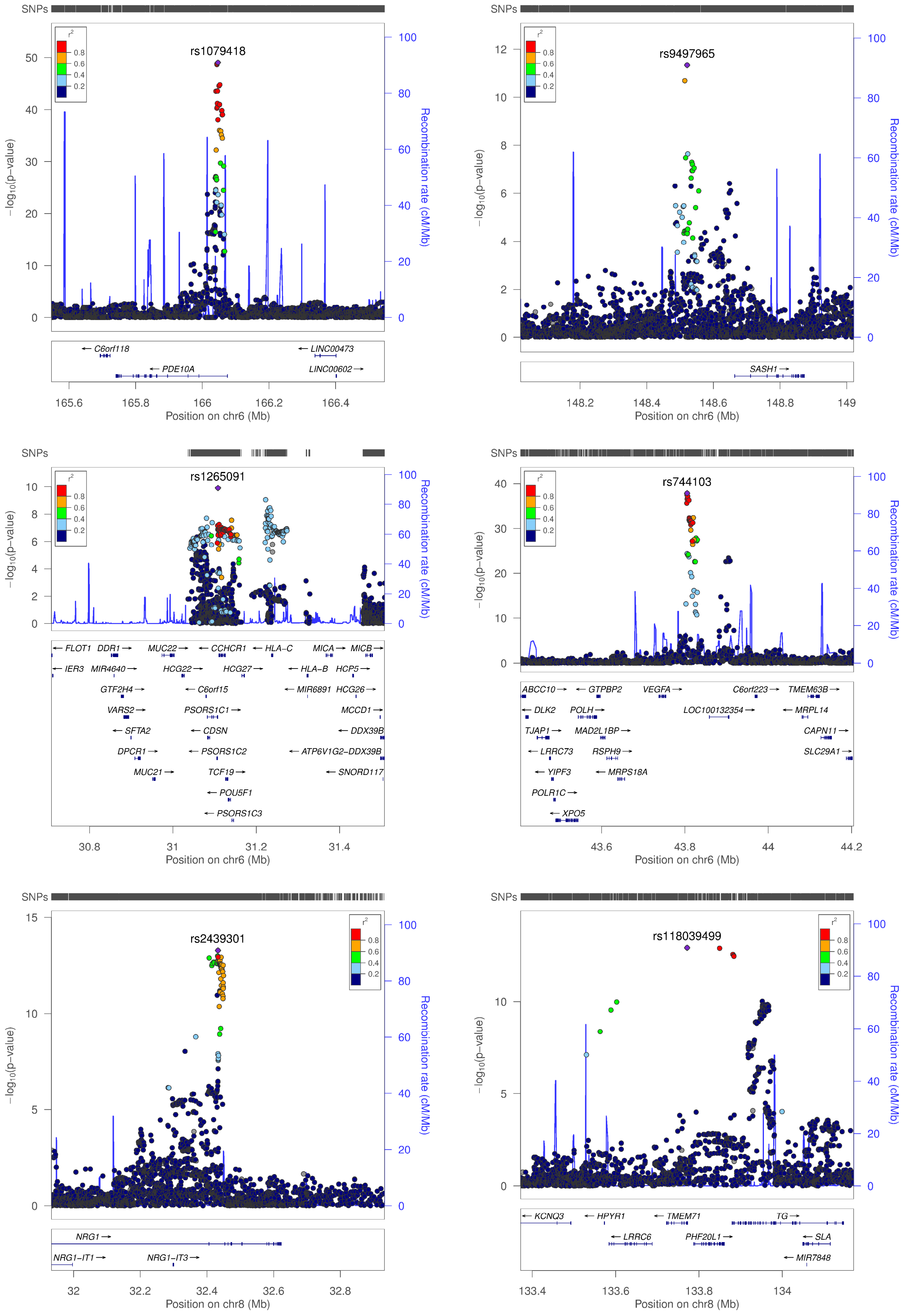

SNPs
rs1079418
r2
0.8
0.6
0.4
0.2
−log10(p−value)
Recombination rate (cM/Mb)
C6orf118
LINC00473
PDE10A
LINC00602
165.6
165.8
166
166.2
166.4
Position on chr6 (Mb)
rs9497965
SASH1
148.2
148.4
148.6
148.8
149
Position on chr6 (Mb)
rs1265091
FLOT1
DDR1
MUC22
CCHCR1
HLA−C
MICA
MICB
IER3
MIR4640
HCG22
HCG27
HLA−B
HCP5
GTF2H4
C6orf15
MIR6891
HCG26
VARS2
PSORS1C1
MCCD1
SFTA2
CDSN
DDX39B
DPCR1
PSORS1C2
ATP6V1G2−DDX39B
MUC21
TCF19
SNORD117
POU5F1
PSORS1C3
30.8
31
31.2
31.4
Position on chr6 (Mb)
rs744103
ABCC10
GTPBP2
VEGFA
C6orf223
TMEM63B
DLK2
POLH
LOC100132354
MRPL14
TJAP1
MAD2L1BP
CAPN11
LRRC73
RSPH9
SLC29A1
YIPF3
MRPS18A
POLR1C
XPO5
43.6
43.8
44
44.2
Position on chr6 (Mb)
rs2439301
NRG1
NRG1−IT1
NRG1−IT3
32
32.2
32.4
32.6
32.8
Position on chr8 (Mb)
rs118039499
KCNQ3
HPYR1
TMEM71
TG
LRRC6
PHF20L1
SLA
MIR7848
133.4
133.6
133.8
134
Position on chr8 (Mb)

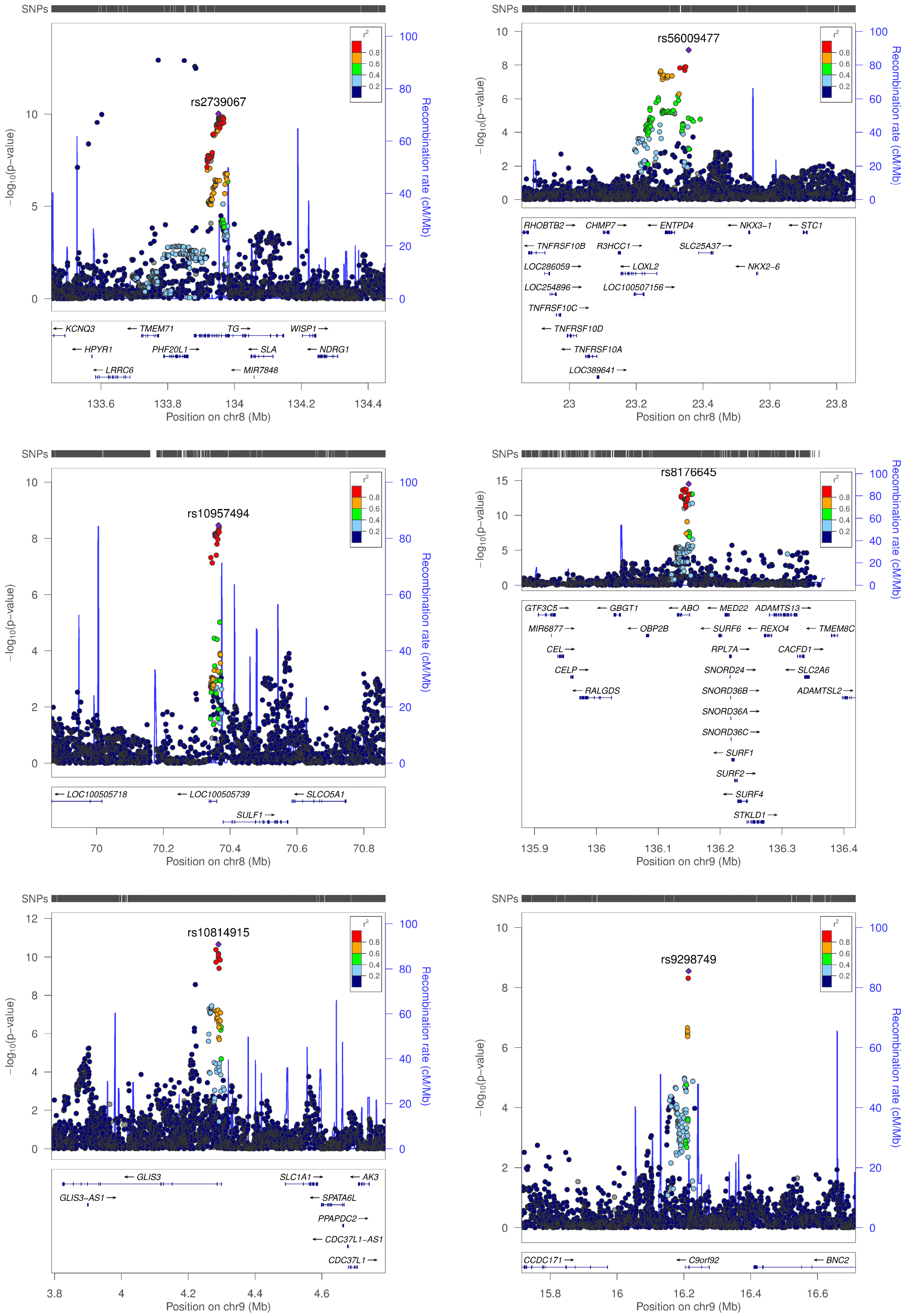
SNPs
rs2739067
−log10(p-value)
Recombination rate (cM/Mb)
KCNQ3
TMEM71
TG
WISP1
HPYR1
PHF20L1
SLA
NDRG1
LRRC6
MIR7848
Position on chr8 (Mb)
rs56009477
RHOBTB2
CHMP7
ENTPD4
NKX3−1
STC1
TNFRSF10B
R3HCC1
SLC25A37
LOC286059
LOXL2
NKX2−6
LOC254896
LOC100507156
TNFRSF10C
TNFRSF10D
TNFRSF10A
LOC389641
Position on chr8 (Mb)
rs10957494
LOC100505718
LOC100505739
SLCO5A1
SULF1
Position on chr8 (Mb)
rs8176645
GTF3C5
GBGT1
ABO
MED22
ADAMTS13
MIR6877
OBP2B
SURF6
REXO4
TMEM8C
CEL
RPL7A
CACFD1
CELP
SNORD24
SLC2A6
RALGDS
SNORD36B
ADAMTSL2
SNORD36A
SNORD36C
SURF1
SURF2
SURF4
STKLD1
Position on chr9 (Mb)
rs10814915
GLIS3
SLC1A1
AK3
GLIS3−AS1
SPATA6L
PPAPDC2
CDC37L1−AS1
CDC37L1
Position on chr9 (Mb)
rs9298749
CCDC171
C9orf92
BNC2
Position on chr9 (Mb)

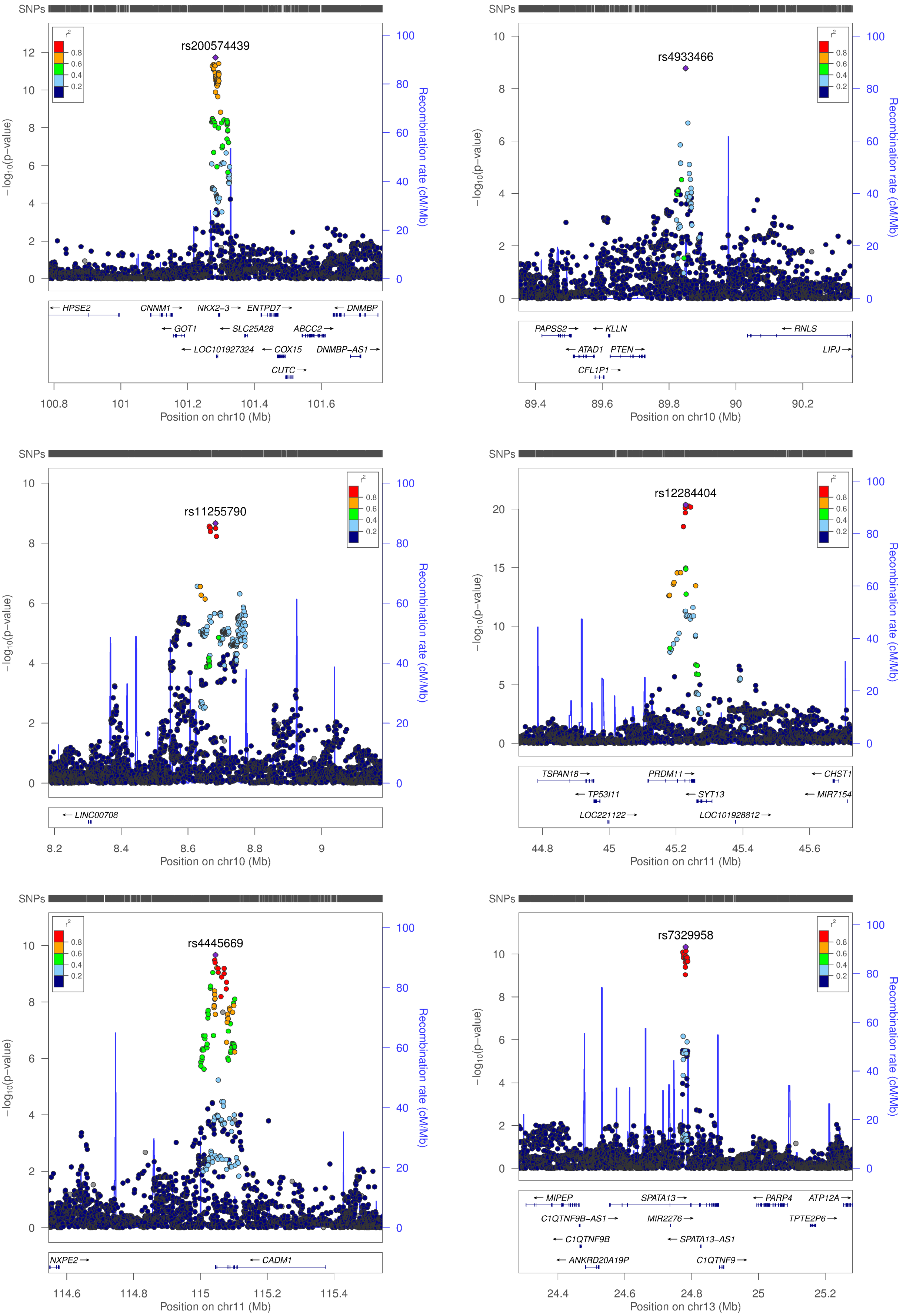

SNPs
rs200574439
HPSE2
CNNM1
NKX2-3
ENTPD7
DNMBP
GOT1
SLC25A28
ABCC2
LOC101927324
COX15
DNMBP-AS1
CUTC
Position on chr10 (Mb)
−log10(p−value)
Recombination rate (cM/Mb)
rs4933466
PAPSS2
KLLN
RNLS
ATAD1
PTEN
LIPJ
CFL1P1
Position on chr10 (Mb)
rs11255790
LINC00708
Position on chr10 (Mb)
rs12284404
TSPAN18
PRDM11
CHST1
TP53I11
SYT13
MIR7154
LOC221122
LOC101928812
Position on chr11 (Mb)
rs4445669
NXPE2
CADM1
Position on chr11 (Mb)
rs7329958
MIPEP
SPATA13
PARP4
ATP12A
C1QTNF9B-AS1
MIR2276
TPTE2P6
C1QTNF9B
SPATA13-AS1
ANKRD20A19P
C1QTNF9
Position on chr13 (Mb)

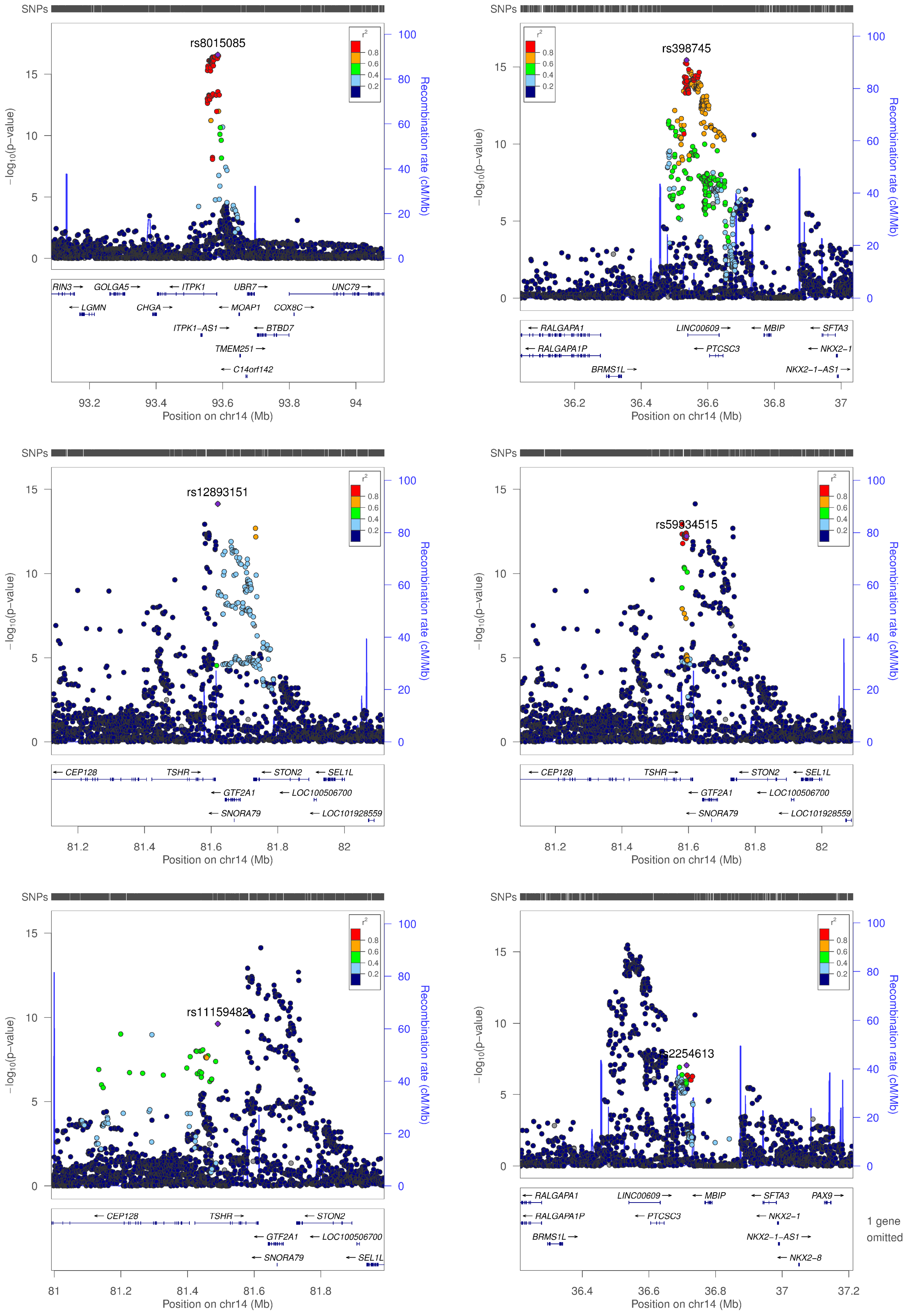
rs8015085
rs398745
rs12893151
rs59934515
rs11159482
rs2254613
SNPs
−log10(p−value)
Recombination rate (cM/Mb)
Position on chr14 (Mb)
RIN3
GOLGA5
ITPK1
UBR7
UNC79
LGMN
CHGA
MOAP1
COX8C
ITPK1−AS1
BTBD7
TMEM251
C14orf142
RALGAPA1
LINC00609
MBIP
SFTA3
RALGAPA1P
PTCSC3
NKX2−1
BRMS1L
NKX2−1−AS1
CEP128
TSHR
STON2
SEL1L
GTF2A1
LOC100506700
SNORA79
LOC101928559
PAX9
NKX2−8
1 gene omitted

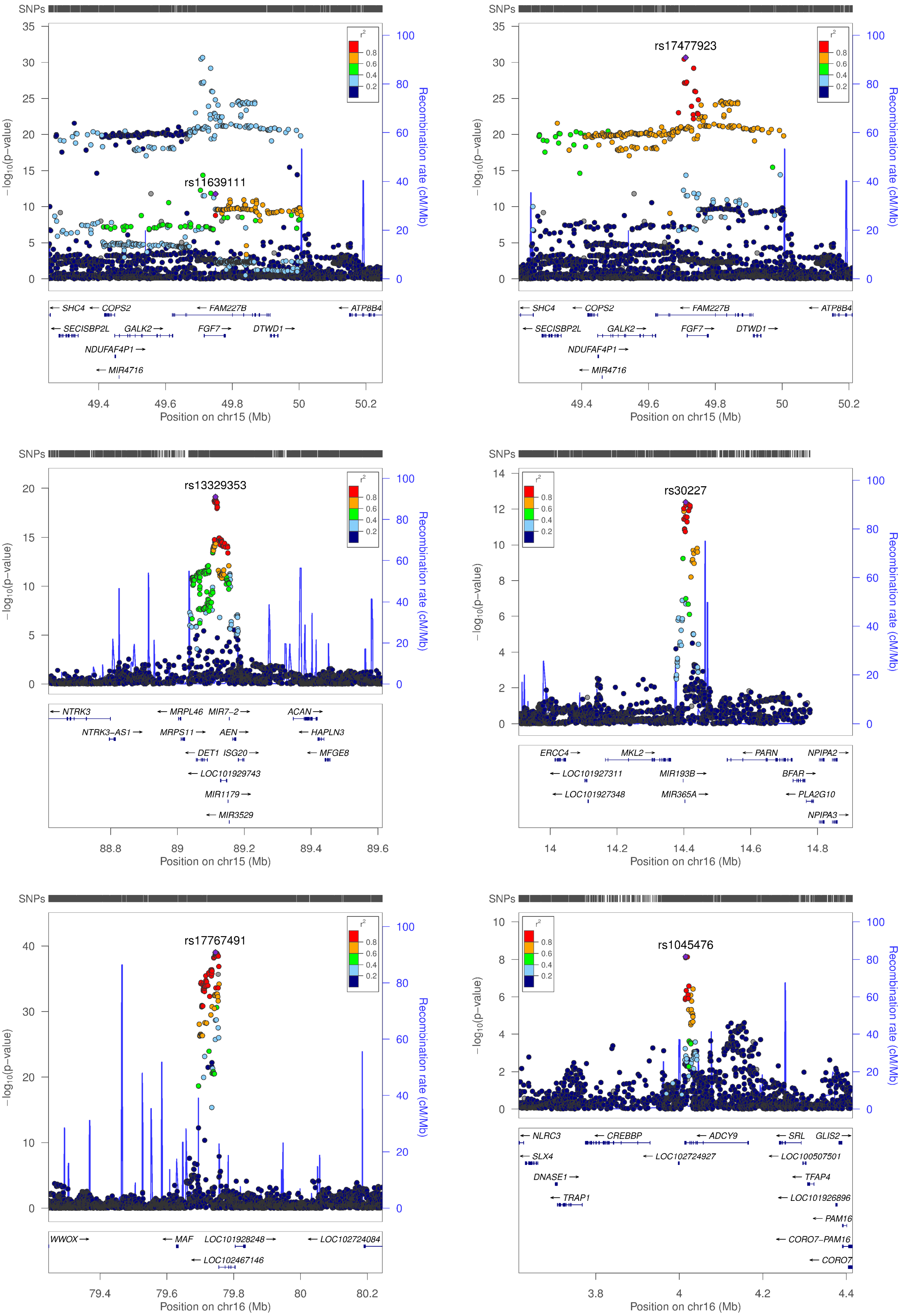

SNPs
−log10(p−value)
Recombination rate (cM/Mb)
r2
0.8
0.6
0.4
0.2
rs11639111
SHC4
COPS2
FAM227B
ATP8B4
SECISBP2L
GALK2
FGF7
DTWD1
NDUFAF4P1
MIR4716
Position on chr15 (Mb)
rs17477923
rs13329353
NTRK3
MRPL46
MIR7−2
ACAN
NTRK3−AS1
MRPS11
AEN
HAPLN3
DET1
ISG20
MFGE8
LOC101929743
MIR1179
MIR3529
rs30227
ERCC4
MKL2
PARN
NPIPA2
LOC101927311
MIR193B
BFAR
LOC101927348
MIR365A
PLA2G10
NPIPA3
Position on chr16 (Mb)
rs17767491
WWOX
MAF
LOC101928248
LOC102724084
LOC102467146
rs1045476
NLRC3
CREBBP
ADCY9
SRL
GLIS2
SLX4
LOC102724927
LOC100507501
DNASE1
TFAP4
TRAP1
LOC101926896
PAM16
CORO7−PAM16
CORO7

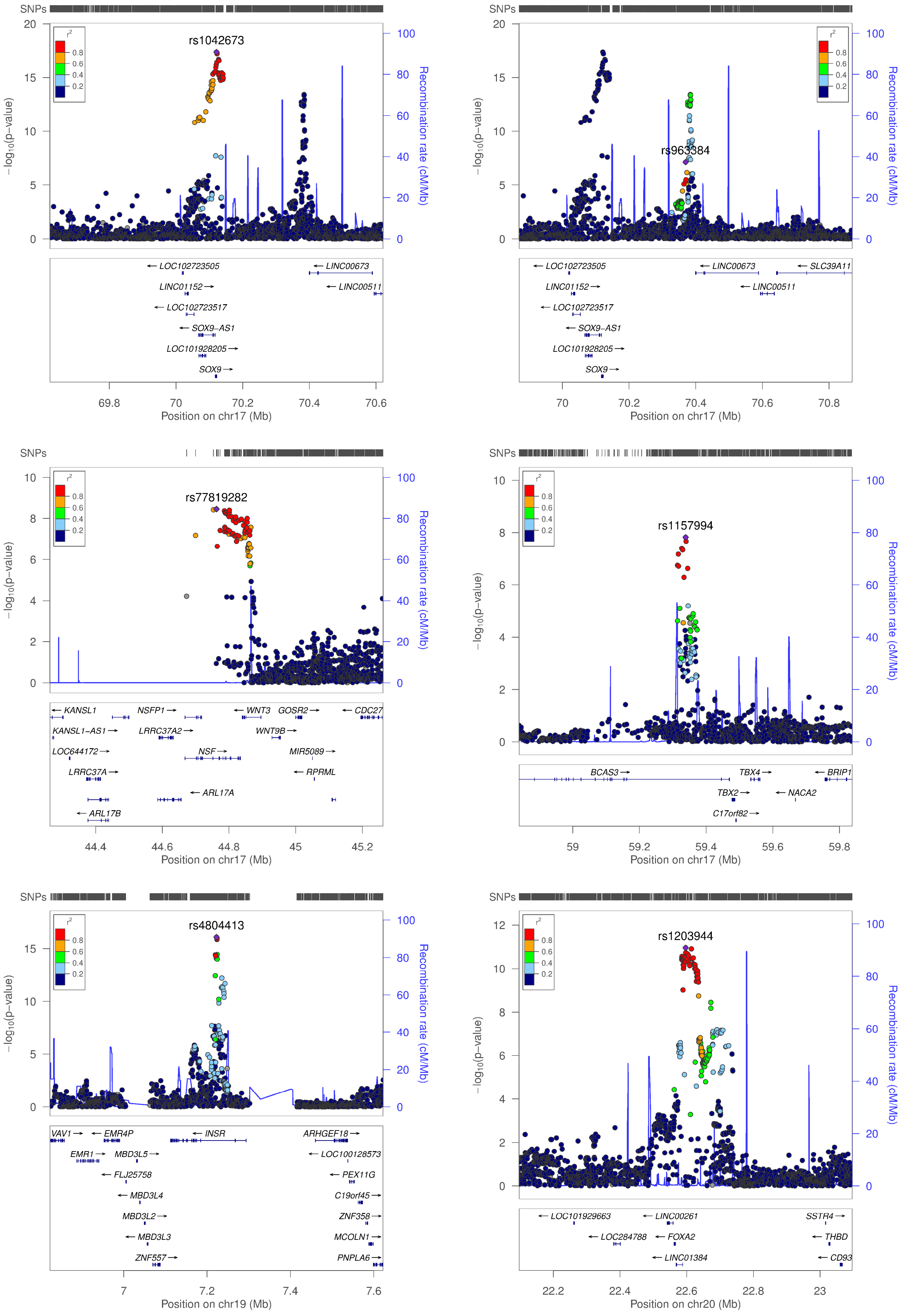

rs1042673
LOC102723505
LINC01152
LOC102723517
SOX9-AS1
LOC101928205
SOX9
LINC00673
LINC00511
Position on chr17 (Mb)
rs963384
SLC39A11
rs77819282
KANSL1
KANSL1-AS1
LOC644172
LRRC37A
ARL17B
NSFP1
LRRC37A2
NSF
ARL17A
WNT3
WNT9B
GOSR2
MIR5089
RPRML
CDC27
rs1157994
BCAS3
TBX4
TBX2
C17orf82
NACA2
BRIP1
rs4804413
VAV1
EMR4P
EMR1
MBD3L5
FLJ25758
MBD3L4
MBD3L2
MBD3L3
ZNF557
INSR
ARHGEF18
LOC100128573
PEX11G
C19orf45
ZNF358
MCOLN1
PNPLA6
Position on chr19 (Mb)
rs1203944
LOC101929663
LOC284788
LINC00261
FOXA2
LINC01384
SSTR4
THBD
CD93
Position on chr20 (Mb)
SNPs
$-\log_{10}$(p-value)
Recombination rate (cM/Mb)
r^2

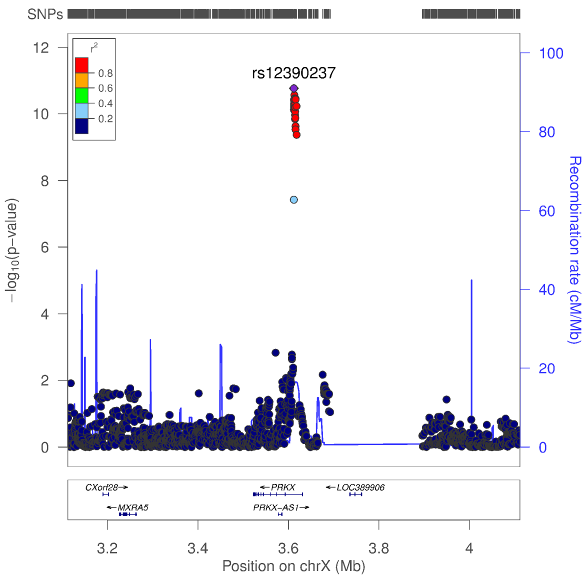

Regional association plots are shown for all genome-wide significant independent SNPs after the final stage meta-analyses. Correlation with the index SNP is estimated based on the 1000 Genomes reference samples. Plots were generated using the stand-alone version of LocusZoom[5]. Genetic positions refer to GRCh37/hg19 coordinates.

Supplementary Figure 3: Regional association plots for FT4

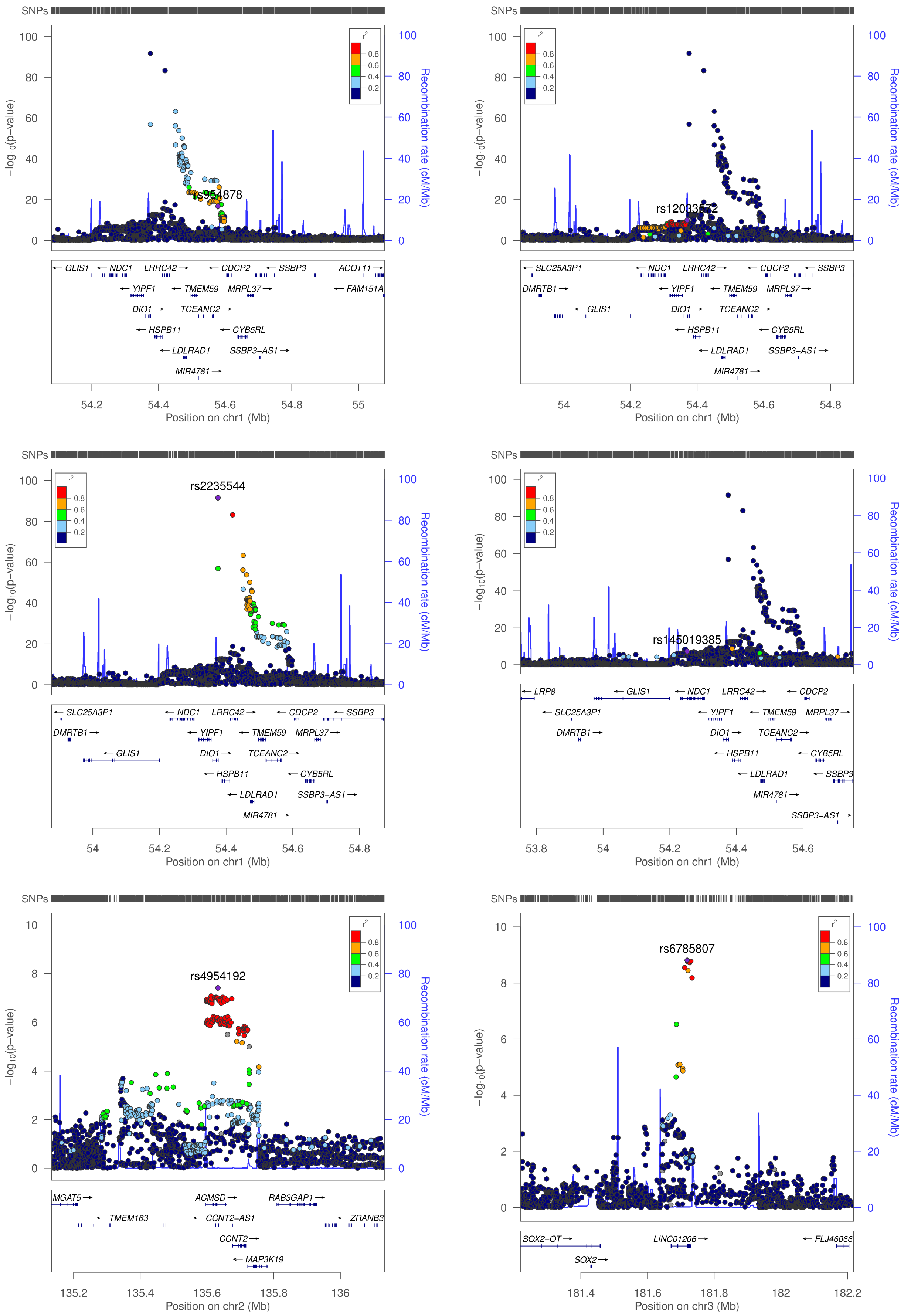

SNPs
r2
0.8
0.6
0.4
0.2
−log10(p-value)
Recombination rate (cM/Mb)
rs964878
GLIS1
NDC1
LRRC42
CDCP2
SSBP3
ACOT11
YIPF1
TMEM59
MRPL37
FAM151A
DIO1
TCEANC2
HSPB11
CYB5RL
LDLRAD1
SSBP3−AS1
MIR4781
Position on chr1 (Mb)
rs12033572
SLC25A3P1
DMRTB1
rs2235544
rs145019385
LRP8
rs4954192
MGAT5
ACMSD
RAB3GAP1
TMEM163
CCNT2−AS1
ZRANB3
CCNT2
MAP3K19
Position on chr2 (Mb)
rs6785807
SOX2−OT
LINC01206
FLJ46066
SOX2
Position on chr3 (Mb)

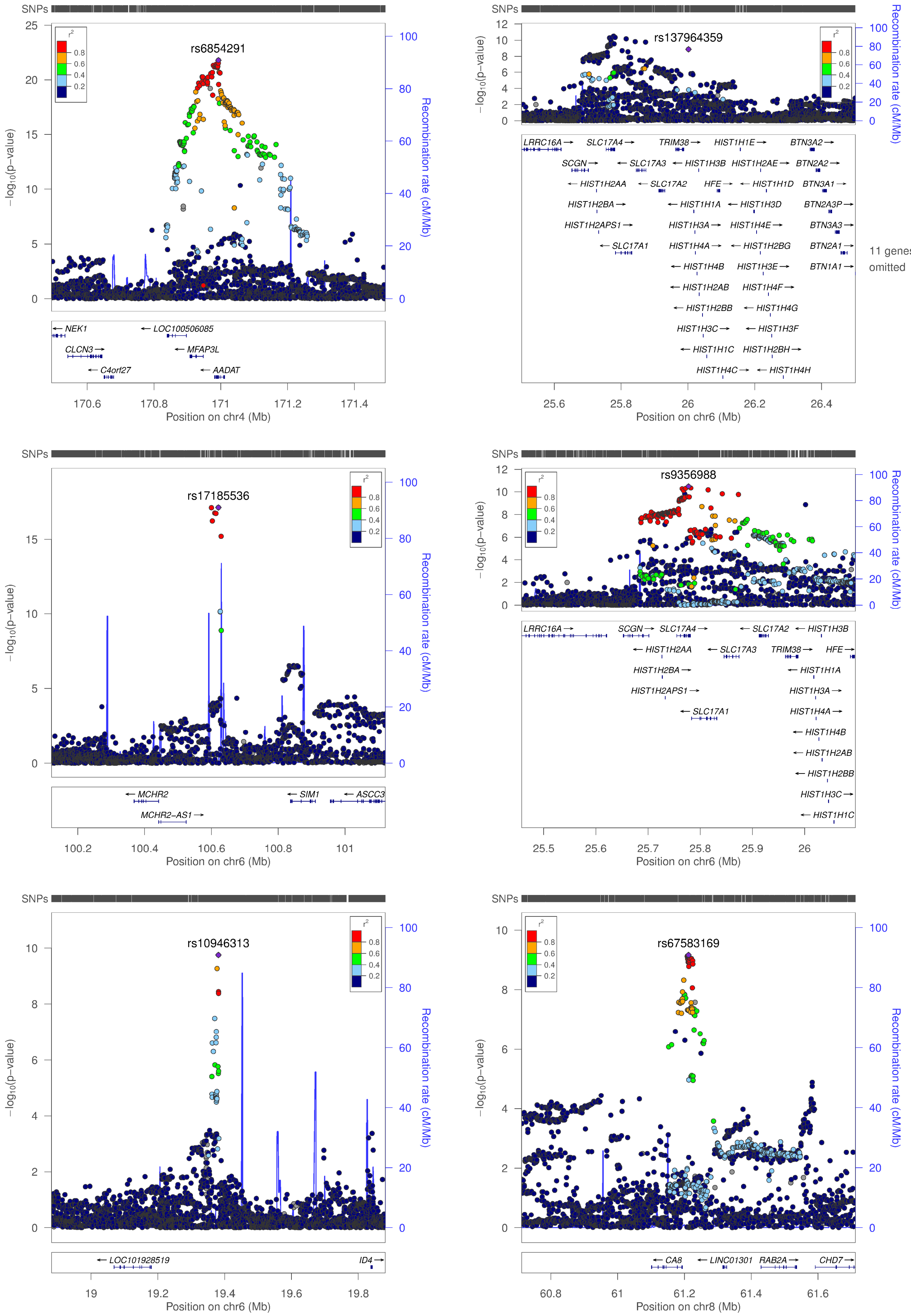

SNPs
rs6854291
−log10(p−value)
Recombination rate (cM/Mb)
r2
0.8
0.6
0.4
0.2
NEK1
LOC100506085
CLCN3
MFAP3L
C4orf27
AADAT
170.6
170.8
171
171.2
171.4
Position on chr4 (Mb)
rs137964359
LRRC16A
SLC17A4
TRIM38
HIST1H1E
BTN3A2
SCGN
SLC17A3
HIST1H3B
HIST1H2AE
BTN2A2
HIST1H2AA
SLC17A2
HFE
HIST1H1D
BTN3A1
HIST1H2BA
HIST1H1A
HIST1H3D
BTN2A3P
HIST1H2APS1
HIST1H3A
HIST1H4E
BTN3A3
SLC17A1
HIST1H4A
HIST1H2BG
BTN2A1
HIST1H4B
HIST1H3E
BTN1A1
HIST1H2AB
HIST1H4F
HIST1H2BB
HIST1H4G
HIST1H3C
HIST1H3F
HIST1H1C
HIST1H2BH
HIST1H4C
HIST1H4H
11 genes omitted
25.6
25.8
26
26.2
26.4
Position on chr6 (Mb)
rs17185536
MCHR2
SIM1
ASCC3
MCHR2−AS1
100.2
100.4
100.6
100.8
101
Position on chr6 (Mb)
rs9356988
25.5
25.6
25.7
25.8
25.9
26
Position on chr6 (Mb)
rs10946313
LOC101928519
ID4
19
19.2
19.4
19.6
19.8
Position on chr6 (Mb)
rs67583169
CA8
LINC01301
RAB2A
CHD7
60.8
61
61.2
61.4
61.6
Position on chr8 (Mb)

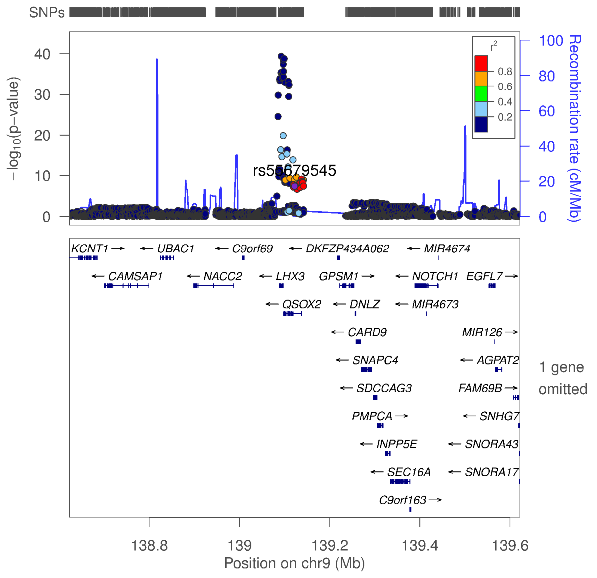
SNPs
rs58679545
−log10(p−value)
Recombination rate (cM/Mb)
r2
0.8
0.6
0.4
0.2
KCNT1
UBAC1
C9orf69
DKFZP434A062
MIR4674
CAMSAP1
NACC2
LHX3
GPSM1
NOTCH1
EGFL7
QSOX2
DNLZ
MIR4673
CARD9
MIR126
SNAPC4
AGPAT2
SDCCAG3
FAM69B
PMPCA
SNHG7
INPP5E
SNORA43
SEC16A
SNORA17
C9orf163
1 gene omitted
Position on chr9 (Mb)

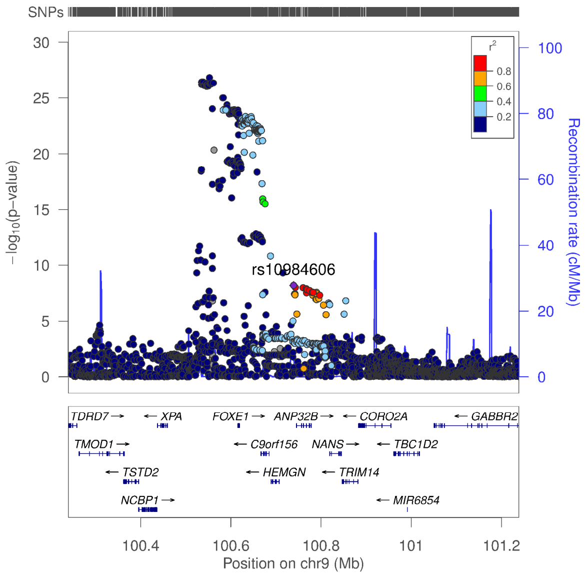
SNPs
rs10984606
−log10(p−value)
Recombination rate (cM/Mb)
r2
TDRD7
XPA
FOXE1
ANP32B
CORO2A
GABBR2
TMOD1
C9orf156
NANS
TBC1D2
TSTD2
HEMGN
TRIM14
NCBP1
MIR6854
Position on chr9 (Mb)

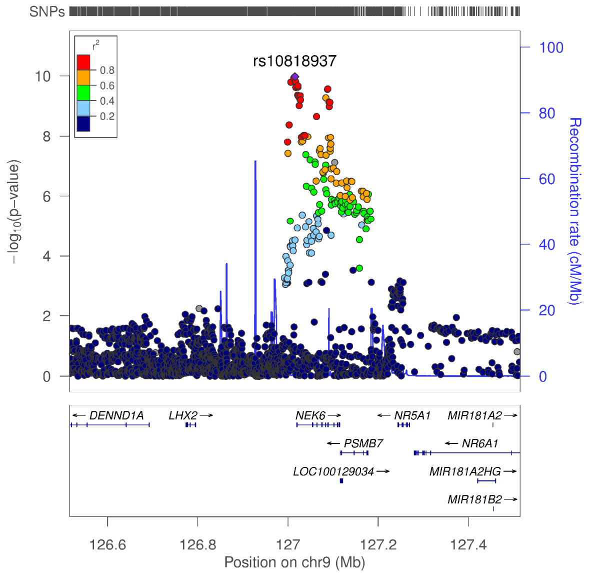
SNPs
rs10818937
−log10(p−value)
Recombination rate (cM/Mb)
r2
DENND1A
LHX2
NEK6
NR5A1
MIR181A2
PSMB7
NR6A1
LOC100129034
MIR181A2HG
MIR181B2
Position on chr9 (Mb)

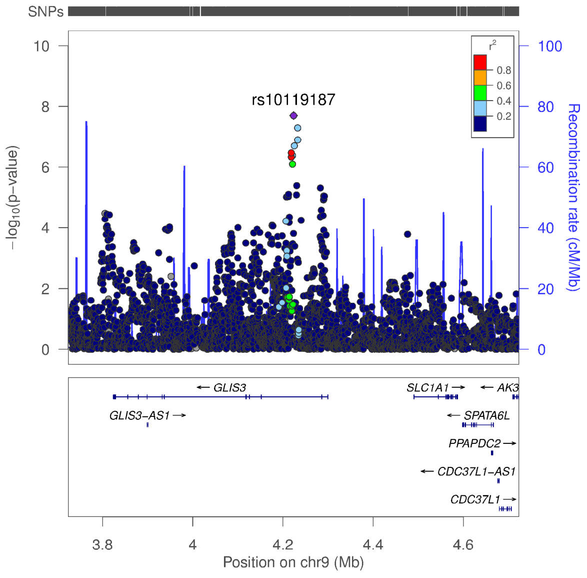
SNPs
rs10119187
−log10(p−value)
Recombination rate (cM/Mb)
r2
GLIS3
SLC1A1
AK3
GLIS3−AS1
SPATA6L
PPAPDC2
CDC37L1−AS1
CDC37L1
Position on chr9 (Mb)

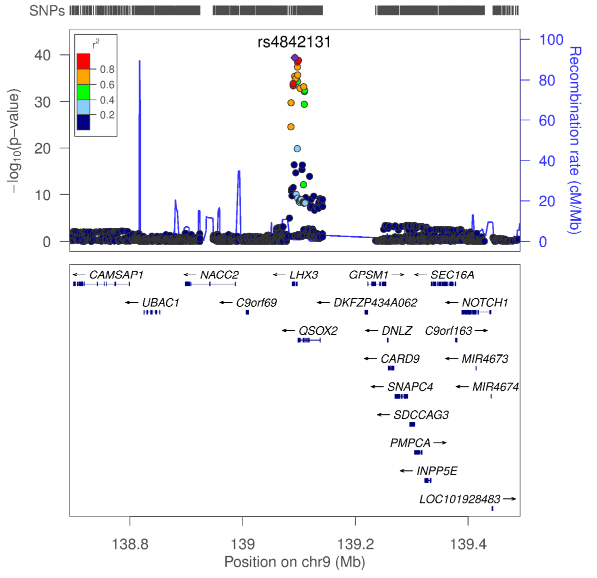
SNPs
rs4842131
−log10(p−value)
Recombination rate (cM/Mb)
r2
CAMSAP1
NACC2
LHX3
GPSM1
SEC16A
UBAC1
C9orf69
DKFZP434A062
NOTCH1
QSOX2
DNLZ
C9orf163
CARD9
MIR4673
SNAPC4
MIR4674
SDCCAG3
PMPCA
INPP5E
LOC101928483
Position on chr9 (Mb)

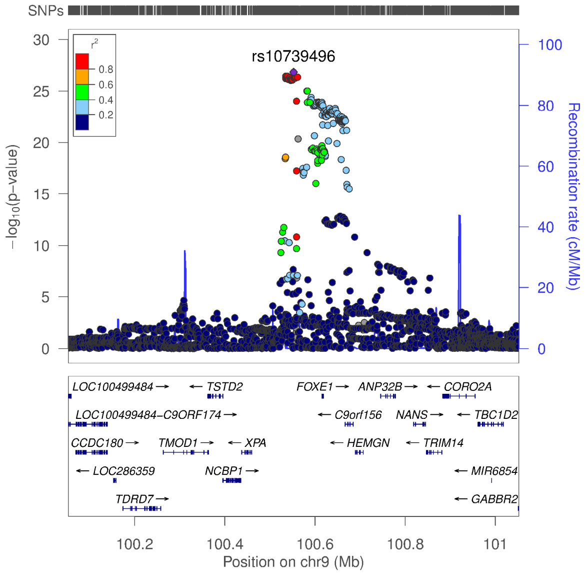
SNPs
rs10739496
−log10(p−value)
Recombination rate (cM/Mb)
r2
LOC100499484
TSTD2
FOXE1
ANP32B
CORO2A
LOC100499484−C9ORF174
C9orf156
NANS
TBC1D2
CCDC180
TMOD1
XPA
HEMGN
TRIM14
LOC286359
NCBP1
MIR6854
TDRD7
GABBR2
Position on chr9 (Mb)

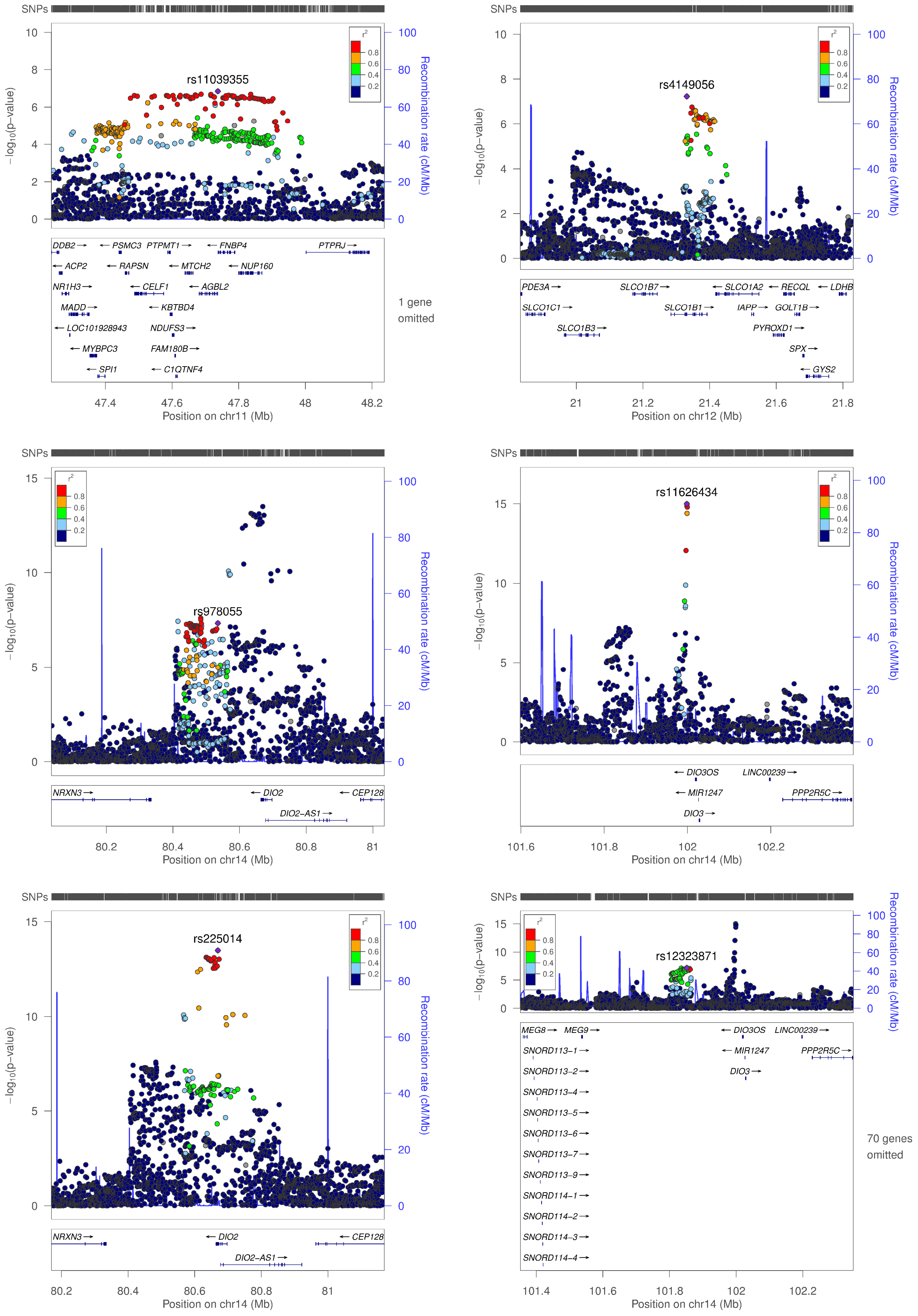

SNPs
−log10(p−value)
Recombination rate (cM/Mb)
r2
0.8
0.6
0.4
0.2
rs11039355
DDB2 →
← PSMC3
PTPMT1 →
← FNBP4
PTPRJ →
← ACP2
← RAPSN
← MTCH2
← NUP160
NR1H3 →
← CELF1
← AGBL2
MADD →
← KBTBD4
← LOC101928943
NDUFS3 →
← MYBPC3
FAM180B →
← SPI1
← C1QTNF4
1 gene omitted
Position on chr11 (Mb)
rs4149056
PDE3A →
SLCO1B7 →
← SLCO1A2
← RECQL
← LDHB
SLCO1C1 →
SLCO1B1 →
IAPP →
GOLT1B →
SLCO1B3 →
PYROXD1 →
SPX →
← GYS2
Position on chr12 (Mb)
rs978055
NRXN3 →
← DIO2
← CEP128
DIO2−AS1 →
Position on chr14 (Mb)
rs11626434
← DIO3OS
LINC00239 →
← MIR1247
PPP2R5C →
DIO3 →
Position on chr14 (Mb)
rs225014
NRXN3 →
← DIO2
← CEP128
DIO2−AS1 →
Position on chr14 (Mb)
rs12323871
MEG8 →
MEG9 →
← DIO3OS
LINC00239 →
SNORD113−1 →
← MIR1247
PPP2R5C →
SNORD113−2 →
DIO3 →
SNORD113−4 →
SNORD113−5 →
SNORD113−6 →
SNORD113−7 →
SNORD113−9 →
SNORD114−1 →
SNORD114−2 →
SNORD114−3 →
SNORD114−4 →
70 genes omitted
Position on chr14 (Mb)

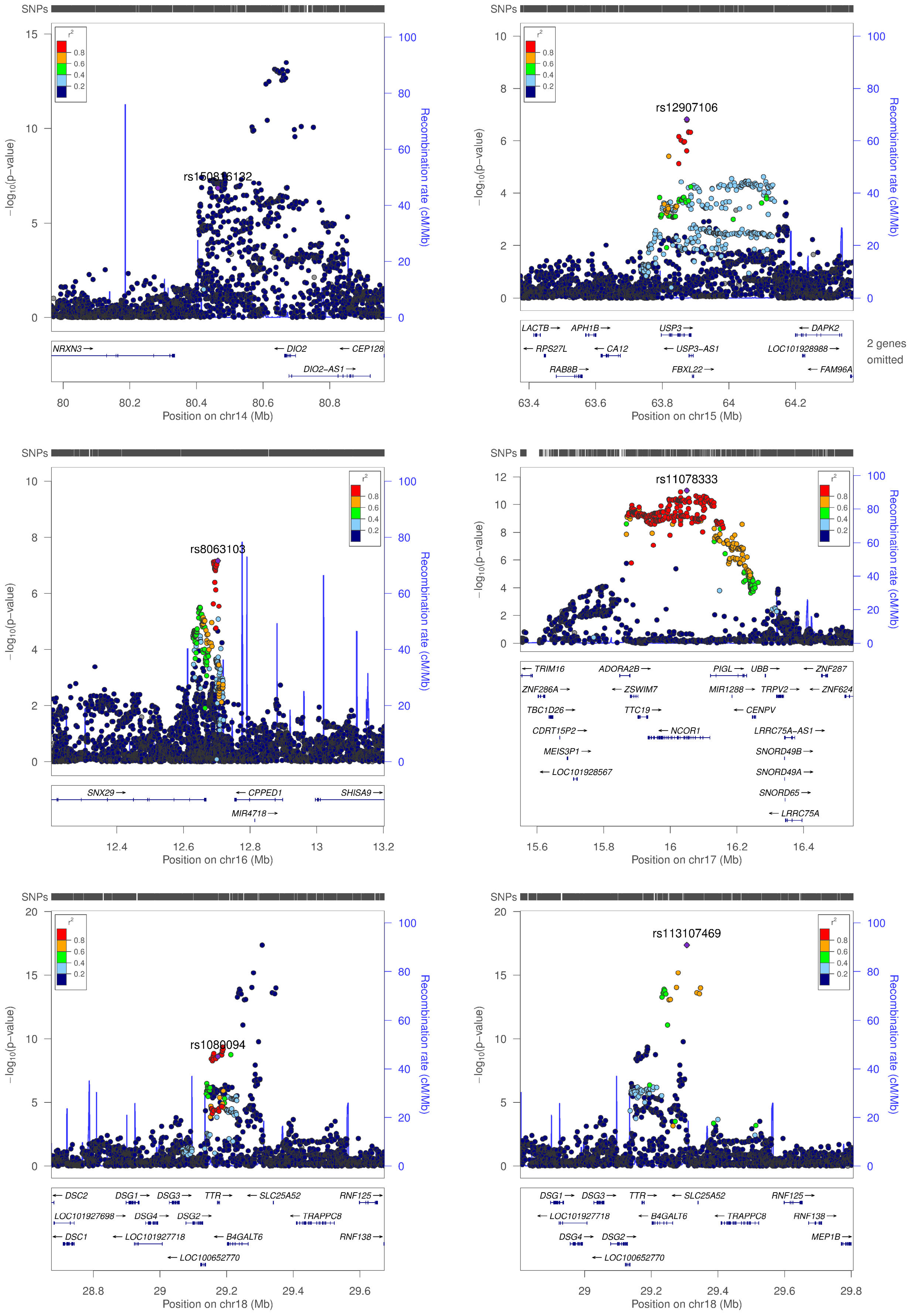

SNPs
−log10(p−value)
Recombination rate (cM/Mb)
r2
0.8
0.6
0.4
0.2
rs150816132
NRXN3
DIO2
CEP128
DIO2−AS1
Position on chr14 (Mb)
rs12907106
LACTB
APH1B
USP3
DAPK2
RPS27L
CA12
USP3−AS1
LOC101928988
RAB8B
FBXL22
FAM96A
2 genes omitted
Position on chr15 (Mb)
rs8063103
SNX29
CPPED1
SHISA9
MIR4718
Position on chr16 (Mb)
rs11078333
TRIM16
ADORA2B
PIGL
UBB
ZNF287
ZNF286A
ZSWIM7
MIR1288
TRPV2
ZNF624
TBC1D26
TTC19
CENPV
CDRT15P2
NCOR1
LRRC75A−AS1
MEIS3P1
SNORD49B
LOC101928567
SNORD49A
SNORD65
LRRC75A
Position on chr17 (Mb)
rs1080094
DSC2
DSG1
DSG3
TTR
SLC25A52
RNF125
LOC101927698
DSG4
DSG2
TRAPPC8
DSC1
LOC101927718
B4GALT6
RNF138
LOC100652770
Position on chr18 (Mb)
rs113107469
MEP1B
Position on chr18 (Mb)

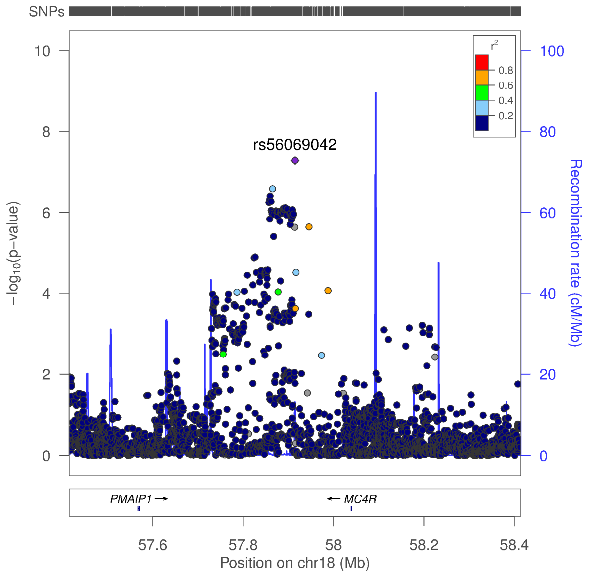

Regional association plots are shown for all genome-wide significant independent SNPs after the final stage meta-analyses. Correlation with the index SNP is estimated based on the 1000 Genomes reference samples. Plots were generated using the stand-alone version of LocusZoom[5]. Genetic positions refer to GRCh37/hg19 coordinates.

Supplementary Figure 4: Forest plots for TSH

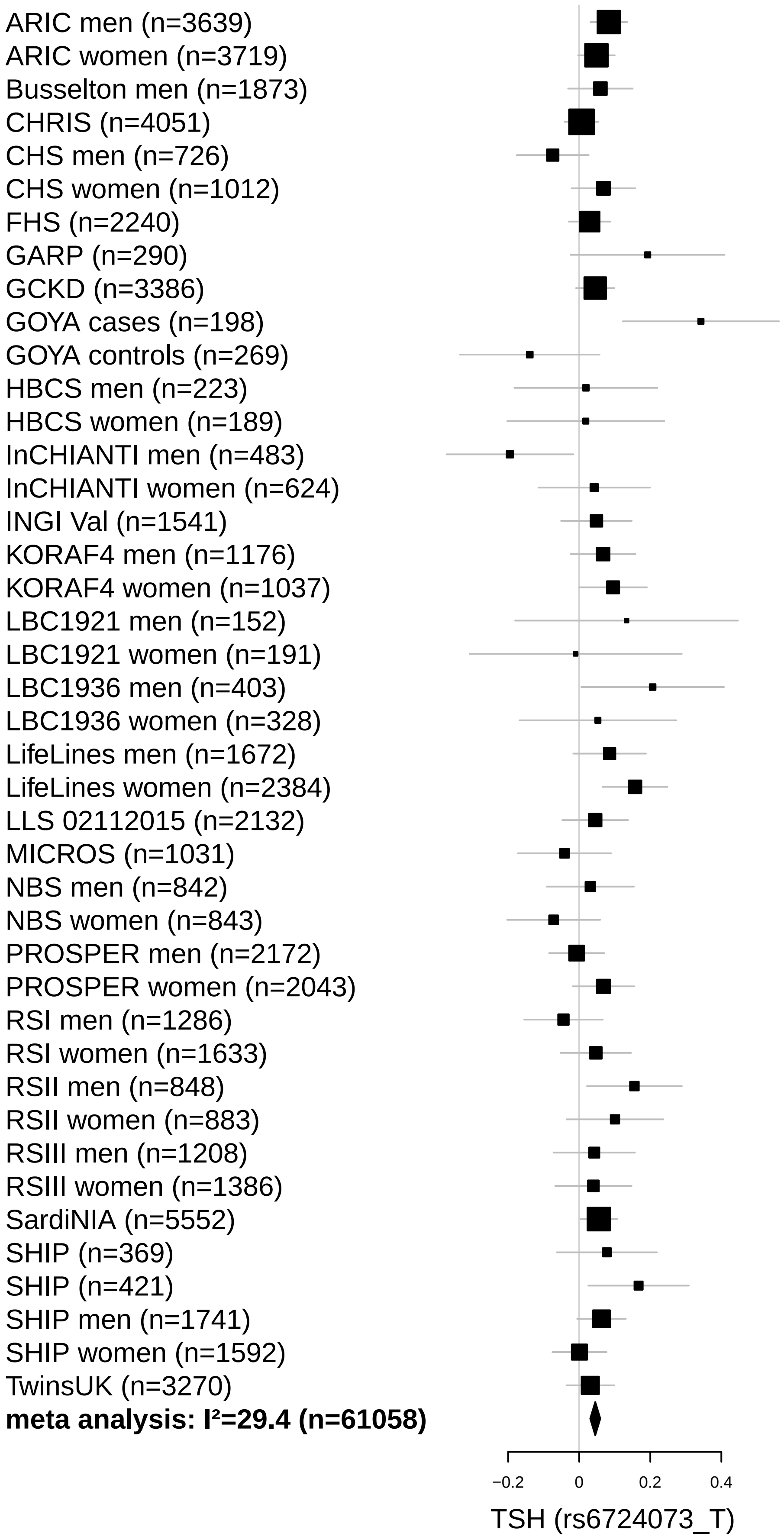
ARIC men (n=3639)
ARIC women (n=3719)
Busselton men (n=1873)
CHRIS (n=4051)
CHS men (n=726)
CHS women (n=1012)
FHS (n=2240)
GARP (n=290)
GCKD (n=3386)
GOYA cases (n=198)
GOYA controls (n=269)
HBCS men (n=223)
HBCS women (n=189)
InCHIANTI men (n=483)
InCHIANTI women (n=624)
INGI Val (n=1541)
KORAF4 men (n=1176)
KORAF4 women (n=1037)
LBC1921 men (n=152)
LBC1921 women (n=191)
LBC1936 men (n=403)
LBC1936 women (n=328)
LifeLines men (n=1672)
LifeLines women (n=2384)
LLS 02112015 (n=2132)
MICROS (n=1031)
NBS men (n=842)
NBS women (n=843)
PROSPER men (n=2172)
PROSPER women (n=2043)
RSI men (n=1286)
RSI women (n=1633)
RSII men (n=848)
RSII women (n=883)
RSIII men (n=1208)
RSIII women (n=1386)
SardiNIA (n=5552)
SHIP (n=369)
SHIP (n=421)
SHIP men (n=1741)
SHIP women (n=1592)
TwinsUK (n=3270)
meta analysis: I²=29.4 (n=61058)
−0.2
0
0.2
0.4
TSH (rs6724073_T)

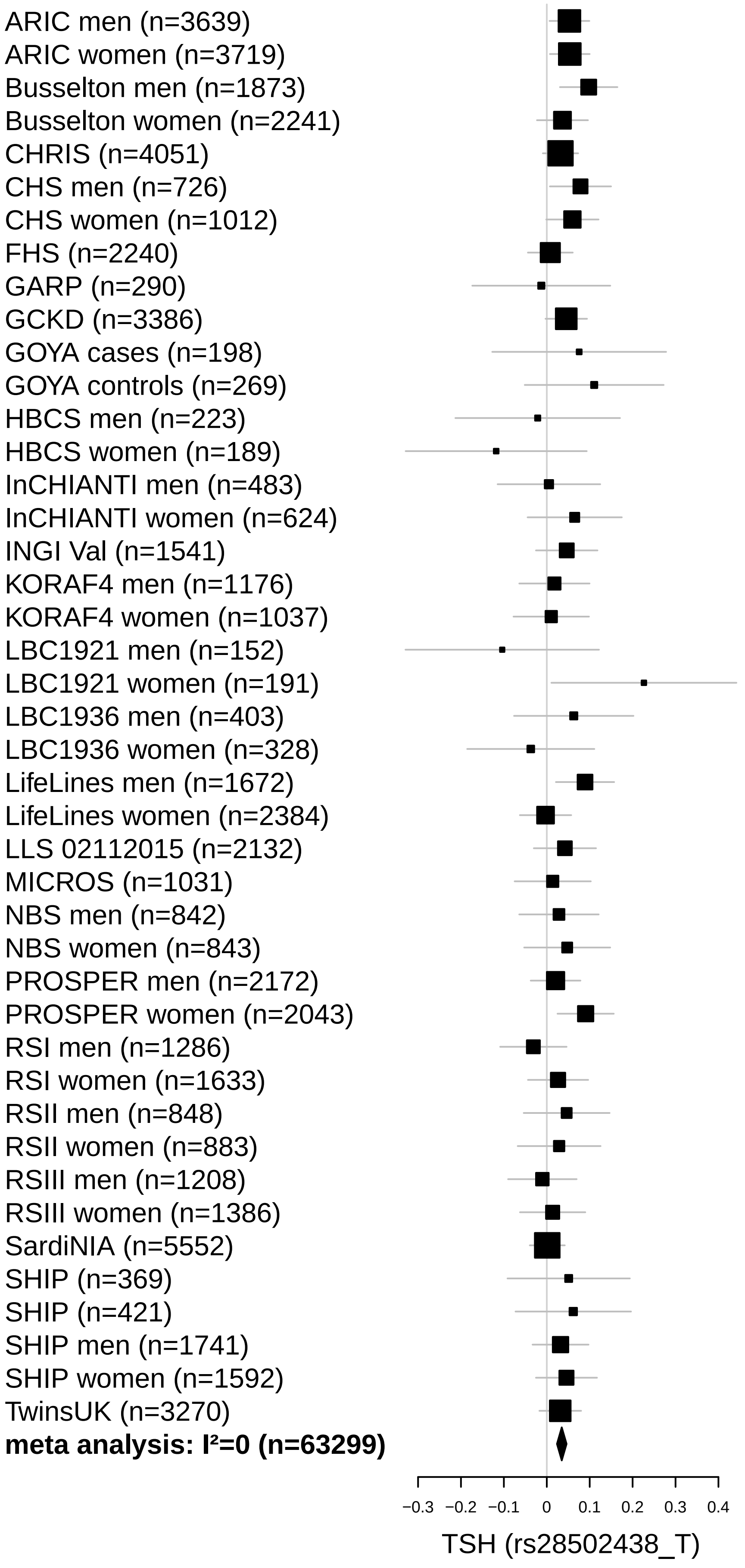

ARIC men (n=3639)
ARIC women (n=3719)
Busselton men (n=1873)
Busselton women (n=2241)
CHRIS (n=4051)
CHS men (n=726)
CHS women (n=1012)
FHS (n=2240)
GARP (n=290)
GCKD (n=3386)
GOYA cases (n=198)
GOYA controls (n=269)
HBCS men (n=223)
HBCS women (n=189)
InCHIANTI men (n=483)
InCHIANTI women (n=624)
INGI Val (n=1541)
KORAF4 men (n=1176)
KORAF4 women (n=1037)
LBC1921 men (n=152)
LBC1921 women (n=191)
LBC1936 men (n=403)
LBC1936 women (n=328)
LifeLines men (n=1672)
LifeLines women (n=2384)
LLS 02112015 (n=2132)
MICROS (n=1031)
NBS men (n=842)
NBS women (n=843)
PROSPER men (n=2172)
PROSPER women (n=2043)
RSI men (n=1286)
RSI women (n=1633)
RSII men (n=848)
RSII women (n=883)
RSIII men (n=1208)
RSIII women (n=1386)
SardiNIA (n=5552)
SHIP (n=369)
SHIP (n=421)
SHIP men (n=1741)
SHIP women (n=1592)
TwinsUK (n=3270)
meta analysis: I²=0 (n=63299)
−0.3
−0.2
−0.1
0
0.1
0.2
0.3
0.4
TSH (rs28502438_T)

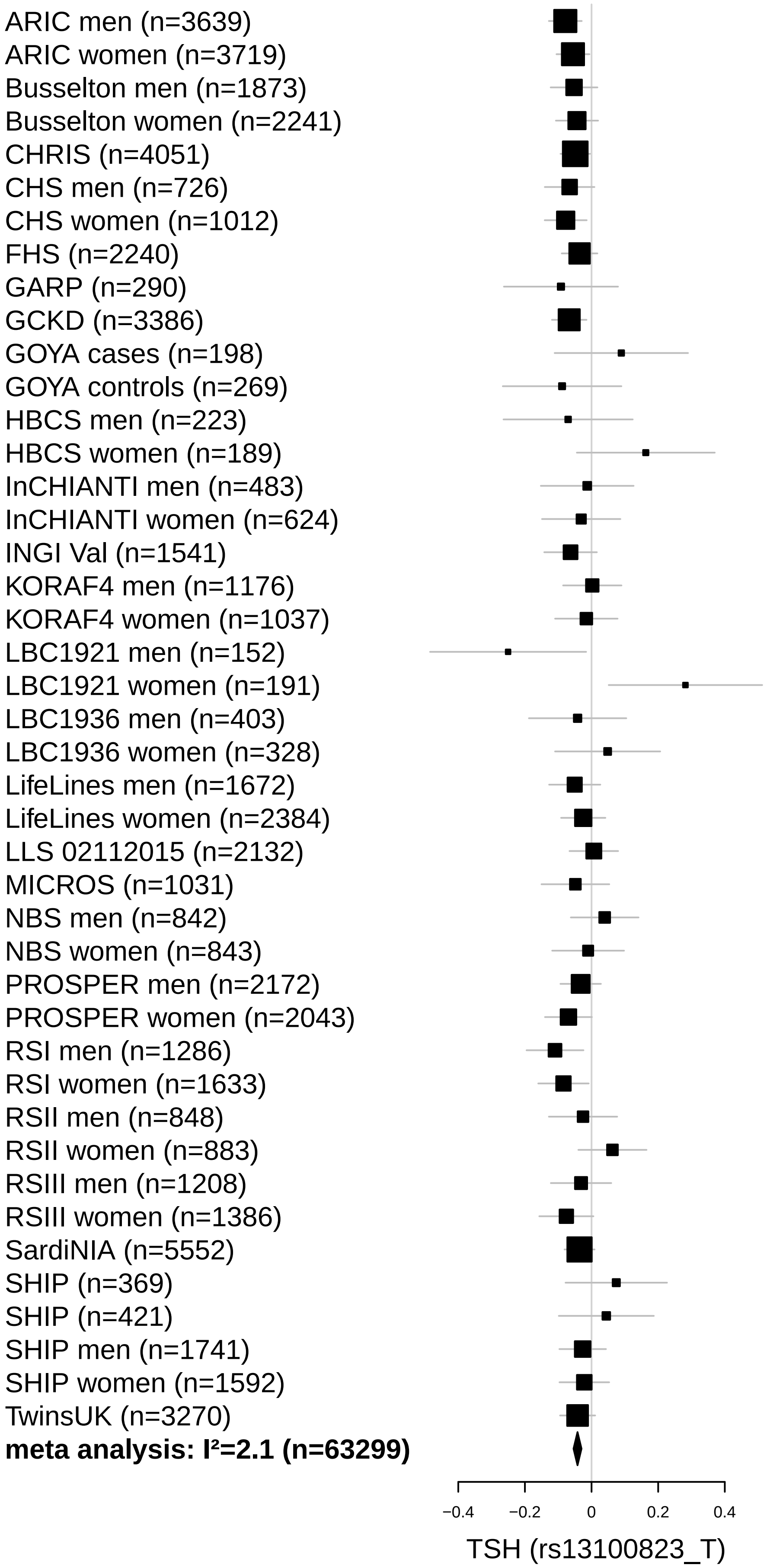

ARIC men (n=3639)
ARIC women (n=3719)
Busselton men (n=1873)
Busselton women (n=2241)
CHRIS (n=4051)
CHS men (n=726)
CHS women (n=1012)
FHS (n=2240)
GARP (n=290)
GCKD (n=3386)
GOYA cases (n=198)
GOYA controls (n=269)
HBCS men (n=223)
HBCS women (n=189)
InCHIANTI men (n=483)
InCHIANTI women (n=624)
INGI Val (n=1541)
KORAF4 men (n=1176)
KORAF4 women (n=1037)
LBC1921 men (n=152)
LBC1921 women (n=191)
LBC1936 men (n=403)
LBC1936 women (n=328)
LifeLines men (n=1672)
LifeLines women (n=2384)
LLS 02112015 (n=2132)
MICROS (n=1031)
NBS men (n=842)
NBS women (n=843)
PROSPER men (n=2172)
PROSPER women (n=2043)
RSI men (n=1286)
RSI women (n=1633)
RSII men (n=848)
RSII women (n=883)
RSIII men (n=1208)
RSIII women (n=1386)
SardiNIA (n=5552)
SHIP (n=369)
SHIP (n=421)
SHIP men (n=1741)
SHIP women (n=1592)
TwinsUK (n=3270)
meta analysis: I²=2.1 (n=63299)
−0.4
−0.2
0
0.2
0.4
TSH (rs13100823_T)

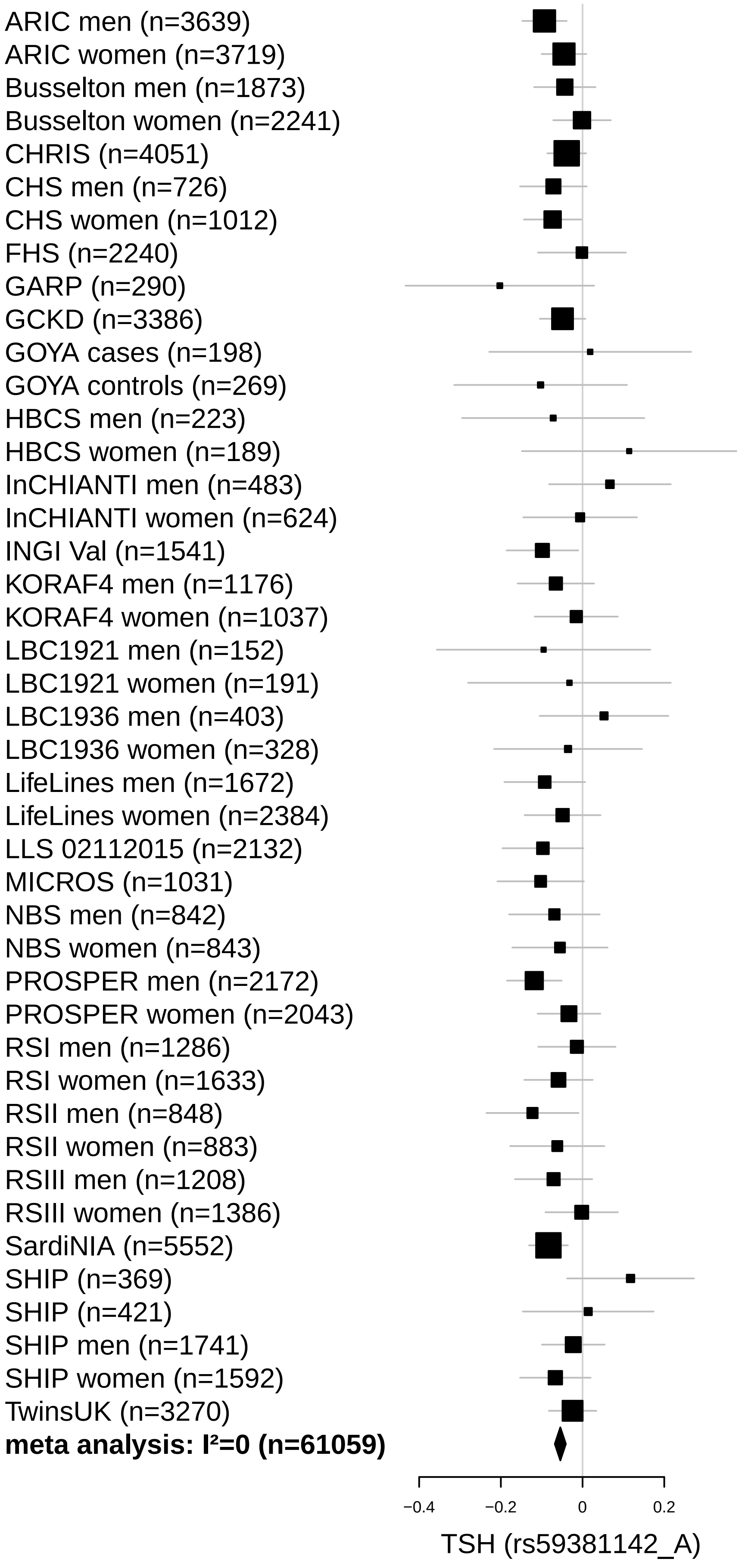
ARIC men (n=3639)
ARIC women (n=3719)
Busselton men (n=1873)
Busselton women (n=2241)
CHRIS (n=4051)
CHS men (n=726)
CHS women (n=1012)
FHS (n=2240)
GARP (n=290)
GCKD (n=3386)
GOYA cases (n=198)
GOYA controls (n=269)
HBCS men (n=223)
HBCS women (n=189)
InCHIANTI men (n=483)
InCHIANTI women (n=624)
INGI Val (n=1541)
KORAF4 men (n=1176)
KORAF4 women (n=1037)
LBC1921 men (n=152)
LBC1921 women (n=191)
LBC1936 men (n=403)
LBC1936 women (n=328)
LifeLines men (n=1672)
LifeLines women (n=2384)
LLS 02112015 (n=2132)
MICROS (n=1031)
NBS men (n=842)
NBS women (n=843)
PROSPER men (n=2172)
PROSPER women (n=2043)
RSI men (n=1286)
RSI women (n=1633)
RSII men (n=848)
RSII women (n=883)
RSIII men (n=1208)
RSIII women (n=1386)
SardiNIA (n=5552)
SHIP (n=369)
SHIP (n=421)
SHIP men (n=1741)
SHIP women (n=1592)
TwinsUK (n=3270)
meta analysis: I²=0 (n=61059)
−0.4
−0.2
0
0.2
TSH (rs59381142_A)

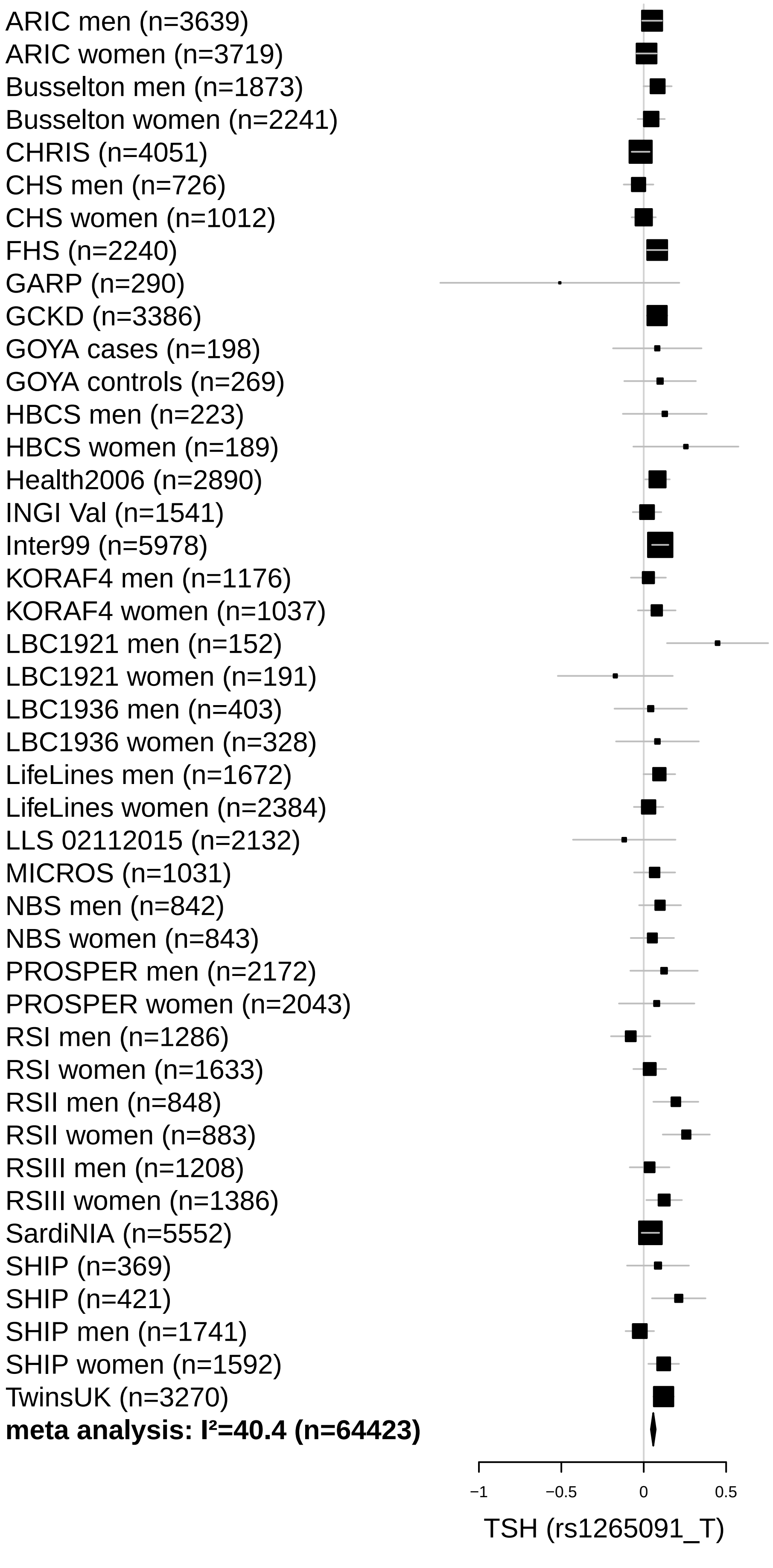
ARIC men (n=3639)
ARIC women (n=3719)
Busselton men (n=1873)
Busselton women (n=2241)
CHRIS (n=4051)
CHS men (n=726)
CHS women (n=1012)
FHS (n=2240)
GARP (n=290)
GCKD (n=3386)
GOYA cases (n=198)
GOYA controls (n=269)
HBCS men (n=223)
HBCS women (n=189)
Health2006 (n=2890)
INGI Val (n=1541)
Inter99 (n=5978)
KORAF4 men (n=1176)
KORAF4 women (n=1037)
LBC1921 men (n=152)
LBC1921 women (n=191)
LBC1936 men (n=403)
LBC1936 women (n=328)
LifeLines men (n=1672)
LifeLines women (n=2384)
LLS 02112015 (n=2132)
MICROS (n=1031)
NBS men (n=842)
NBS women (n=843)
PROSPER men (n=2172)
PROSPER women (n=2043)
RSI men (n=1286)
RSI women (n=1633)
RSII men (n=848)
RSII women (n=883)
RSIII men (n=1208)
RSIII women (n=1386)
SardiNIA (n=5552)
SHIP (n=369)
SHIP (n=421)
SHIP men (n=1741)
SHIP women (n=1592)
TwinsUK (n=3270)
meta analysis: I²=40.4 (n=64423)
−1
−0.5
0
0.5
TSH (rs1265091_T)

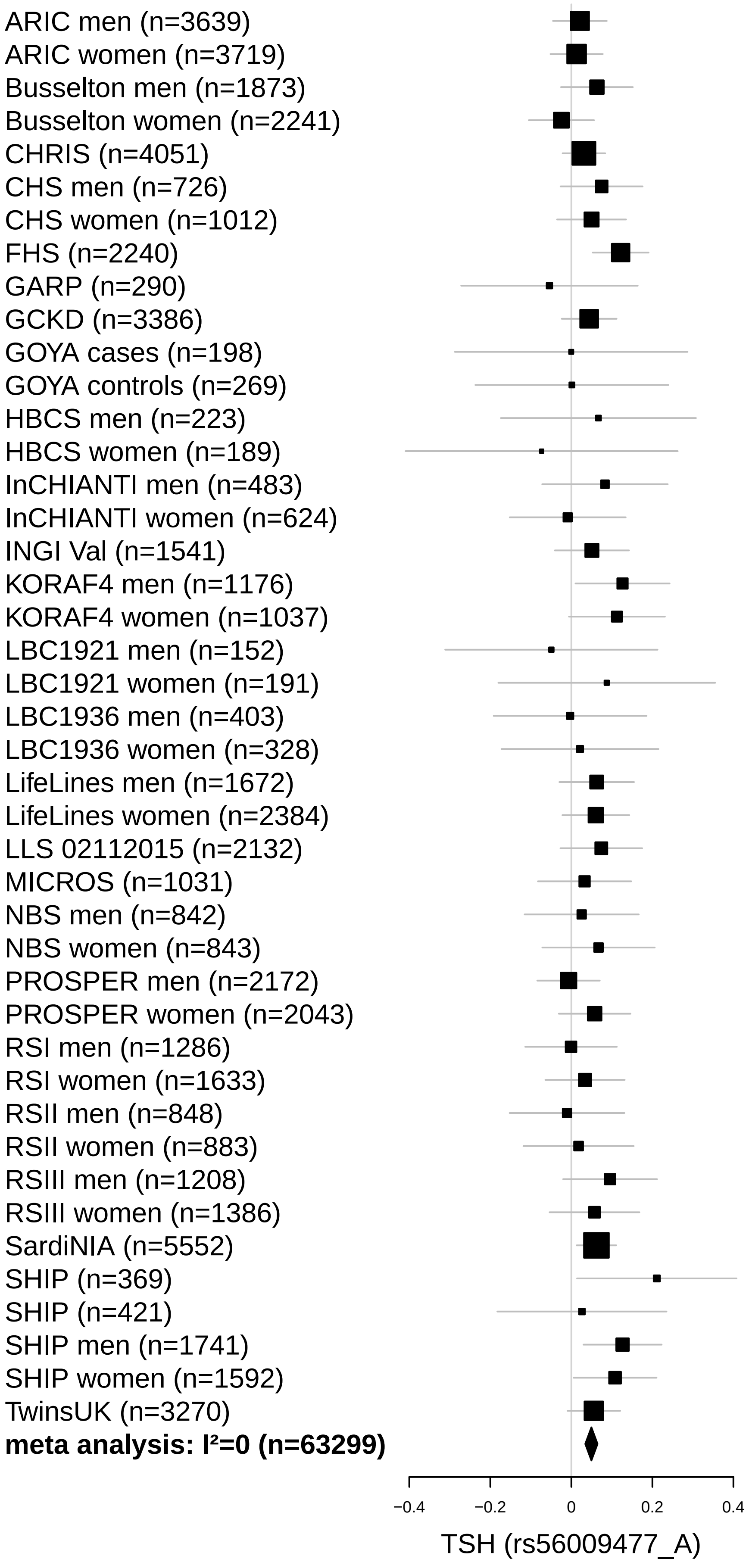
ARIC men (n=3639)
ARIC women (n=3719)
Busselton men (n=1873)
Busselton women (n=2241)
CHRIS (n=4051)
CHS men (n=726)
CHS women (n=1012)
FHS (n=2240)
GARP (n=290)
GCKD (n=3386)
GOYA cases (n=198)
GOYA controls (n=269)
HBCS men (n=223)
HBCS women (n=189)
InCHIANTI men (n=483)
InCHIANTI women (n=624)
INGI Val (n=1541)
KORAF4 men (n=1176)
KORAF4 women (n=1037)
LBC1921 men (n=152)
LBC1921 women (n=191)
LBC1936 men (n=403)
LBC1936 women (n=328)
LifeLines men (n=1672)
LifeLines women (n=2384)
LLS 02112015 (n=2132)
MICROS (n=1031)
NBS men (n=842)
NBS women (n=843)
PROSPER men (n=2172)
PROSPER women (n=2043)
RSI men (n=1286)
RSI women (n=1633)
RSII men (n=848)
RSII women (n=883)
RSIII men (n=1208)
RSIII women (n=1386)
SardiNIA (n=5552)
SHIP (n=369)
SHIP (n=421)
SHIP men (n=1741)
SHIP women (n=1592)
TwinsUK (n=3270)
meta analysis: I²=0 (n=63299)
−0.4
−0.2
0
0.2
0.4
TSH (rs56009477_A)

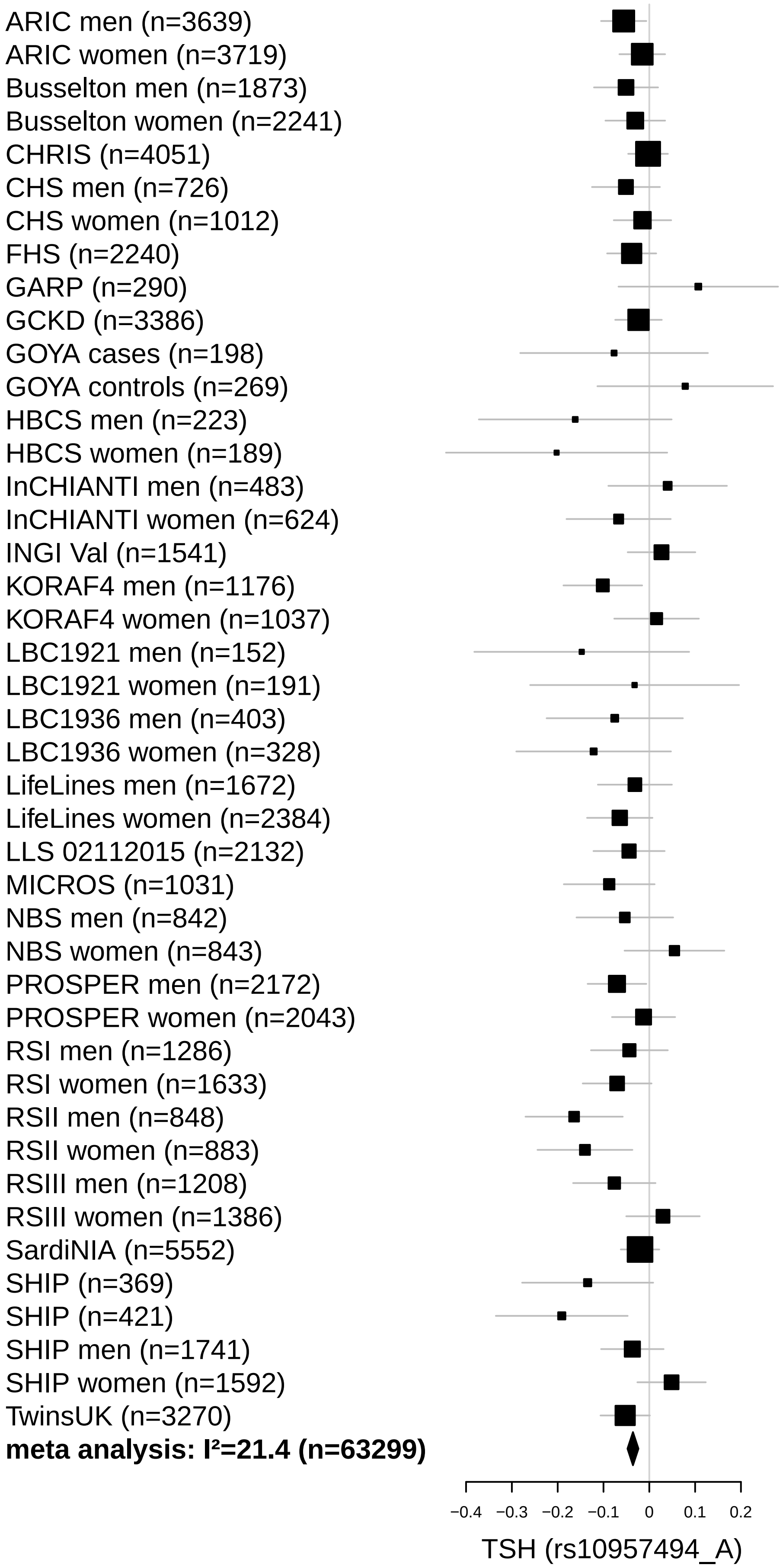
ARIC men (n=3639)
ARIC women (n=3719)
Busselton men (n=1873)
Busselton women (n=2241)
CHRIS (n=4051)
CHS men (n=726)
CHS women (n=1012)
FHS (n=2240)
GARP (n=290)
GCKD (n=3386)
GOYA cases (n=198)
GOYA controls (n=269)
HBCS men (n=223)
HBCS women (n=189)
InCHIANTI men (n=483)
InCHIANTI women (n=624)
INGI Val (n=1541)
KORAF4 men (n=1176)
KORAF4 women (n=1037)
LBC1921 men (n=152)
LBC1921 women (n=191)
LBC1936 men (n=403)
LBC1936 women (n=328)
LifeLines men (n=1672)
LifeLines women (n=2384)
LLS 02112015 (n=2132)
MICROS (n=1031)
NBS men (n=842)
NBS women (n=843)
PROSPER men (n=2172)
PROSPER women (n=2043)
RSI men (n=1286)
RSI women (n=1633)
RSII men (n=848)
RSII women (n=883)
RSIII men (n=1208)
RSIII women (n=1386)
SardiNIA (n=5552)
SHIP (n=369)
SHIP (n=421)
SHIP men (n=1741)
SHIP women (n=1592)
TwinsUK (n=3270)
meta analysis: I²=21.4 (n=63299)
−0.4
−0.3
−0.2
−0.1
0
0.1
0.2
TSH (rs10957494_A)

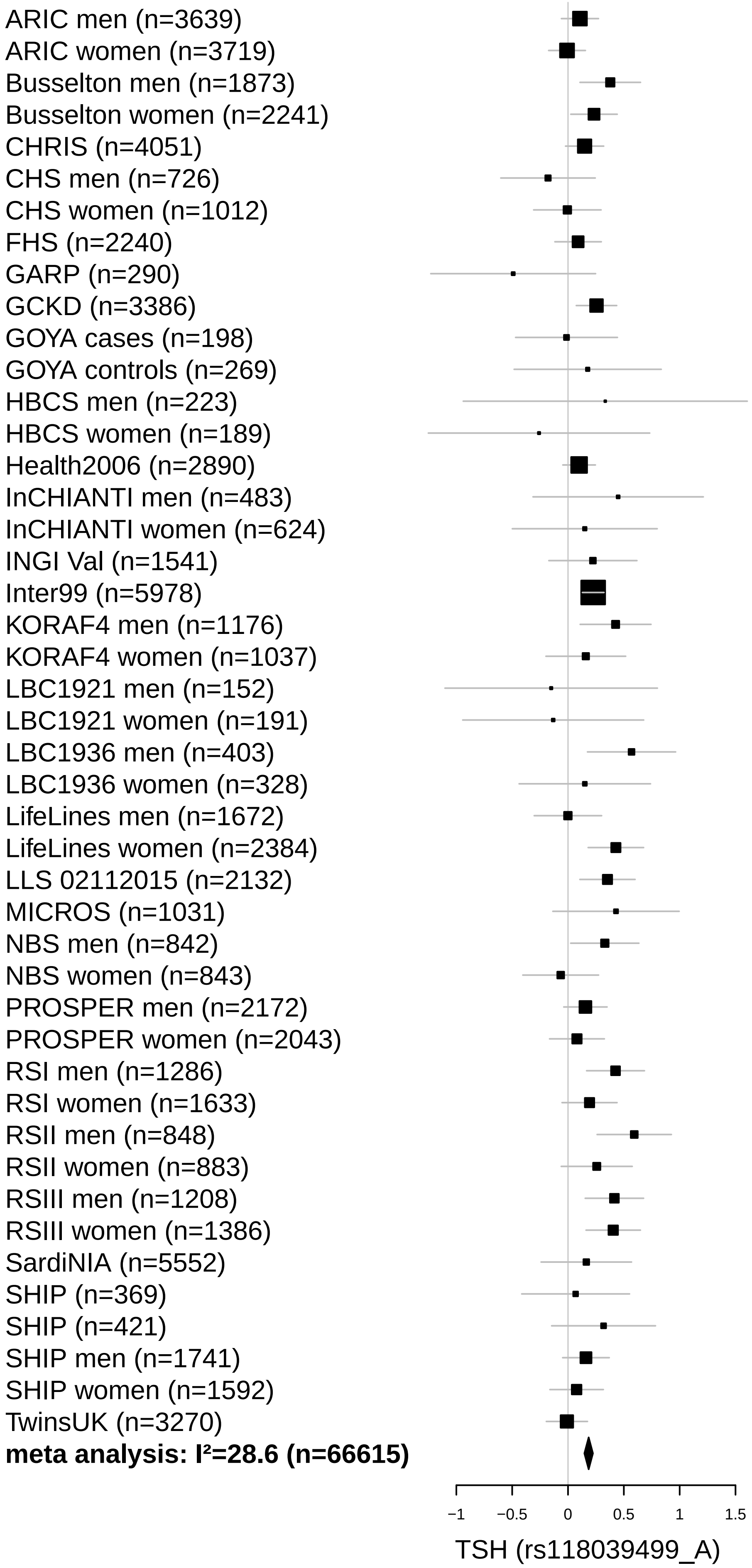
ARIC men (n=3639)
ARIC women (n=3719)
Busselton men (n=1873)
Busselton women (n=2241)
CHRIS (n=4051)
CHS men (n=726)
CHS women (n=1012)
FHS (n=2240)
GARP (n=290)
GCKD (n=3386)
GOYA cases (n=198)
GOYA controls (n=269)
HBCS men (n=223)
HBCS women (n=189)
Health2006 (n=2890)
InCHIANTI men (n=483)
InCHIANTI women (n=624)
INGI Val (n=1541)
Inter99 (n=5978)
KORAF4 men (n=1176)
KORAF4 women (n=1037)
LBC1921 men (n=152)
LBC1921 women (n=191)
LBC1936 men (n=403)
LBC1936 women (n=328)
LifeLines men (n=1672)
LifeLines women (n=2384)
LLS 02112015 (n=2132)
MICROS (n=1031)
NBS men (n=842)
NBS women (n=843)
PROSPER men (n=2172)
PROSPER women (n=2043)
RSI men (n=1286)
RSI women (n=1633)
RSII men (n=848)
RSII women (n=883)
RSIII men (n=1208)
RSIII women (n=1386)
SardiNIA (n=5552)
SHIP (n=369)
SHIP (n=421)
SHIP men (n=1741)
SHIP women (n=1592)
TwinsUK (n=3270)
meta analysis: I²=28.6 (n=66615)
−1
−0.5
0
0.5
1
1.5
TSH (rs118039499_A)

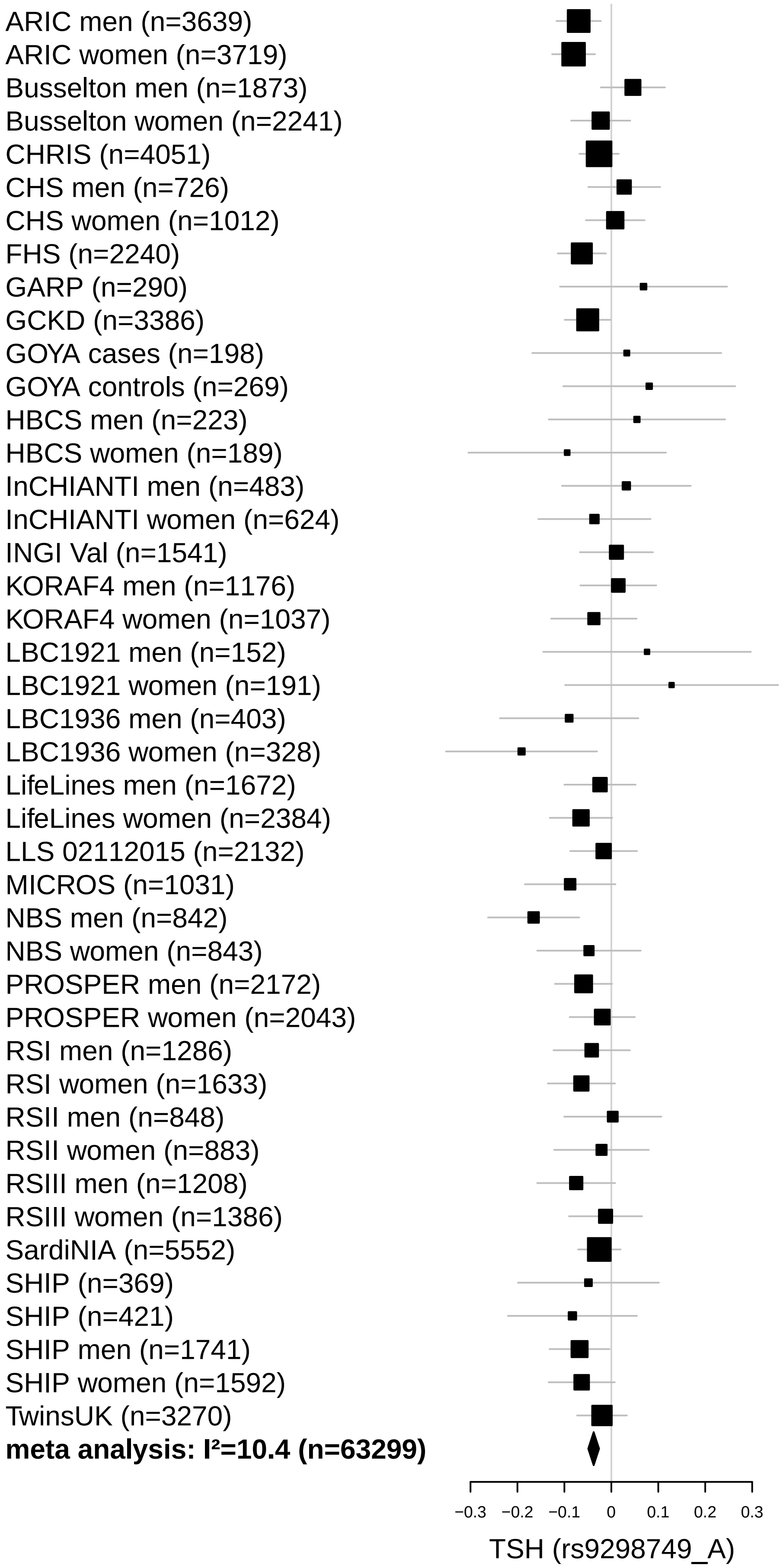
ARIC men (n=3639)
ARIC women (n=3719)
Busselton men (n=1873)
Busselton women (n=2241)
CHRIS (n=4051)
CHS men (n=726)
CHS women (n=1012)
FHS (n=2240)
GARP (n=290)
GCKD (n=3386)
GOYA cases (n=198)
GOYA controls (n=269)
HBCS men (n=223)
HBCS women (n=189)
InCHIANTI men (n=483)
InCHIANTI women (n=624)
INGI Val (n=1541)
KORAF4 men (n=1176)
KORAF4 women (n=1037)
LBC1921 men (n=152)
LBC1921 women (n=191)
LBC1936 men (n=403)
LBC1936 women (n=328)
LifeLines men (n=1672)
LifeLines women (n=2384)
LLS 02112015 (n=2132)
MICROS (n=1031)
NBS men (n=842)
NBS women (n=843)
PROSPER men (n=2172)
PROSPER women (n=2043)
RSI men (n=1286)
RSI women (n=1633)
RSII men (n=848)
RSII women (n=883)
RSIII men (n=1208)
RSIII women (n=1386)
SardiNIA (n=5552)
SHIP (n=369)
SHIP (n=421)
SHIP men (n=1741)
SHIP women (n=1592)
TwinsUK (n=3270)
meta analysis: I²=10.4 (n=63299)
−0.3
−0.2
−0.1
0
0.1
0.2
0.3
TSH (rs9298749_A)

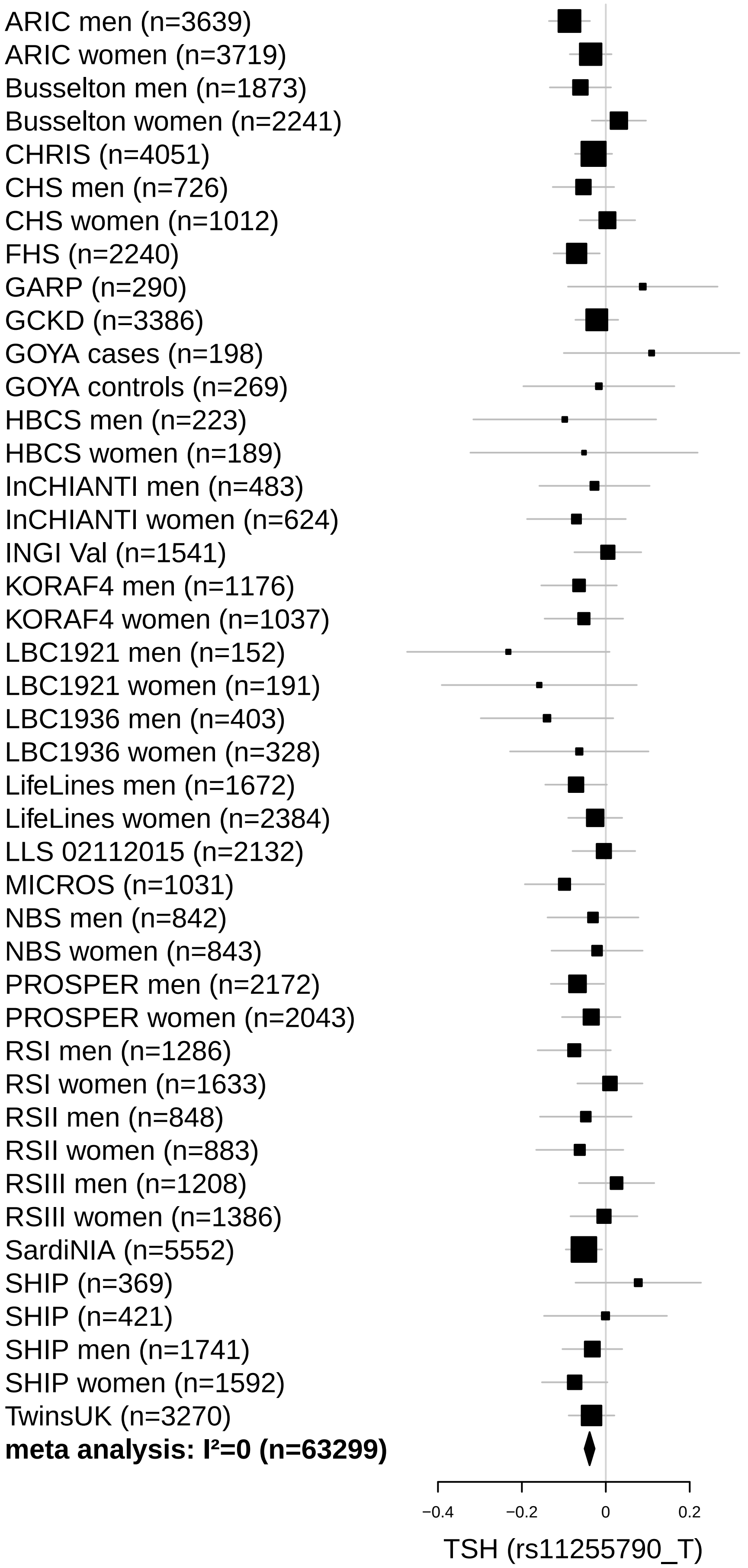
ARIC men (n=3639)
ARIC women (n=3719)
Busselton men (n=1873)
Busselton women (n=2241)
CHRIS (n=4051)
CHS men (n=726)
CHS women (n=1012)
FHS (n=2240)
GARP (n=290)
GCKD (n=3386)
GOYA cases (n=198)
GOYA controls (n=269)
HBCS men (n=223)
HBCS women (n=189)
InCHIANTI men (n=483)
InCHIANTI women (n=624)
INGI Val (n=1541)
KORAF4 men (n=1176)
KORAF4 women (n=1037)
LBC1921 men (n=152)
LBC1921 women (n=191)
LBC1936 men (n=403)
LBC1936 women (n=328)
LifeLines men (n=1672)
LifeLines women (n=2384)
LLS 02112015 (n=2132)
MICROS (n=1031)
NBS men (n=842)
NBS women (n=843)
PROSPER men (n=2172)
PROSPER women (n=2043)
RSI men (n=1286)
RSI women (n=1633)
RSII men (n=848)
RSII women (n=883)
RSIII men (n=1208)
RSIII women (n=1386)
SardiNIA (n=5552)
SHIP (n=369)
SHIP (n=421)
SHIP men (n=1741)
SHIP women (n=1592)
TwinsUK (n=3270)
meta analysis: I²=0 (n=63299)
−0.4
−0.2
0
0.2
TSH (rs11255790_T)

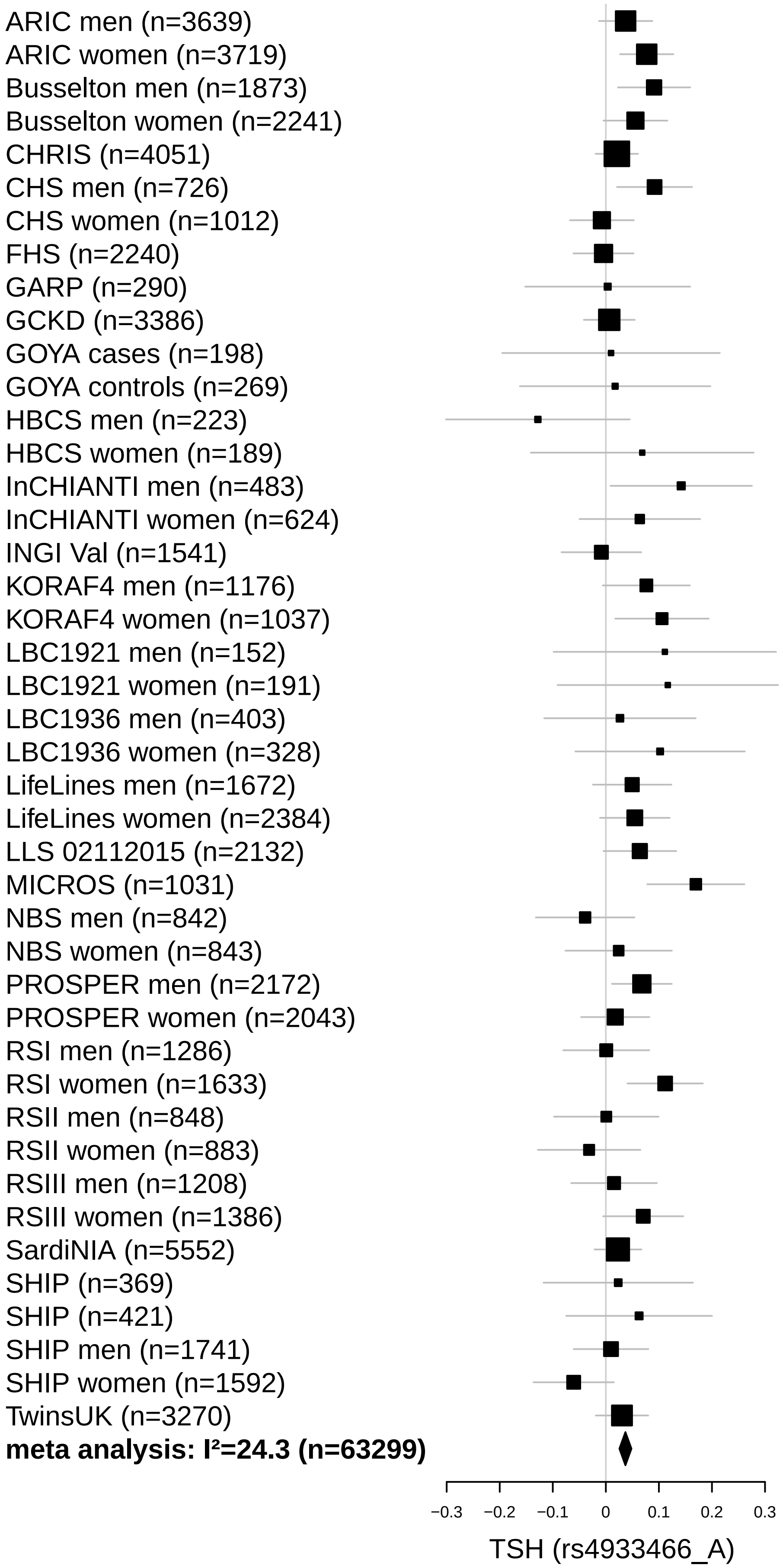
ARIC men (n=3639)
ARIC women (n=3719)
Busselton men (n=1873)
Busselton women (n=2241)
CHRIS (n=4051)
CHS men (n=726)
CHS women (n=1012)
FHS (n=2240)
GARP (n=290)
GCKD (n=3386)
GOYA cases (n=198)
GOYA controls (n=269)
HBCS men (n=223)
HBCS women (n=189)
InCHIANTI men (n=483)
InCHIANTI women (n=624)
INGI Val (n=1541)
KORAF4 men (n=1176)
KORAF4 women (n=1037)
LBC1921 men (n=152)
LBC1921 women (n=191)
LBC1936 men (n=403)
LBC1936 women (n=328)
LifeLines men (n=1672)
LifeLines women (n=2384)
LLS 02112015 (n=2132)
MICROS (n=1031)
NBS men (n=842)
NBS women (n=843)
PROSPER men (n=2172)
PROSPER women (n=2043)
RSI men (n=1286)
RSI women (n=1633)
RSII men (n=848)
RSII women (n=883)
RSIII men (n=1208)
RSIII women (n=1386)
SardiNIA (n=5552)
SHIP (n=369)
SHIP (n=421)
SHIP men (n=1741)
SHIP women (n=1592)
TwinsUK (n=3270)
meta analysis: I²=24.3 (n=63299)
−0.3
−0.2
−0.1
0
0.1
0.2
0.3
TSH (rs4933466_A)

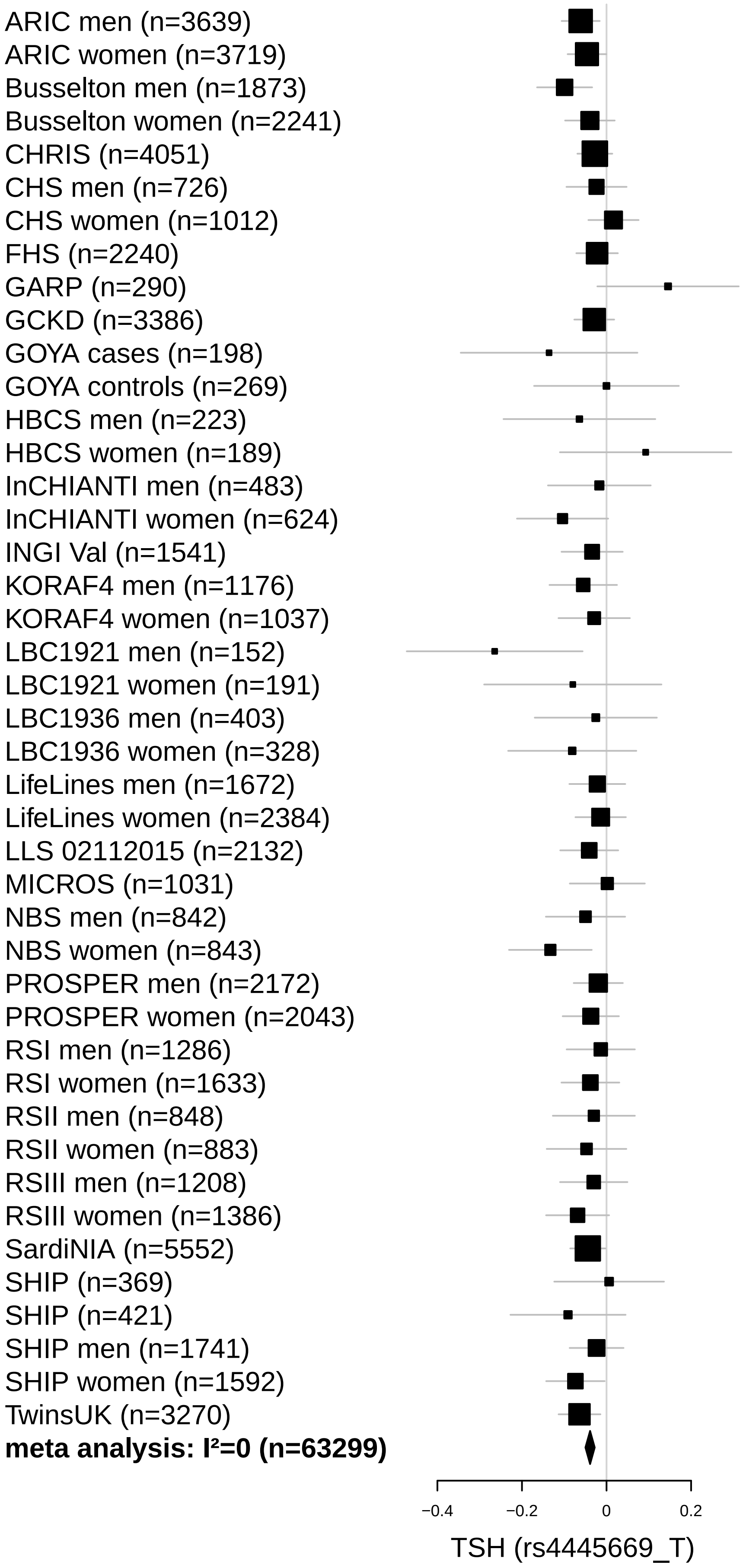
ARIC men (n=3639)
ARIC women (n=3719)
Busselton men (n=1873)
Busselton women (n=2241)
CHRIS (n=4051)
CHS men (n=726)
CHS women (n=1012)
FHS (n=2240)
GARP (n=290)
GCKD (n=3386)
GOYA cases (n=198)
GOYA controls (n=269)
HBCS men (n=223)
HBCS women (n=189)
InCHIANTI men (n=483)
InCHIANTI women (n=624)
INGI Val (n=1541)
KORAF4 men (n=1176)
KORAF4 women (n=1037)
LBC1921 men (n=152)
LBC1921 women (n=191)
LBC1936 men (n=403)
LBC1936 women (n=328)
LifeLines men (n=1672)
LifeLines women (n=2384)
LLS 02112015 (n=2132)
MICROS (n=1031)
NBS men (n=842)
NBS women (n=843)
PROSPER men (n=2172)
PROSPER women (n=2043)
RSI men (n=1286)
RSI women (n=1633)
RSII men (n=848)
RSII women (n=883)
RSIII men (n=1208)
RSIII women (n=1386)
SardiNIA (n=5552)
SHIP (n=369)
SHIP (n=421)
SHIP men (n=1741)
SHIP women (n=1592)
TwinsUK (n=3270)
meta analysis: I²=0 (n=63299)
−0.4
−0.2
0
0.2
TSH (rs4445669_T)

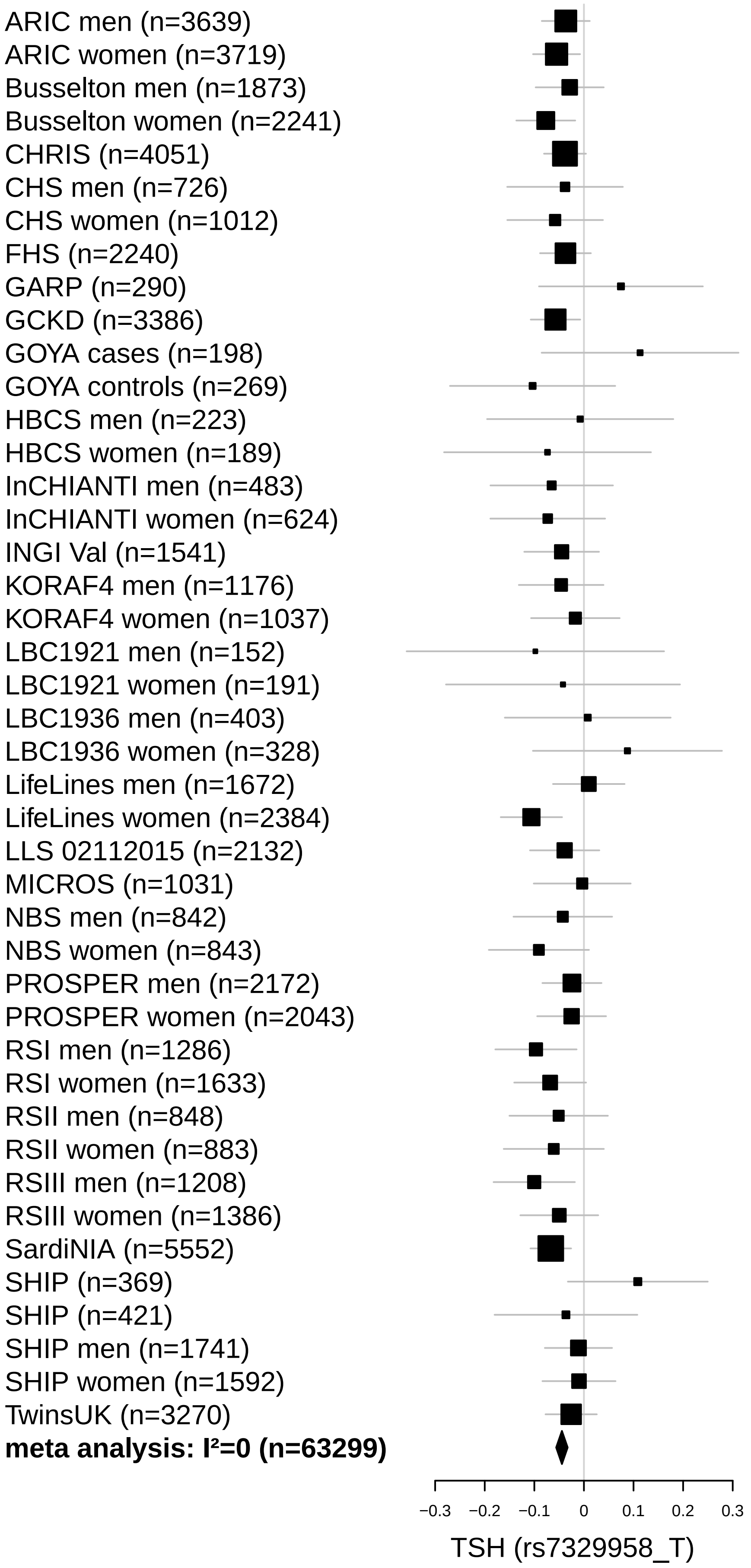

ARIC men (n=3639)
ARIC women (n=3719)
Busselton men (n=1873)
Busselton women (n=2241)
CHRIS (n=4051)
CHS men (n=726)
CHS women (n=1012)
FHS (n=2240)
GARP (n=290)
GCKD (n=3386)
GOYA cases (n=198)
GOYA controls (n=269)
HBCS men (n=223)
HBCS women (n=189)
InCHIANTI men (n=483)
InCHIANTI women (n=624)
INGI Val (n=1541)
KORAF4 men (n=1176)
KORAF4 women (n=1037)
LBC1921 men (n=152)
LBC1921 women (n=191)
LBC1936 men (n=403)
LBC1936 women (n=328)
LifeLines men (n=1672)
LifeLines women (n=2384)
LLS 02112015 (n=2132)
MICROS (n=1031)
NBS men (n=842)
NBS women (n=843)
PROSPER men (n=2172)
PROSPER women (n=2043)
RSI men (n=1286)
RSI women (n=1633)
RSII men (n=848)
RSII women (n=883)
RSIII men (n=1208)
RSIII women (n=1386)
SardiNIA (n=5552)
SHIP (n=369)
SHIP (n=421)
SHIP men (n=1741)
SHIP women (n=1592)
TwinsUK (n=3270)
meta analysis: I²=0 (n=63299)
−0.3 −0.2 −0.1 0 0.1 0.2 0.3
TSH (rs7329958_T)

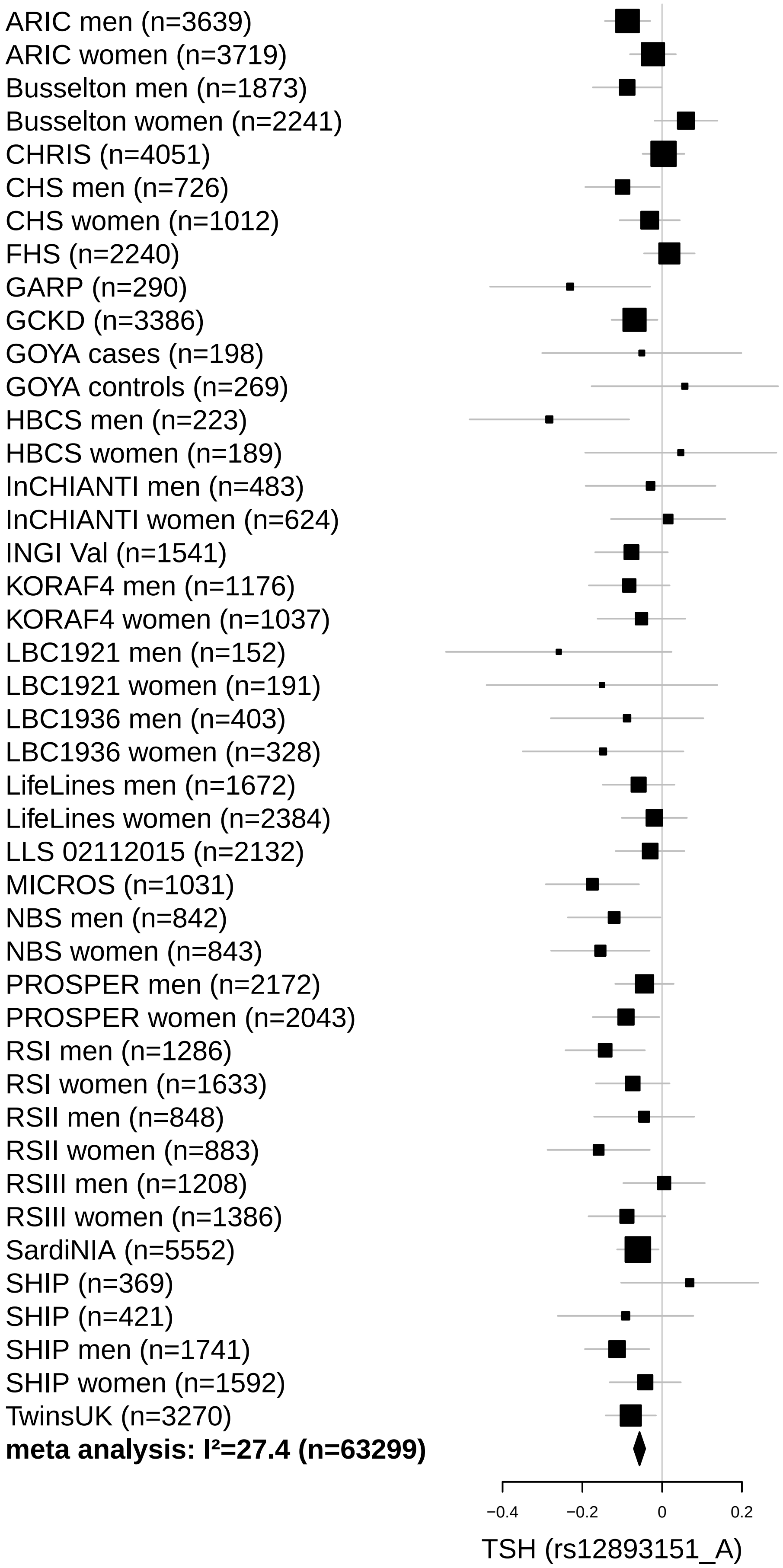
ARIC men (n=3639)
ARIC women (n=3719)
Busselton men (n=1873)
Busselton women (n=2241)
CHRIS (n=4051)
CHS men (n=726)
CHS women (n=1012)
FHS (n=2240)
GARP (n=290)
GCKD (n=3386)
GOYA cases (n=198)
GOYA controls (n=269)
HBCS men (n=223)
HBCS women (n=189)
InCHIANTI men (n=483)
InCHIANTI women (n=624)
INGI Val (n=1541)
KORAF4 men (n=1176)
KORAF4 women (n=1037)
LBC1921 men (n=152)
LBC1921 women (n=191)
LBC1936 men (n=403)
LBC1936 women (n=328)
LifeLines men (n=1672)
LifeLines women (n=2384)
LLS 02112015 (n=2132)
MICROS (n=1031)
NBS men (n=842)
NBS women (n=843)
PROSPER men (n=2172)
PROSPER women (n=2043)
RSI men (n=1286)
RSI women (n=1633)
RSII men (n=848)
RSII women (n=883)
RSIII men (n=1208)
RSIII women (n=1386)
SardiNIA (n=5552)
SHIP (n=369)
SHIP (n=421)
SHIP men (n=1741)
SHIP women (n=1592)
TwinsUK (n=3270)
meta analysis: I²=27.4 (n=63299)
−0.4
−0.2
0
0.2
TSH (rs12893151_A)

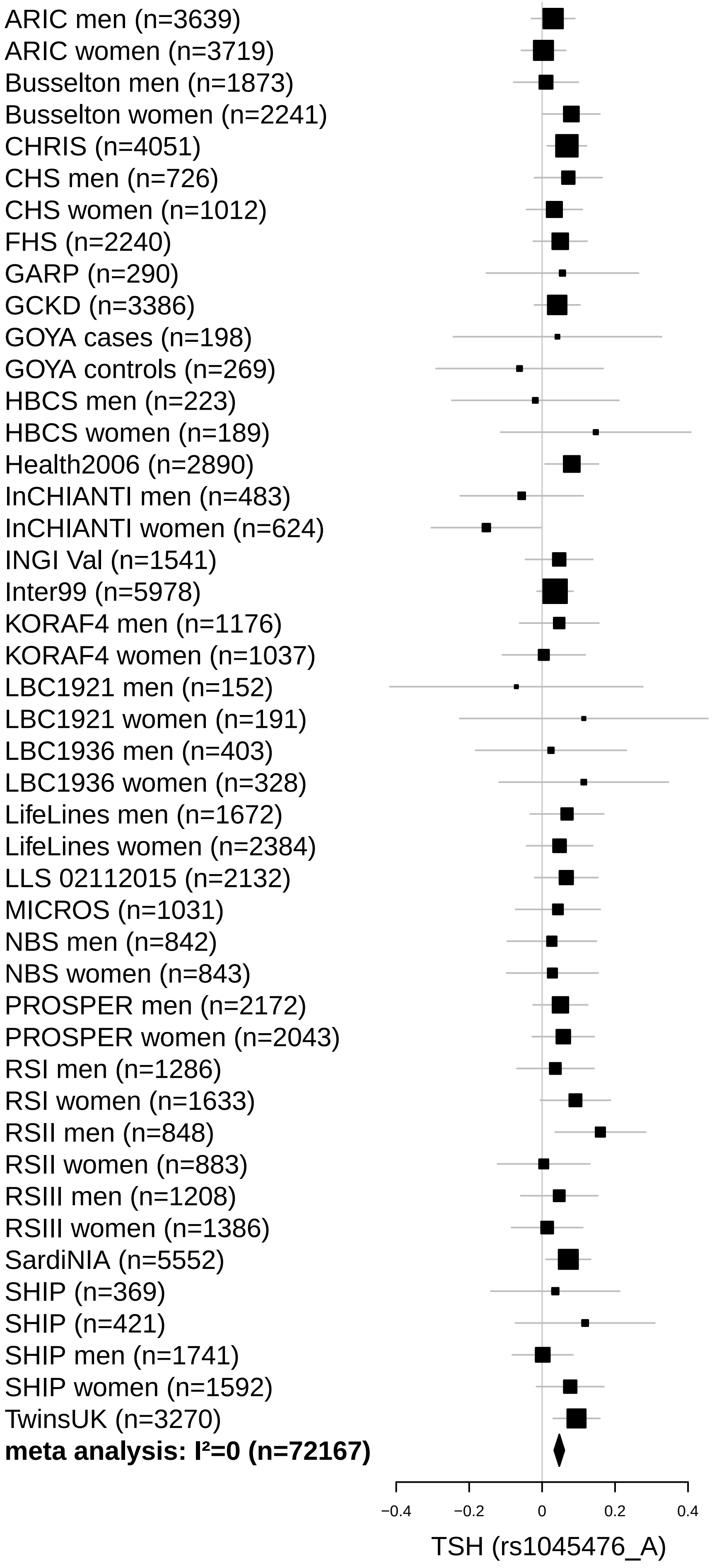
ARIC men (n=3639)
ARIC women (n=3719)
Busselton men (n=1873)
Busselton women (n=2241)
CHRIS (n=4051)
CHS men (n=726)
CHS women (n=1012)
FHS (n=2240)
GARP (n=290)
GCKD (n=3386)
GOYA cases (n=198)
GOYA controls (n=269)
HBCS men (n=223)
HBCS women (n=189)
Health2006 (n=2890)
InCHIANTI men (n=483)
InCHIANTI women (n=624)
INGI Val (n=1541)
Inter99 (n=5978)
KORAF4 men (n=1176)
KORAF4 women (n=1037)
LBC1921 men (n=152)
LBC1921 women (n=191)
LBC1936 men (n=403)
LBC1936 women (n=328)
LifeLines men (n=1672)
LifeLines women (n=2384)
LLS 02112015 (n=2132)
MICROS (n=1031)
NBS men (n=842)
NBS women (n=843)
PROSPER men (n=2172)
PROSPER women (n=2043)
RSI men (n=1286)
RSI women (n=1633)
RSII men (n=848)
RSII women (n=883)
RSIII men (n=1208)
RSIII women (n=1386)
SardiNIA (n=5552)
SHIP (n=369)
SHIP (n=421)
SHIP men (n=1741)
SHIP women (n=1592)
TwinsUK (n=3270)
meta analysis: I²=0 (n=72167)
−0.4
−0.2
0
0.2
0.4
TSH (rs1045476_A)

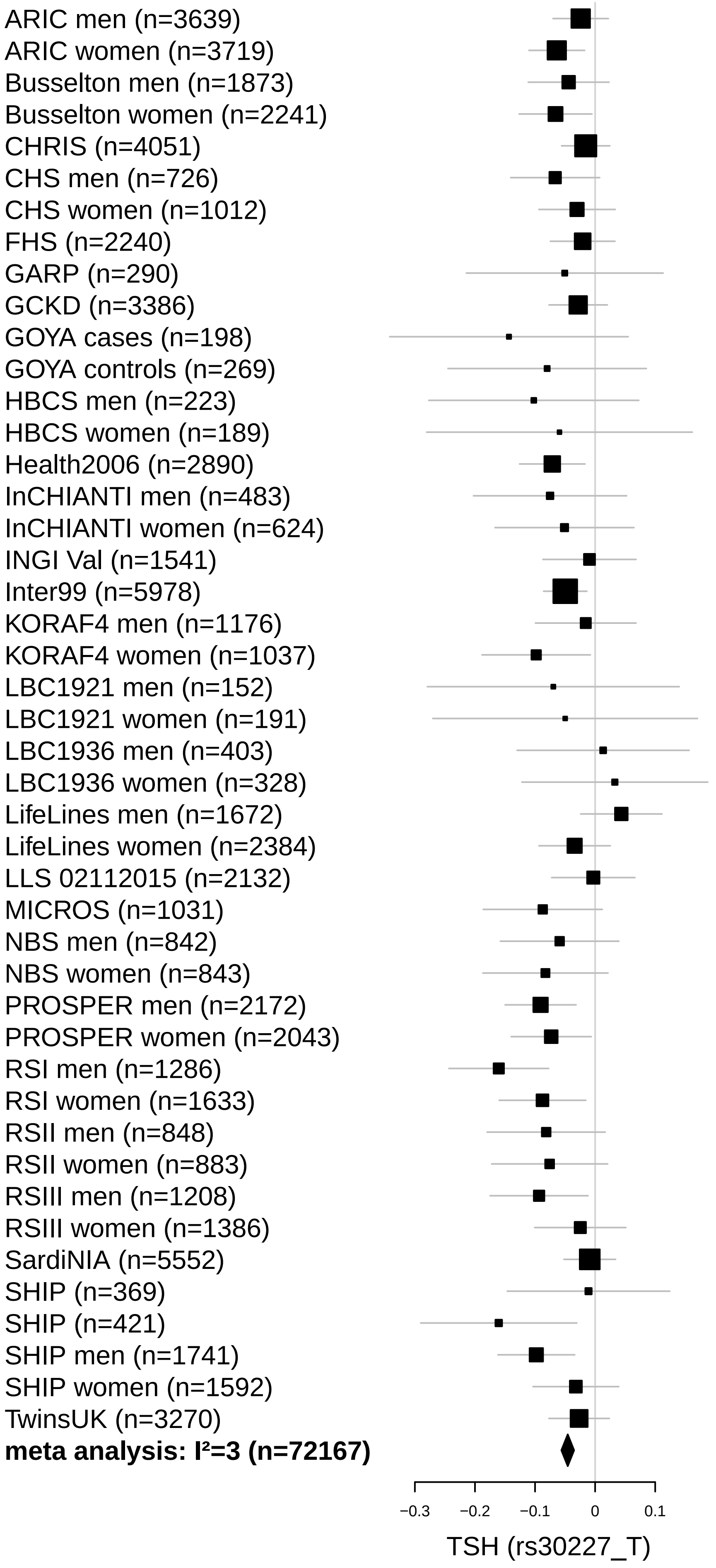

ARIC men (n=3639)
ARIC women (n=3719)
Busselton men (n=1873)
Busselton women (n=2241)
CHRIS (n=4051)
CHS men (n=726)
CHS women (n=1012)
FHS (n=2240)
GARP (n=290)
GCKD (n=3386)
GOYA cases (n=198)
GOYA controls (n=269)
HBCS men (n=223)
HBCS women (n=189)
Health2006 (n=2890)
InCHIANTI men (n=483)
InCHIANTI women (n=624)
INGI Val (n=1541)
Inter99 (n=5978)
KORAF4 men (n=1176)
KORAF4 women (n=1037)
LBC1921 men (n=152)
LBC1921 women (n=191)
LBC1936 men (n=403)
LBC1936 women (n=328)
LifeLines men (n=1672)
LifeLines women (n=2384)
LLS 02112015 (n=2132)
MICROS (n=1031)
NBS men (n=842)
NBS women (n=843)
PROSPER men (n=2172)
PROSPER women (n=2043)
RSI men (n=1286)
RSI women (n=1633)
RSII men (n=848)
RSII women (n=883)
RSIII men (n=1208)
RSIII women (n=1386)
SardiNIA (n=5552)
SHIP (n=369)
SHIP (n=421)
SHIP men (n=1741)
SHIP women (n=1592)
TwinsUK (n=3270)
meta analysis: I²=3 (n=72167)
−0.3
−0.2
−0.1
0
0.1
TSH (rs30227_T)

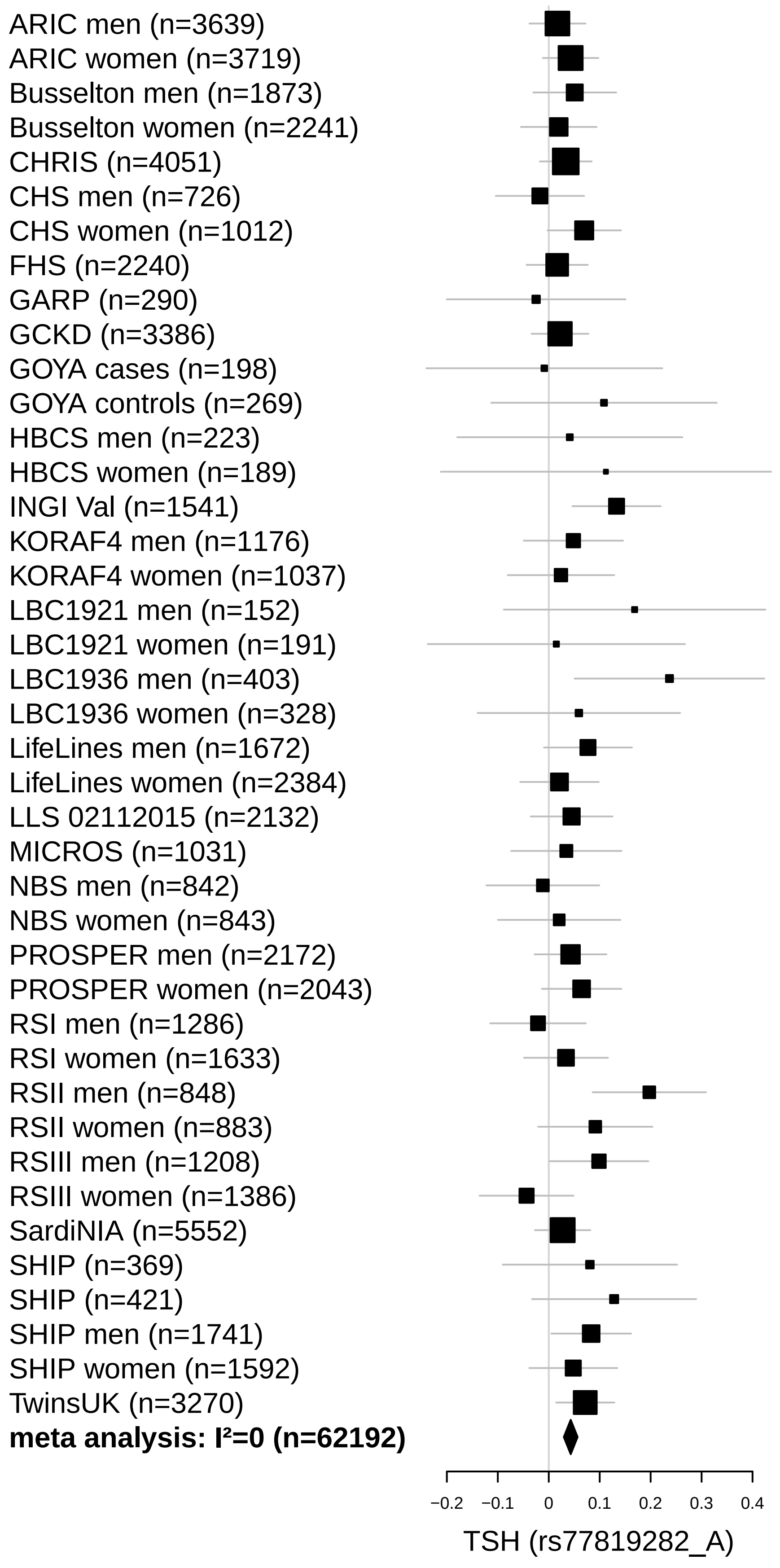

ARIC men (n=3639)
ARIC women (n=3719)
Busselton men (n=1873)
Busselton women (n=2241)
CHRIS (n=4051)
CHS men (n=726)
CHS women (n=1012)
FHS (n=2240)
GARP (n=290)
GCKD (n=3386)
GOYA cases (n=198)
GOYA controls (n=269)
HBCS men (n=223)
HBCS women (n=189)
INGI Val (n=1541)
KORAF4 men (n=1176)
KORAF4 women (n=1037)
LBC1921 men (n=152)
LBC1921 women (n=191)
LBC1936 men (n=403)
LBC1936 women (n=328)
LifeLines men (n=1672)
LifeLines women (n=2384)
LLS 02112015 (n=2132)
MICROS (n=1031)
NBS men (n=842)
NBS women (n=843)
PROSPER men (n=2172)
PROSPER women (n=2043)
RSI men (n=1286)
RSI women (n=1633)
RSII men (n=848)
RSII women (n=883)
RSIII men (n=1208)
RSIII women (n=1386)
SardiNIA (n=5552)
SHIP (n=369)
SHIP (n=421)
SHIP men (n=1741)
SHIP women (n=1592)
TwinsUK (n=3270)
meta analysis: I²=0 (n=62192)
−0.2
−0.1
0
0.1
0.2
0.3
0.4
TSH (rs77819282_A)

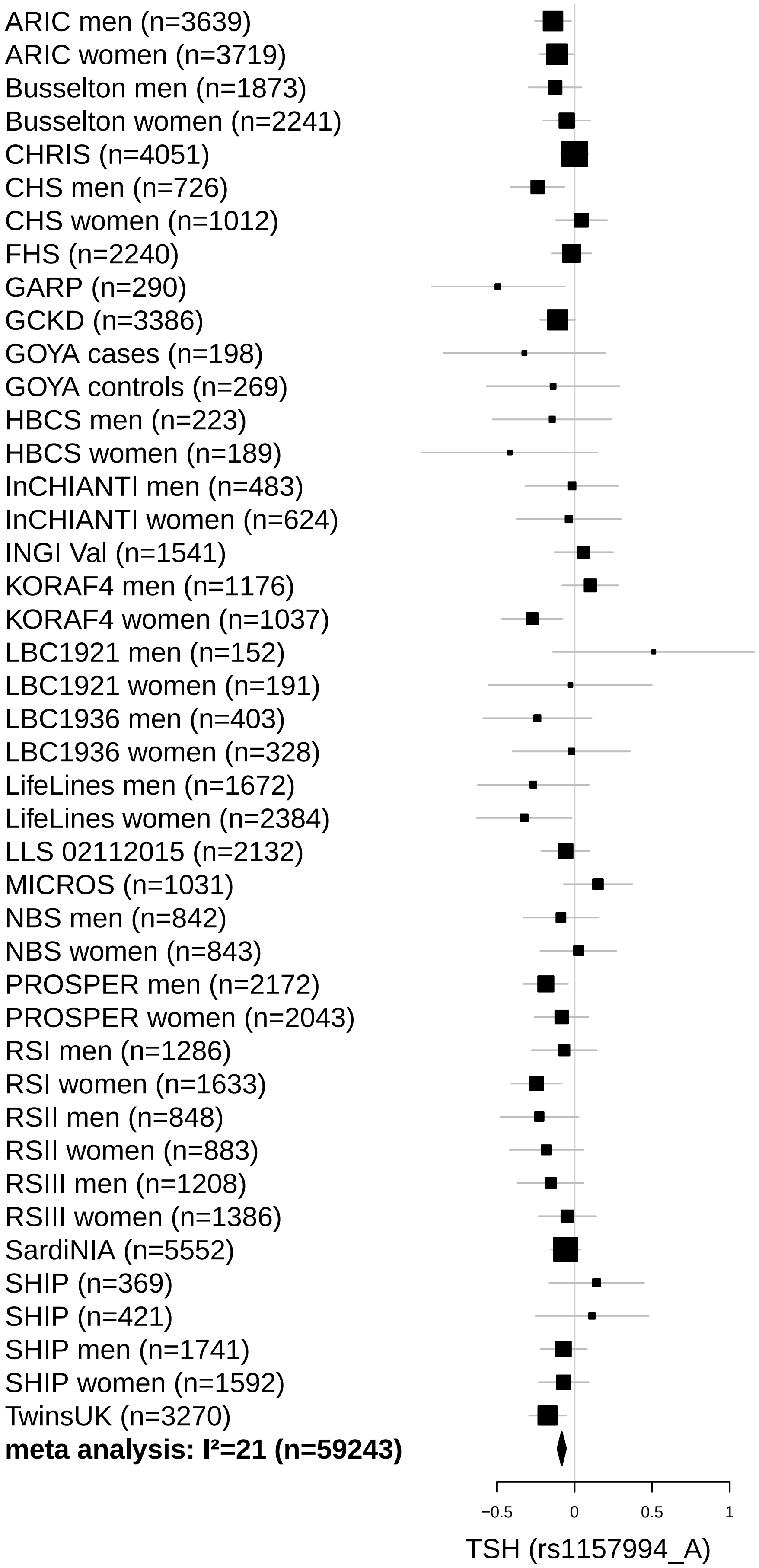
ARIC men (n=3639)
ARIC women (n=3719)
Busselton men (n=1873)
Busselton women (n=2241)
CHRIS (n=4051)
CHS men (n=726)
CHS women (n=1012)
FHS (n=2240)
GARP (n=290)
GCKD (n=3386)
GOYA cases (n=198)
GOYA controls (n=269)
HBCS men (n=223)
HBCS women (n=189)
InCHIANTI men (n=483)
InCHIANTI women (n=624)
INGI Val (n=1541)
KORAF4 men (n=1176)
KORAF4 women (n=1037)
LBC1921 men (n=152)
LBC1921 women (n=191)
LBC1936 men (n=403)
LBC1936 women (n=328)
LifeLines men (n=1672)
LifeLines women (n=2384)
LLS 02112015 (n=2132)
MICROS (n=1031)
NBS men (n=842)
NBS women (n=843)
PROSPER men (n=2172)
PROSPER women (n=2043)
RSI men (n=1286)
RSI women (n=1633)
RSII men (n=848)
RSII women (n=883)
RSIII men (n=1208)
RSIII women (n=1386)
SardiNIA (n=5552)
SHIP (n=369)
SHIP (n=421)
SHIP men (n=1741)
SHIP women (n=1592)
TwinsUK (n=3270)
meta analysis: I²=21 (n=59243)
−0.5
0
0.5
1
TSH (rs1157994_A)

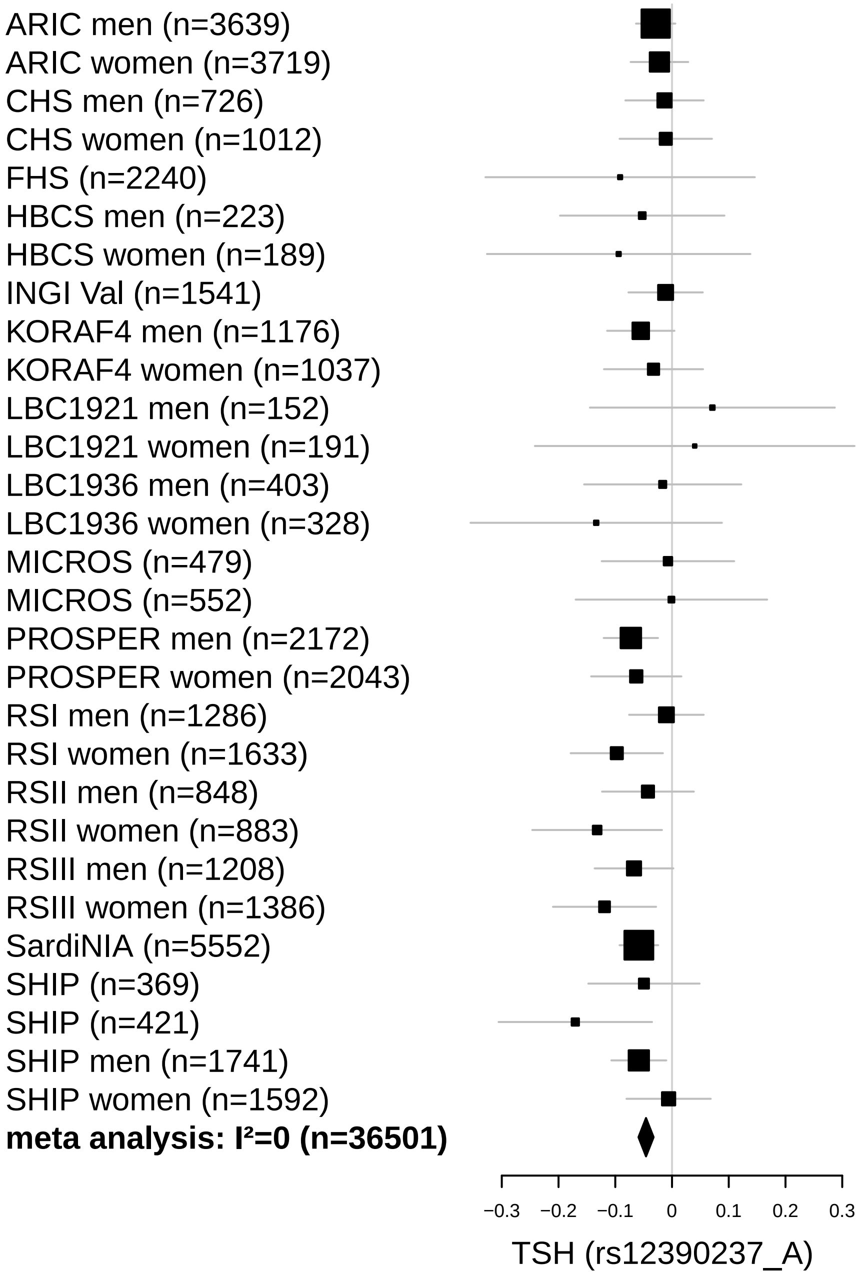
ARIC men (n=3639)
ARIC women (n=3719)
CHS men (n=726)
CHS women (n=1012)
FHS (n=2240)
HBCS men (n=223)
HBCS women (n=189)
INGI Val (n=1541)
KORAF4 men (n=1176)
KORAF4 women (n=1037)
LBC1921 men (n=152)
LBC1921 women (n=191)
LBC1936 men (n=403)
LBC1936 women (n=328)
MICROS (n=479)
MICROS (n=552)
PROSPER men (n=2172)
PROSPER women (n=2043)
RSI men (n=1286)
RSI women (n=1633)
RSII men (n=848)
RSII women (n=883)
RSIII men (n=1208)
RSIII women (n=1386)
SardiNIA (n=5552)
SHIP (n=369)
SHIP (n=421)
SHIP men (n=1741)
SHIP women (n=1592)
meta analysis: I²=0 (n=36501)
−0.3
−0.2
−0.1
0
0.1
0.2
0.3
TSH (rs12390237_A)

Forest plots of the 19 index SNPs of replicated susceptibility loci for TSH in the combined discovery and replication samples. Effects and their 95% confidence intervals are based on inverse normalized TSH values. I^2 is the percentage of total variation across studies that is due to heterogeneity.

Supplementary Figure 5: Forest plots for FT4

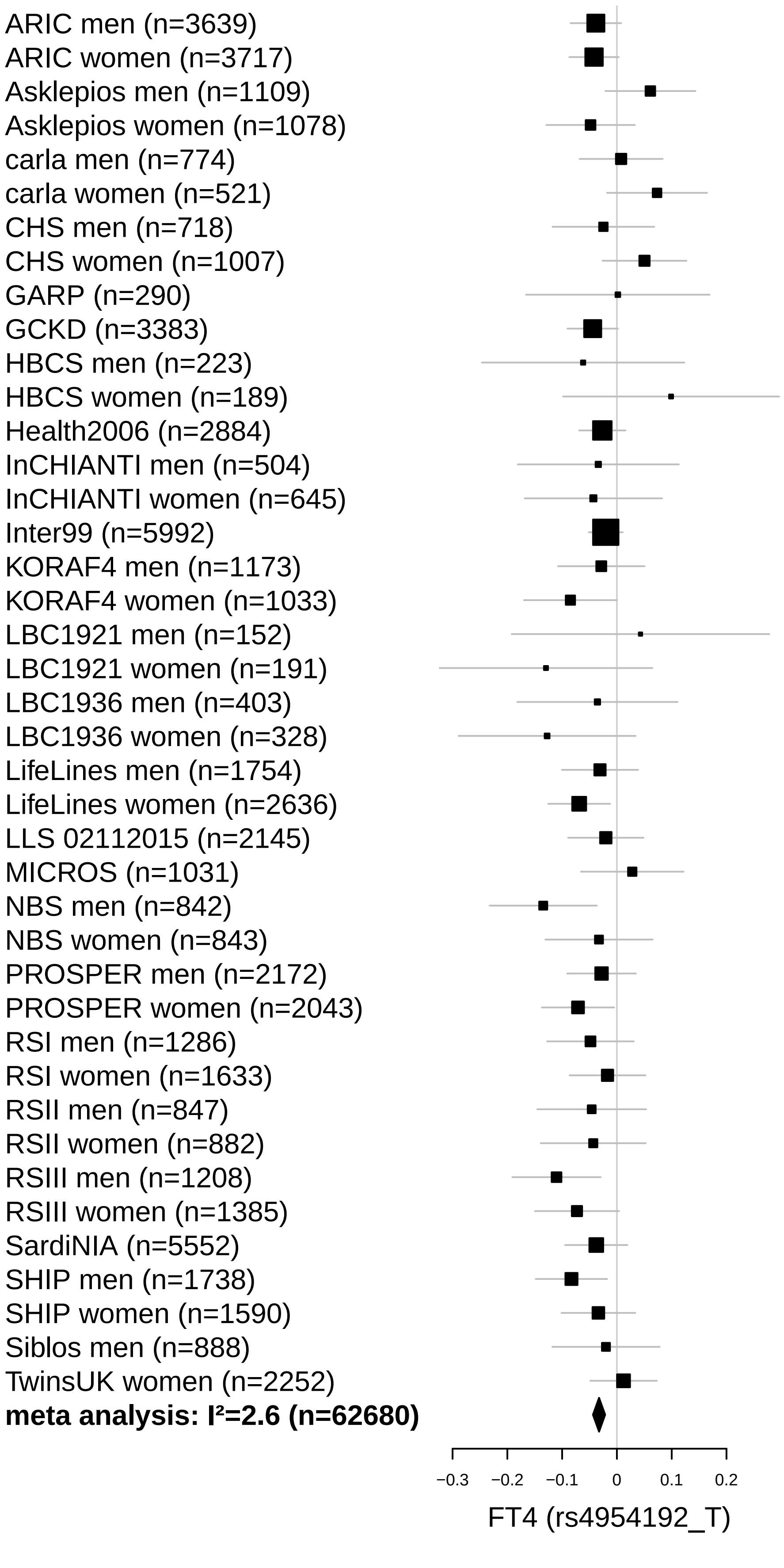
ARIC men (n=3639)
ARIC women (n=3717)
Asklepios men (n=1109)
Asklepios women (n=1078)
carla men (n=774)
carla women (n=521)
CHS men (n=718)
CHS women (n=1007)
GARP (n=290)
GCKD (n=3383)
HBCS men (n=223)
HBCS women (n=189)
Health2006 (n=2884)
InCHIANTI men (n=504)
InCHIANTI women (n=645)
Inter99 (n=5992)
KORAF4 men (n=1173)
KORAF4 women (n=1033)
LBC1921 men (n=152)
LBC1921 women (n=191)
LBC1936 men (n=403)
LBC1936 women (n=328)
LifeLines men (n=1754)
LifeLines women (n=2636)
LLS 02112015 (n=2145)
MICROS (n=1031)
NBS men (n=842)
NBS women (n=843)
PROSPER men (n=2172)
PROSPER women (n=2043)
RSI men (n=1286)
RSI women (n=1633)
RSII men (n=847)
RSII women (n=882)
RSIII men (n=1208)
RSIII women (n=1385)
SardiNIA (n=5552)
SHIP men (n=1738)
SHIP women (n=1590)
Siblos men (n=888)
TwinsUK women (n=2252)
meta analysis: I²=2.6 (n=62680)
−0.3
−0.2
−0.1
0
0.1
0.2
FT4 (rs4954192_T)

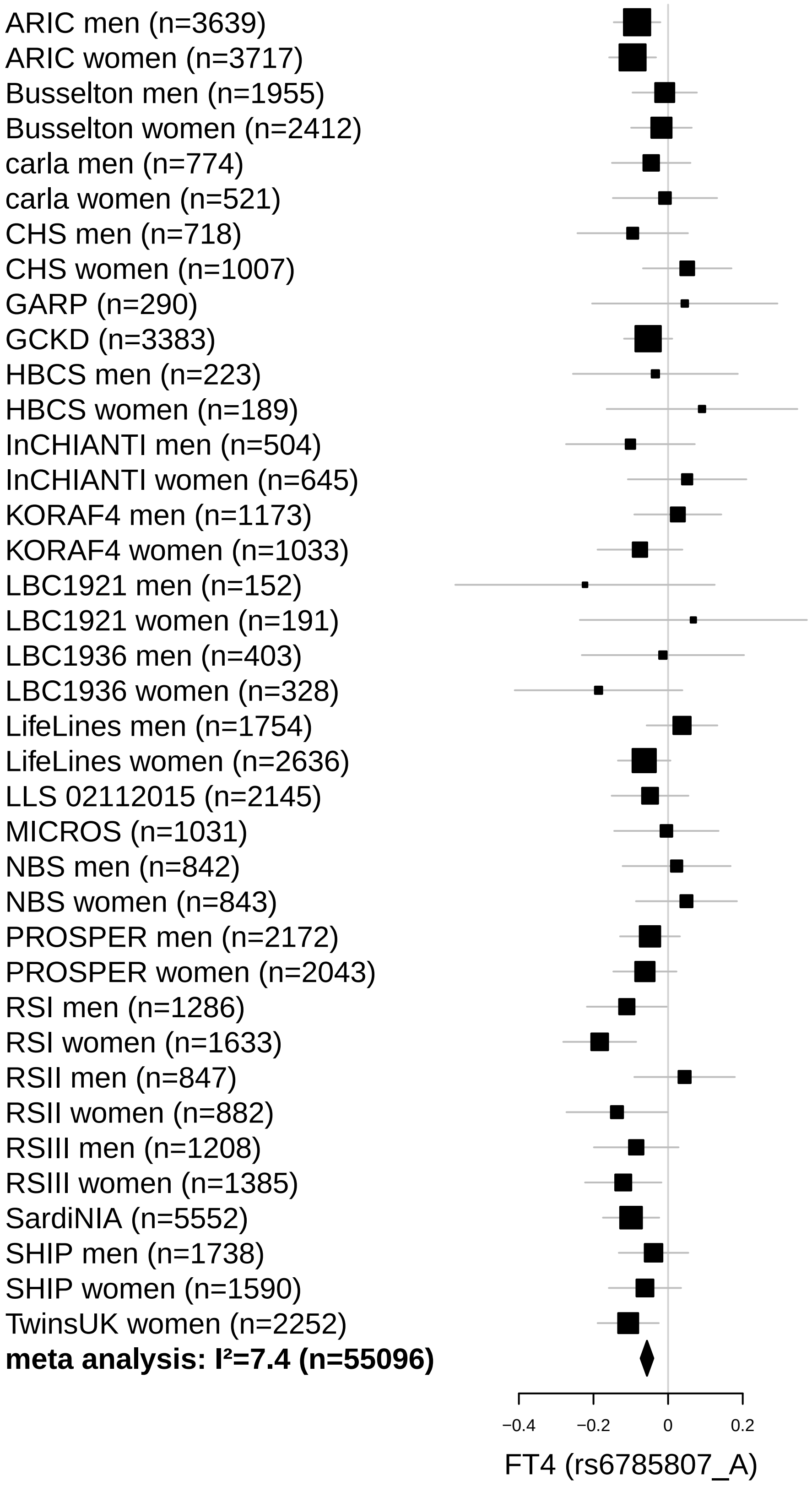
ARIC men (n=3639)
ARIC women (n=3717)
Busselton men (n=1955)
Busselton women (n=2412)
carla men (n=774)
carla women (n=521)
CHS men (n=718)
CHS women (n=1007)
GARP (n=290)
GCKD (n=3383)
HBCS men (n=223)
HBCS women (n=189)
InCHIANTI men (n=504)
InCHIANTI women (n=645)
KORAF4 men (n=1173)
KORAF4 women (n=1033)
LBC1921 men (n=152)
LBC1921 women (n=191)
LBC1936 men (n=403)
LBC1936 women (n=328)
LifeLines men (n=1754)
LifeLines women (n=2636)
LLS 02112015 (n=2145)
MICROS (n=1031)
NBS men (n=842)
NBS women (n=843)
PROSPER men (n=2172)
PROSPER women (n=2043)
RSI men (n=1286)
RSI women (n=1633)
RSII men (n=847)
RSII women (n=882)
RSIII men (n=1208)
RSIII women (n=1385)
SardiNIA (n=5552)
SHIP men (n=1738)
SHIP women (n=1590)
TwinsUK women (n=2252)
meta analysis: I²=7.4 (n=55096)
−0.4
−0.2
0
0.2
FT4 (rs6785807_A)

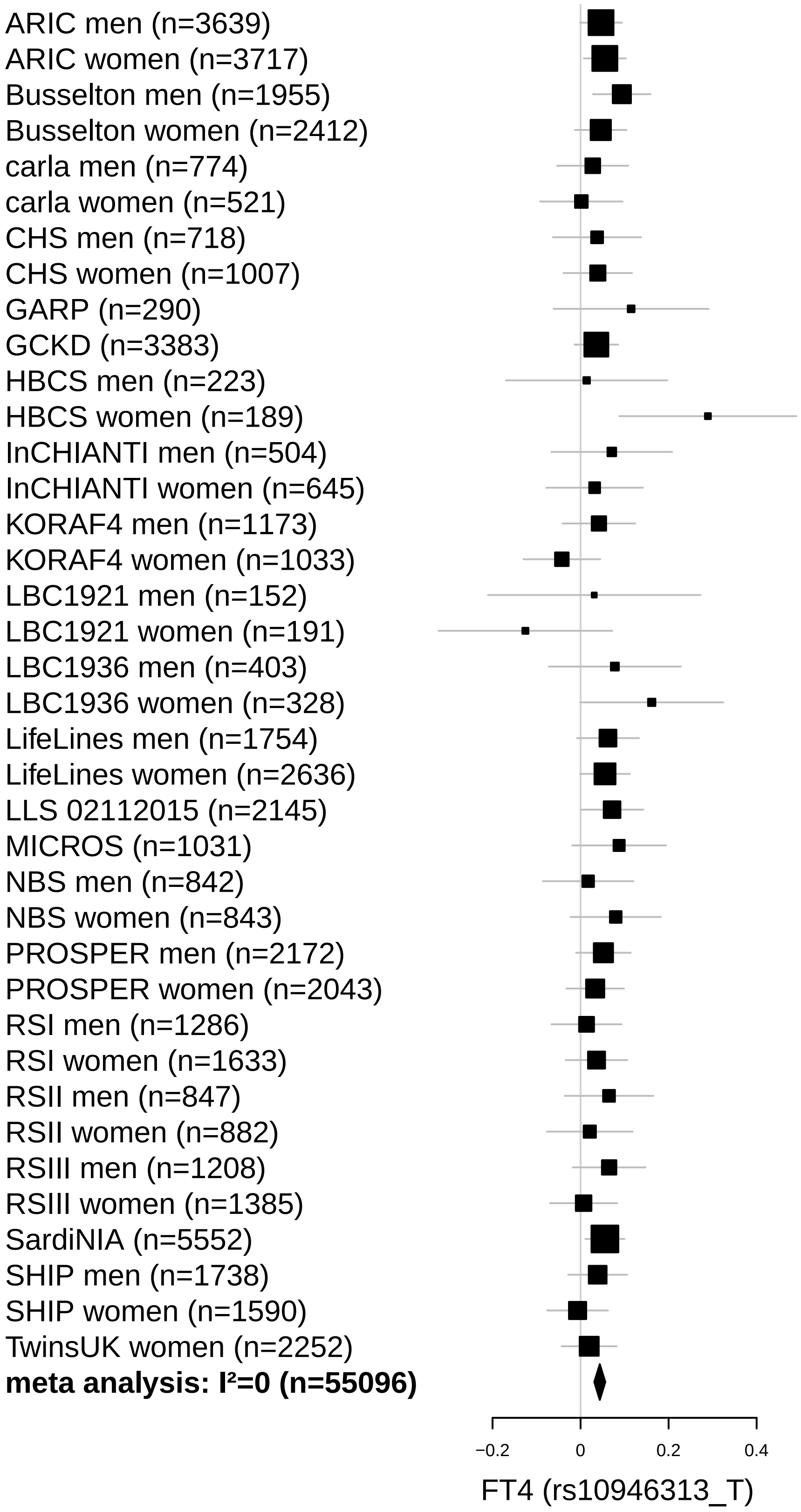
ARIC men (n=3639)
ARIC women (n=3717)
Busselton men (n=1955)
Busselton women (n=2412)
carla men (n=774)
carla women (n=521)
CHS men (n=718)
CHS women (n=1007)
GARP (n=290)
GCKD (n=3383)
HBCS men (n=223)
HBCS women (n=189)
InCHIANTI men (n=504)
InCHIANTI women (n=645)
KORAF4 men (n=1173)
KORAF4 women (n=1033)
LBC1921 men (n=152)
LBC1921 women (n=191)
LBC1936 men (n=403)
LBC1936 women (n=328)
LifeLines men (n=1754)
LifeLines women (n=2636)
LLS 02112015 (n=2145)
MICROS (n=1031)
NBS men (n=842)
NBS women (n=843)
PROSPER men (n=2172)
PROSPER women (n=2043)
RSI men (n=1286)
RSI women (n=1633)
RSII men (n=847)
RSII women (n=882)
RSIII men (n=1208)
RSIII women (n=1385)
SardiNIA (n=5552)
SHIP men (n=1738)
SHIP women (n=1590)
TwinsUK women (n=2252)
meta analysis: I²=0 (n=55096)
−0.2
0
0.2
0.4
FT4 (rs10946313_T)

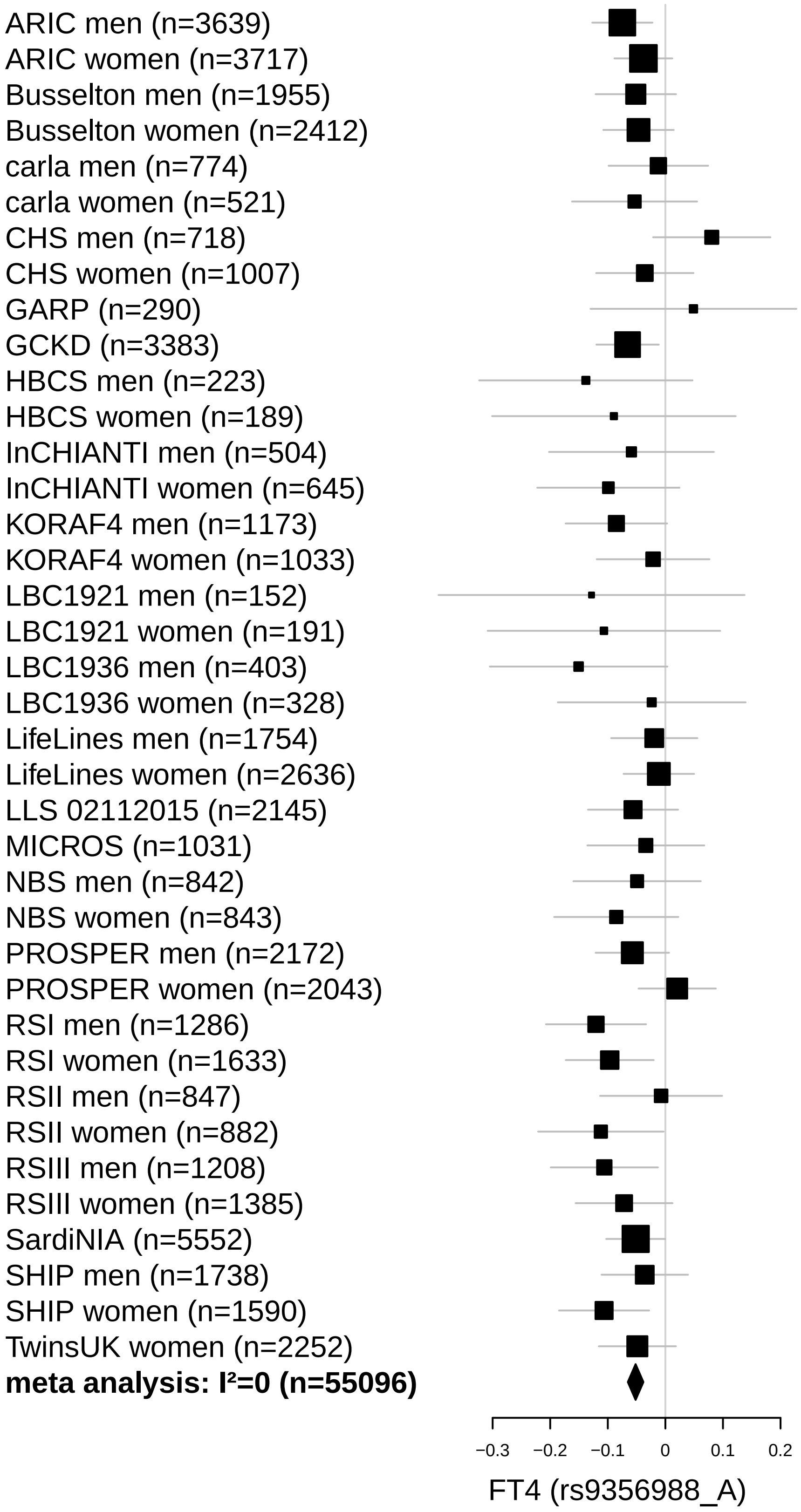
ARIC men (n=3639)
ARIC women (n=3717)
Busselton men (n=1955)
Busselton women (n=2412)
carla men (n=774)
carla women (n=521)
CHS men (n=718)
CHS women (n=1007)
GARP (n=290)
GCKD (n=3383)
HBCS men (n=223)
HBCS women (n=189)
InCHIANTI men (n=504)
InCHIANTI women (n=645)
KORAF4 men (n=1173)
KORAF4 women (n=1033)
LBC1921 men (n=152)
LBC1921 women (n=191)
LBC1936 men (n=403)
LBC1936 women (n=328)
LifeLines men (n=1754)
LifeLines women (n=2636)
LLS 02112015 (n=2145)
MICROS (n=1031)
NBS men (n=842)
NBS women (n=843)
PROSPER men (n=2172)
PROSPER women (n=2043)
RSI men (n=1286)
RSI women (n=1633)
RSII men (n=847)
RSII women (n=882)
RSIII men (n=1208)
RSIII women (n=1385)
SardiNIA (n=5552)
SHIP men (n=1738)
SHIP women (n=1590)
TwinsUK women (n=2252)
meta analysis: I²=0 (n=55096)
−0.3
−0.2
−0.1
0
0.1
0.2
FT4 (rs9356988_A)

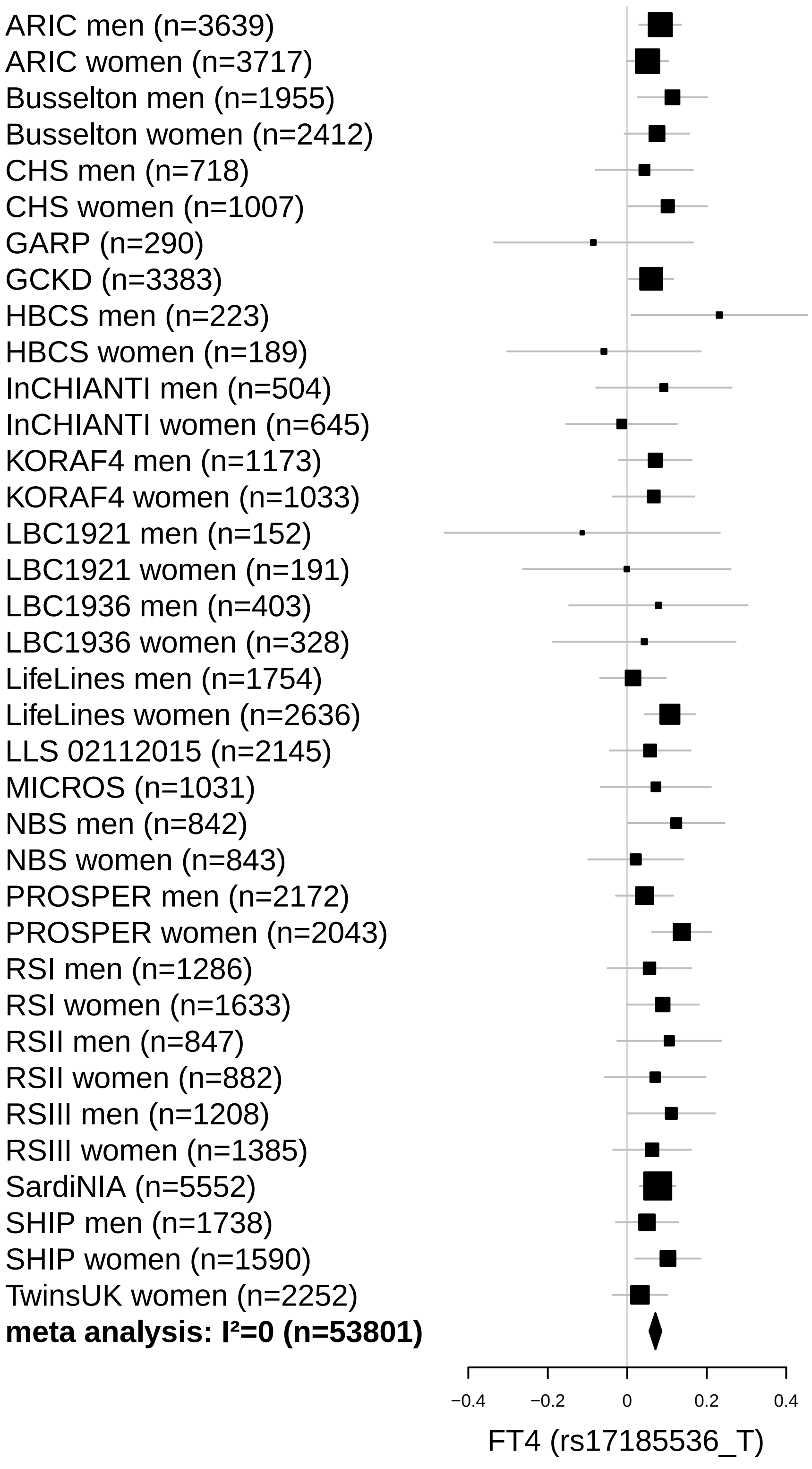
ARIC men (n=3639)
ARIC women (n=3717)
Busselton men (n=1955)
Busselton women (n=2412)
CHS men (n=718)
CHS women (n=1007)
GARP (n=290)
GCKD (n=3383)
HBCS men (n=223)
HBCS women (n=189)
InCHIANTI men (n=504)
InCHIANTI women (n=645)
KORAF4 men (n=1173)
KORAF4 women (n=1033)
LBC1921 men (n=152)
LBC1921 women (n=191)
LBC1936 men (n=403)
LBC1936 women (n=328)
LifeLines men (n=1754)
LifeLines women (n=2636)
LLS 02112015 (n=2145)
MICROS (n=1031)
NBS men (n=842)
NBS women (n=843)
PROSPER men (n=2172)
PROSPER women (n=2043)
RSI men (n=1286)
RSI women (n=1633)
RSII men (n=847)
RSII women (n=882)
RSIII men (n=1208)
RSIII women (n=1385)
SardiNIA (n=5552)
SHIP men (n=1738)
SHIP women (n=1590)
TwinsUK women (n=2252)
meta analysis: I²=0 (n=53801)
−0.4
−0.2
0
0.2
0.4
FT4 (rs17185536_T)

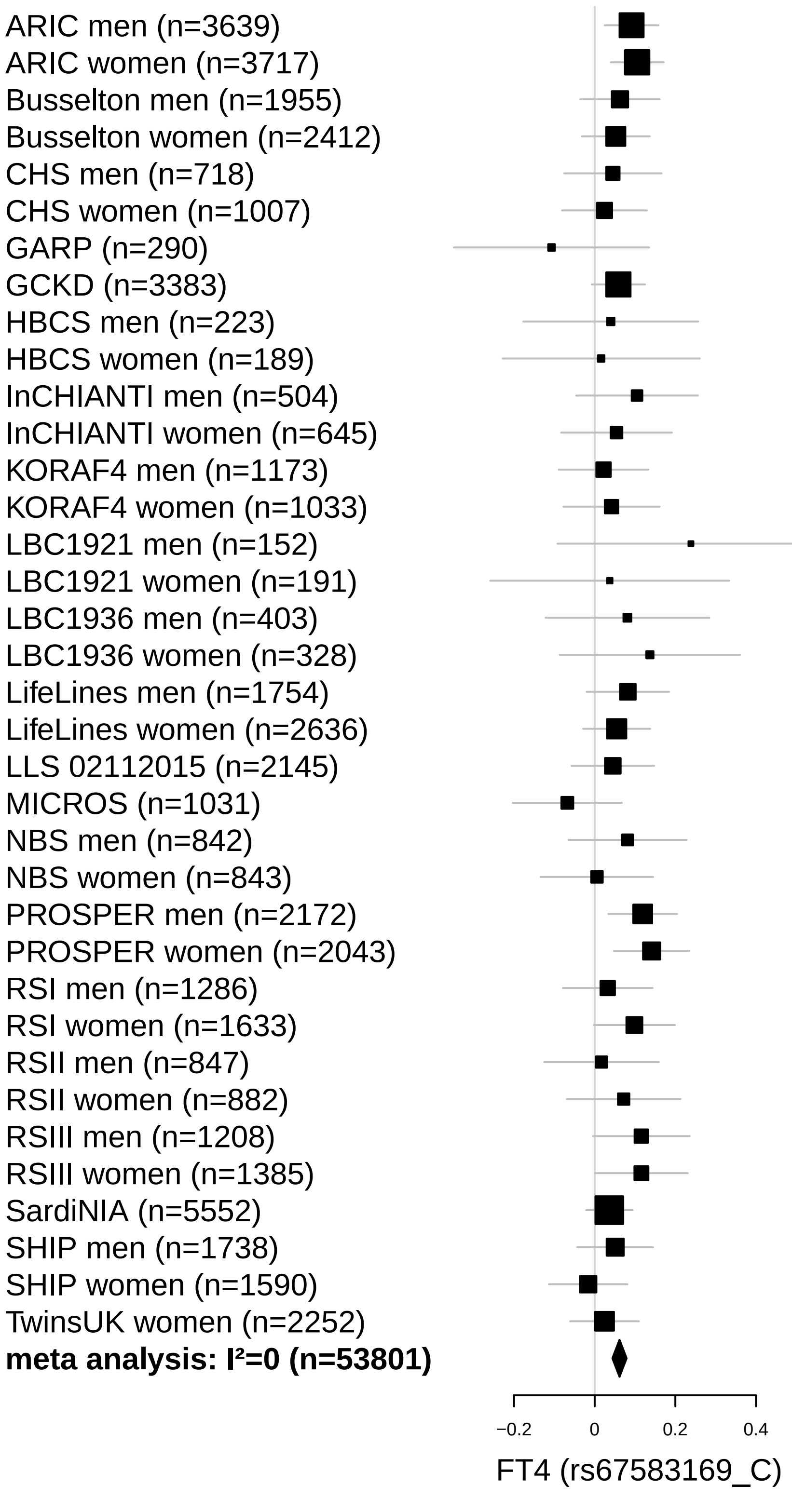
ARIC men (n=3639)
ARIC women (n=3717)
Busselton men (n=1955)
Busselton women (n=2412)
CHS men (n=718)
CHS women (n=1007)
GARP (n=290)
GCKD (n=3383)
HBCS men (n=223)
HBCS women (n=189)
InCHIANTI men (n=504)
InCHIANTI women (n=645)
KORAF4 men (n=1173)
KORAF4 women (n=1033)
LBC1921 men (n=152)
LBC1921 women (n=191)
LBC1936 men (n=403)
LBC1936 women (n=328)
LifeLines men (n=1754)
LifeLines women (n=2636)
LLS 02112015 (n=2145)
MICROS (n=1031)
NBS men (n=842)
NBS women (n=843)
PROSPER men (n=2172)
PROSPER women (n=2043)
RSI men (n=1286)
RSI women (n=1633)
RSII men (n=847)
RSII women (n=882)
RSIII men (n=1208)
RSIII women (n=1385)
SardiNIA (n=5552)
SHIP men (n=1738)
SHIP women (n=1590)
TwinsUK women (n=2252)
meta analysis: I²=0 (n=53801)
−0.2
0
0.2
0.4
FT4 (rs67583169_C)

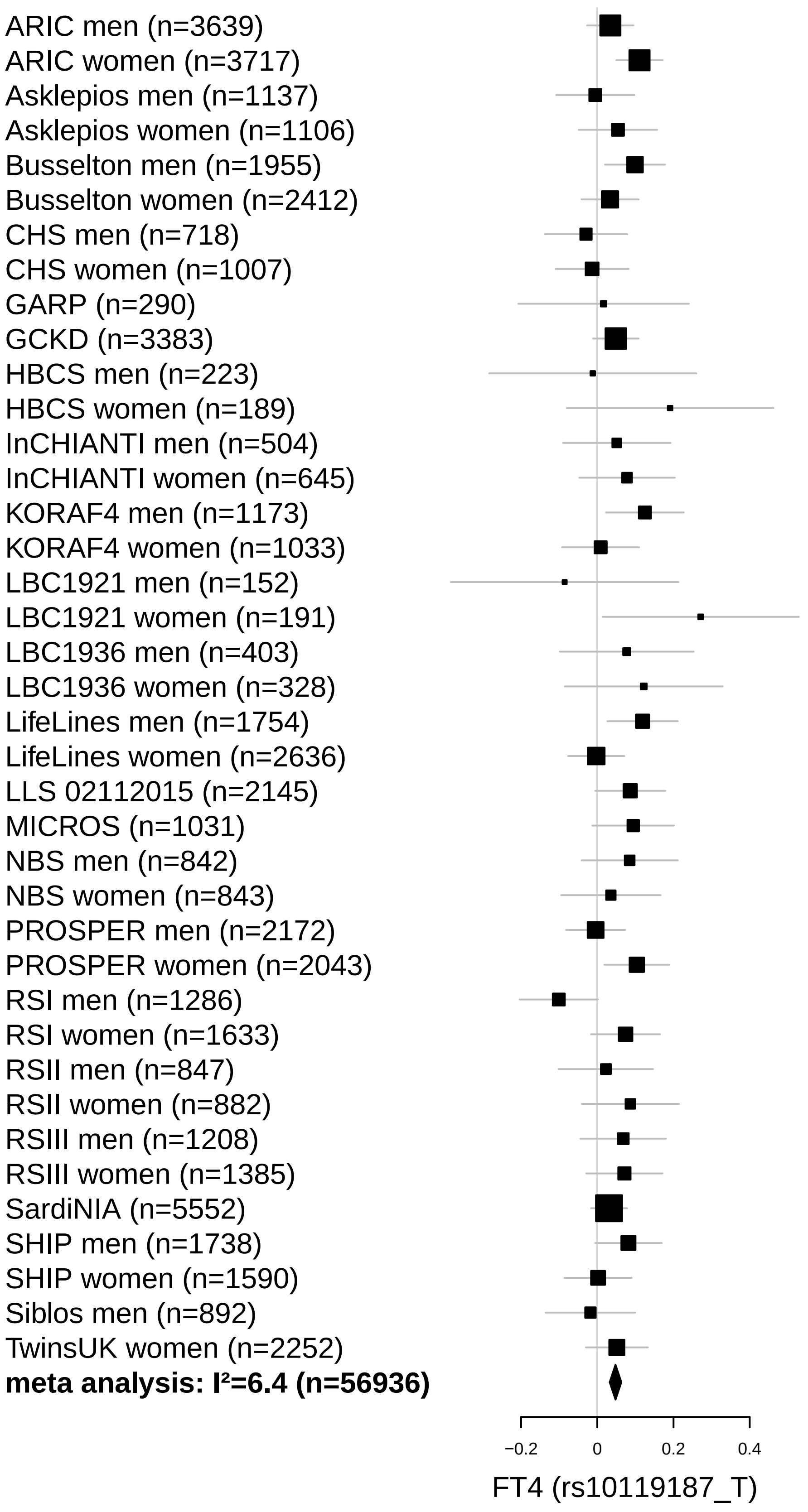
ARIC men (n=3639)
ARIC women (n=3717)
Asklepios men (n=1137)
Asklepios women (n=1106)
Busselton men (n=1955)
Busselton women (n=2412)
CHS men (n=718)
CHS women (n=1007)
GARP (n=290)
GCKD (n=3383)
HBCS men (n=223)
HBCS women (n=189)
InCHIANTI men (n=504)
InCHIANTI women (n=645)
KORAF4 men (n=1173)
KORAF4 women (n=1033)
LBC1921 men (n=152)
LBC1921 women (n=191)
LBC1936 men (n=403)
LBC1936 women (n=328)
LifeLines men (n=1754)
LifeLines women (n=2636)
LLS 02112015 (n=2145)
MICROS (n=1031)
NBS men (n=842)
NBS women (n=843)
PROSPER men (n=2172)
PROSPER women (n=2043)
RSI men (n=1286)
RSI women (n=1633)
RSII men (n=847)
RSII women (n=882)
RSIII men (n=1208)
RSIII women (n=1385)
SardiNIA (n=5552)
SHIP men (n=1738)
SHIP women (n=1590)
Siblos men (n=892)
TwinsUK women (n=2252)
meta analysis: I²=6.4 (n=56936)
−0.2
0
0.2
0.4
FT4 (rs10119187_T)

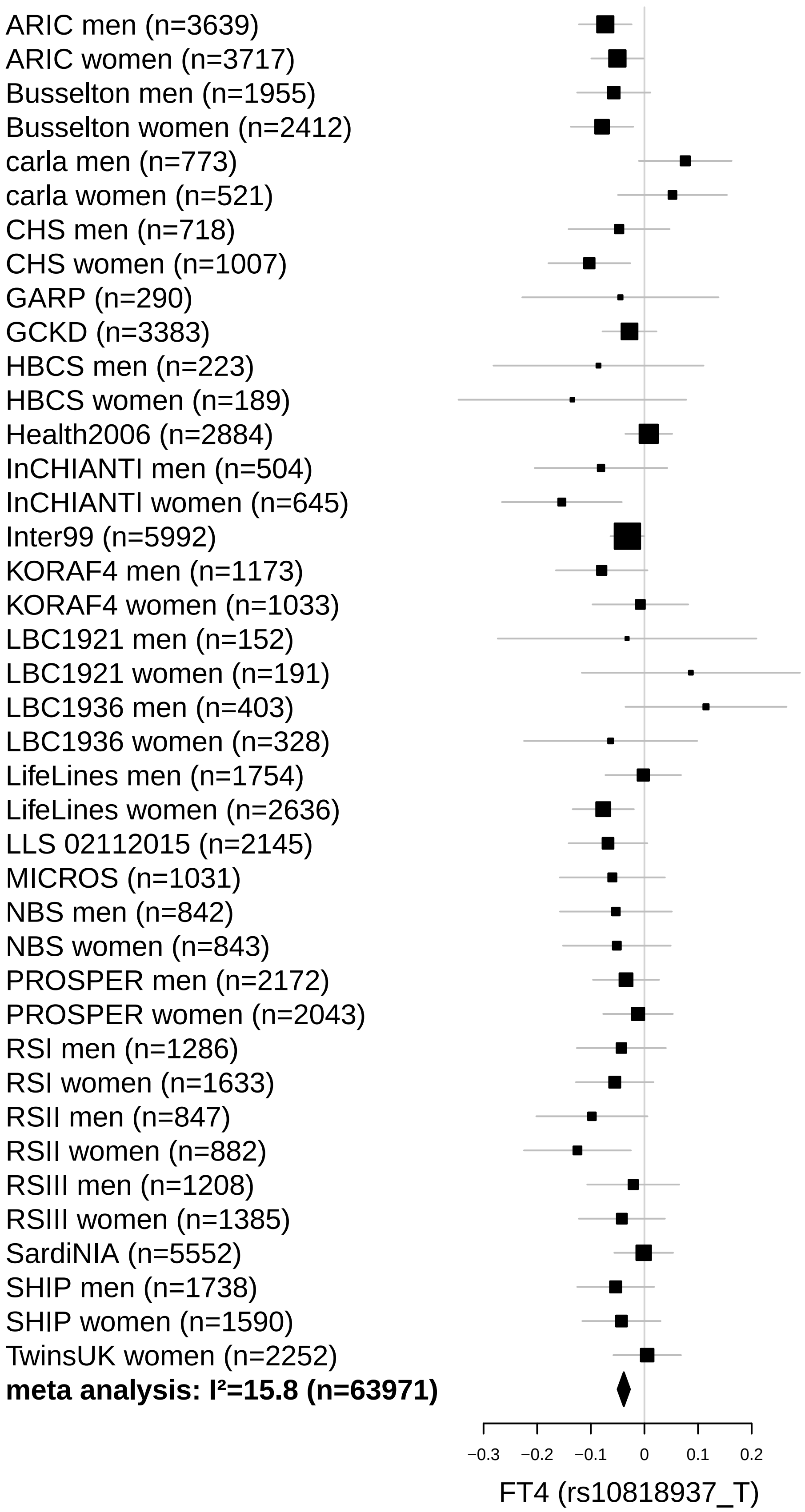
ARIC men (n=3639)
ARIC women (n=3717)
Busselton men (n=1955)
Busselton women (n=2412)
carla men (n=773)
carla women (n=521)
CHS men (n=718)
CHS women (n=1007)
GARP (n=290)
GCKD (n=3383)
HBCS men (n=223)
HBCS women (n=189)
Health2006 (n=2884)
InCHIANTI men (n=504)
InCHIANTI women (n=645)
Inter99 (n=5992)
KORAF4 men (n=1173)
KORAF4 women (n=1033)
LBC1921 men (n=152)
LBC1921 women (n=191)
LBC1936 men (n=403)
LBC1936 women (n=328)
LifeLines men (n=1754)
LifeLines women (n=2636)
LLS 02112015 (n=2145)
MICROS (n=1031)
NBS men (n=842)
NBS women (n=843)
PROSPER men (n=2172)
PROSPER women (n=2043)
RSI men (n=1286)
RSI women (n=1633)
RSII men (n=847)
RSII women (n=882)
RSIII men (n=1208)
RSIII women (n=1385)
SardiNIA (n=5552)
SHIP men (n=1738)
SHIP women (n=1590)
TwinsUK women (n=2252)
meta analysis: I²=15.8 (n=63971)
−0.3
−0.2
−0.1
0
0.1
0.2
FT4 (rs10818937_T)

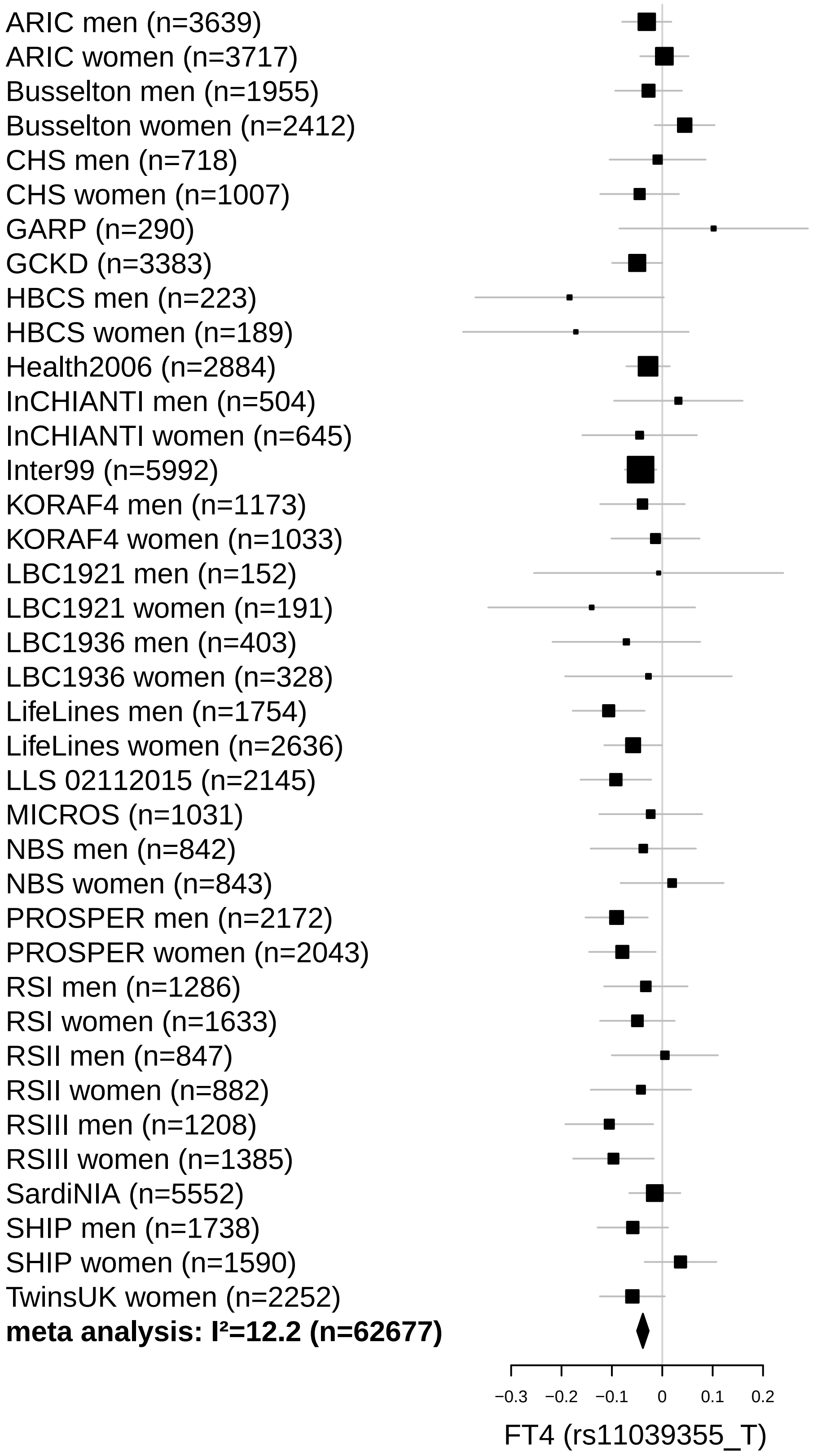
ARIC men (n=3639)
ARIC women (n=3717)
Busselton men (n=1955)
Busselton women (n=2412)
CHS men (n=718)
CHS women (n=1007)
GARP (n=290)
GCKD (n=3383)
HBCS men (n=223)
HBCS women (n=189)
Health2006 (n=2884)
InCHIANTI men (n=504)
InCHIANTI women (n=645)
Inter99 (n=5992)
KORAF4 men (n=1173)
KORAF4 women (n=1033)
LBC1921 men (n=152)
LBC1921 women (n=191)
LBC1936 men (n=403)
LBC1936 women (n=328)
LifeLines men (n=1754)
LifeLines women (n=2636)
LLS 02112015 (n=2145)
MICROS (n=1031)
NBS men (n=842)
NBS women (n=843)
PROSPER men (n=2172)
PROSPER women (n=2043)
RSI men (n=1286)
RSI women (n=1633)
RSII men (n=847)
RSII women (n=882)
RSIII men (n=1208)
RSIII women (n=1385)
SardiNIA (n=5552)
SHIP men (n=1738)
SHIP women (n=1590)
TwinsUK women (n=2252)
meta analysis: I²=12.2 (n=62677)
−0.3
−0.2
−0.1
0
0.1
0.2
FT4 (rs11039355_T)

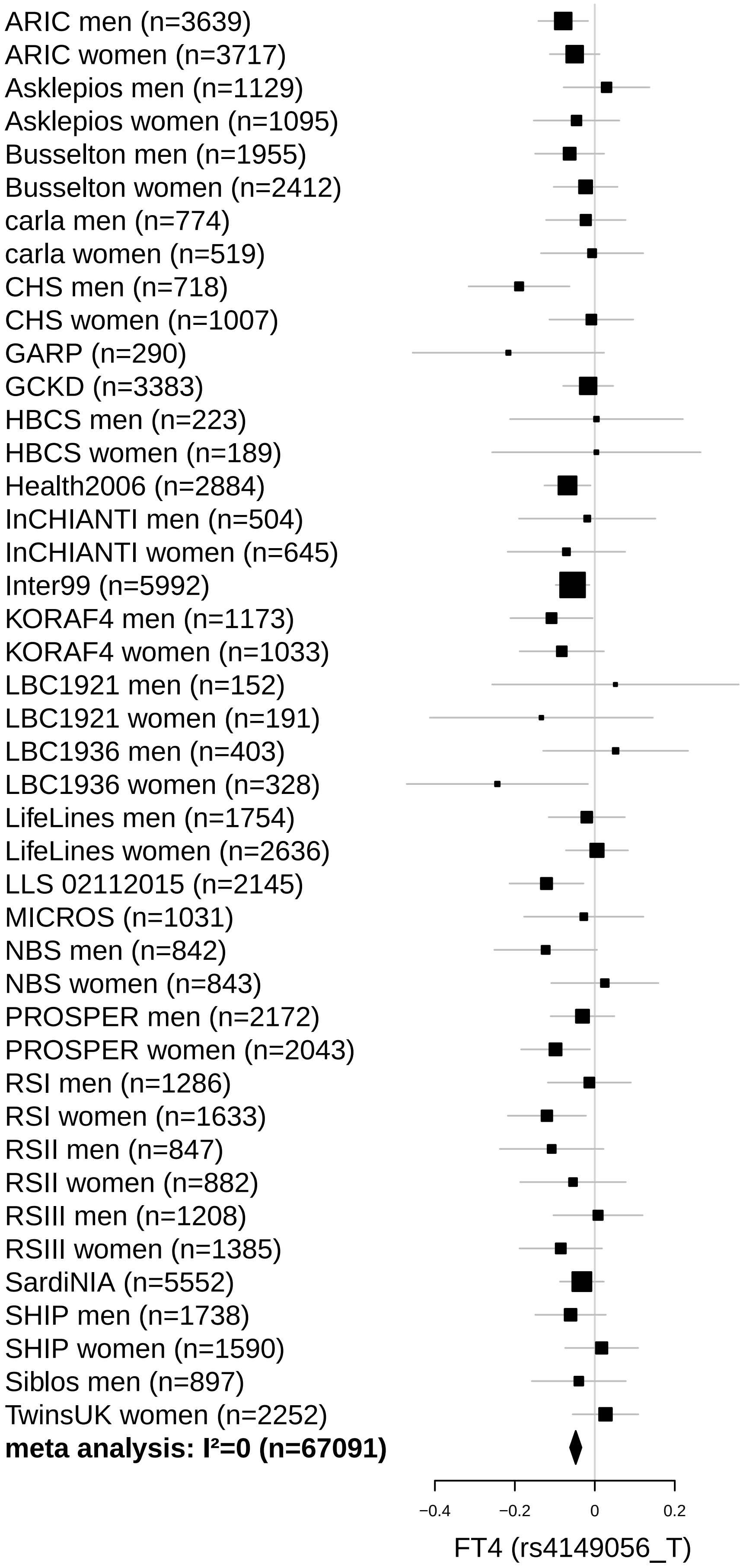

ARIC men (n=3639)
ARIC women (n=3717)
Asklepios men (n=1129)
Asklepios women (n=1095)
Busselton men (n=1955)
Busselton women (n=2412)
carla men (n=774)
carla women (n=519)
CHS men (n=718)
CHS women (n=1007)
GARP (n=290)
GCKD (n=3383)
HBCS men (n=223)
HBCS women (n=189)
Health2006 (n=2884)
InCHIANTI men (n=504)
InCHIANTI women (n=645)
Inter99 (n=5992)
KORAF4 men (n=1173)
KORAF4 women (n=1033)
LBC1921 men (n=152)
LBC1921 women (n=191)
LBC1936 men (n=403)
LBC1936 women (n=328)
LifeLines men (n=1754)
LifeLines women (n=2636)
LLS 02112015 (n=2145)
MICROS (n=1031)
NBS men (n=842)
NBS women (n=843)
PROSPER men (n=2172)
PROSPER women (n=2043)
RSI men (n=1286)
RSI women (n=1633)
RSII men (n=847)
RSII women (n=882)
RSIII men (n=1208)
RSIII women (n=1385)
SardiNIA (n=5552)
SHIP men (n=1738)
SHIP women (n=1590)
Siblos men (n=897)
TwinsUK women (n=2252)
meta analysis: I²=0 (n=67091)
−0.4
−0.2
0
0.2
FT4 (rs4149056_T)

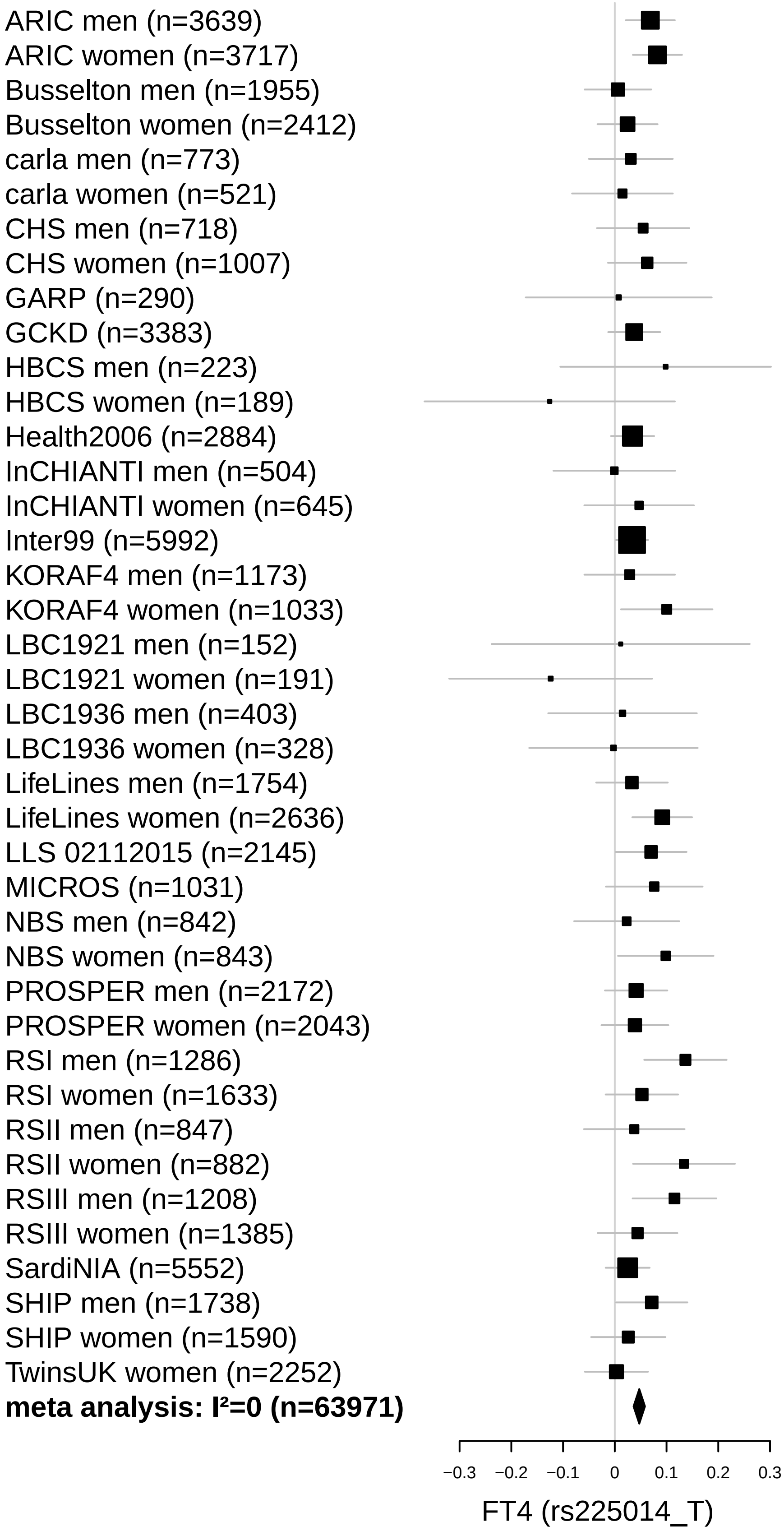

ARIC men (n=3639)
ARIC women (n=3717)
Busselton men (n=1955)
Busselton women (n=2412)
carla men (n=773)
carla women (n=521)
CHS men (n=718)
CHS women (n=1007)
GARP (n=290)
GCKD (n=3383)
HBCS men (n=223)
HBCS women (n=189)
Health2006 (n=2884)
InCHIANTI men (n=504)
InCHIANTI women (n=645)
Inter99 (n=5992)
KORAF4 men (n=1173)
KORAF4 women (n=1033)
LBC1921 men (n=152)
LBC1921 women (n=191)
LBC1936 men (n=403)
LBC1936 women (n=328)
LifeLines men (n=1754)
LifeLines women (n=2636)
LLS 02112015 (n=2145)
MICROS (n=1031)
NBS men (n=842)
NBS women (n=843)
PROSPER men (n=2172)
PROSPER women (n=2043)
RSI men (n=1286)
RSI women (n=1633)
RSII men (n=847)
RSII women (n=882)
RSIII men (n=1208)
RSIII women (n=1385)
SardiNIA (n=5552)
SHIP men (n=1738)
SHIP women (n=1590)
TwinsUK women (n=2252)
meta analysis: I²=0 (n=63971)
−0.3
−0.2
−0.1
0
0.1
0.2
0.3
FT4 (rs225014_T)

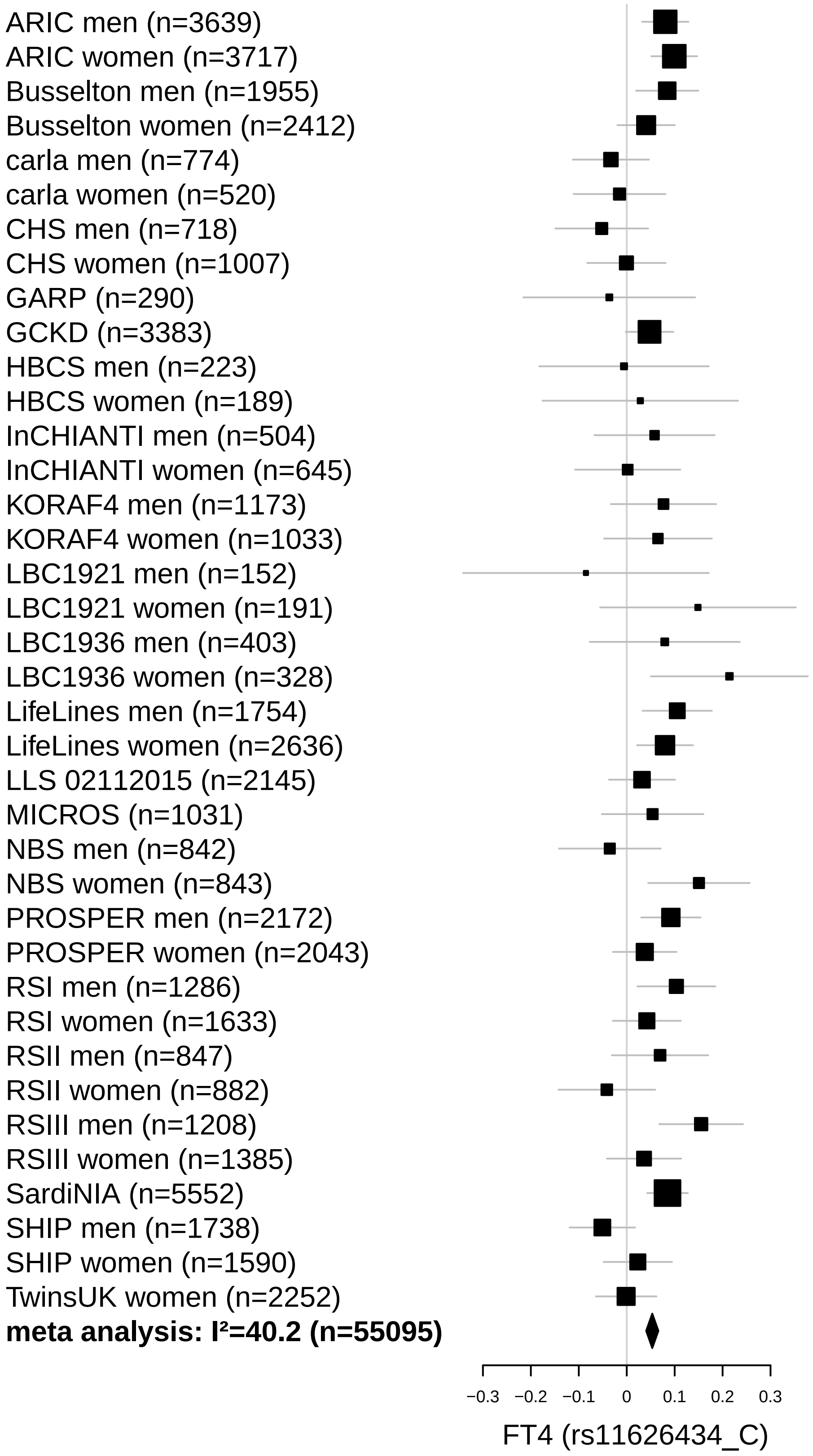
ARIC men (n=3639)
ARIC women (n=3717)
Busselton men (n=1955)
Busselton women (n=2412)
carla men (n=774)
carla women (n=520)
CHS men (n=718)
CHS women (n=1007)
GARP (n=290)
GCKD (n=3383)
HBCS men (n=223)
HBCS women (n=189)
InCHIANTI men (n=504)
InCHIANTI women (n=645)
KORAF4 men (n=1173)
KORAF4 women (n=1033)
LBC1921 men (n=152)
LBC1921 women (n=191)
LBC1936 men (n=403)
LBC1936 women (n=328)
LifeLines men (n=1754)
LifeLines women (n=2636)
LLS 02112015 (n=2145)
MICROS (n=1031)
NBS men (n=842)
NBS women (n=843)
PROSPER men (n=2172)
PROSPER women (n=2043)
RSI men (n=1286)
RSI women (n=1633)
RSII men (n=847)
RSII women (n=882)
RSIII men (n=1208)
RSIII women (n=1385)
SardiNIA (n=5552)
SHIP men (n=1738)
SHIP women (n=1590)
TwinsUK women (n=2252)
meta analysis: I²=40.2 (n=55095)
−0.3
−0.2
−0.1
0
0.1
0.2
0.3
FT4 (rs11626434_C)

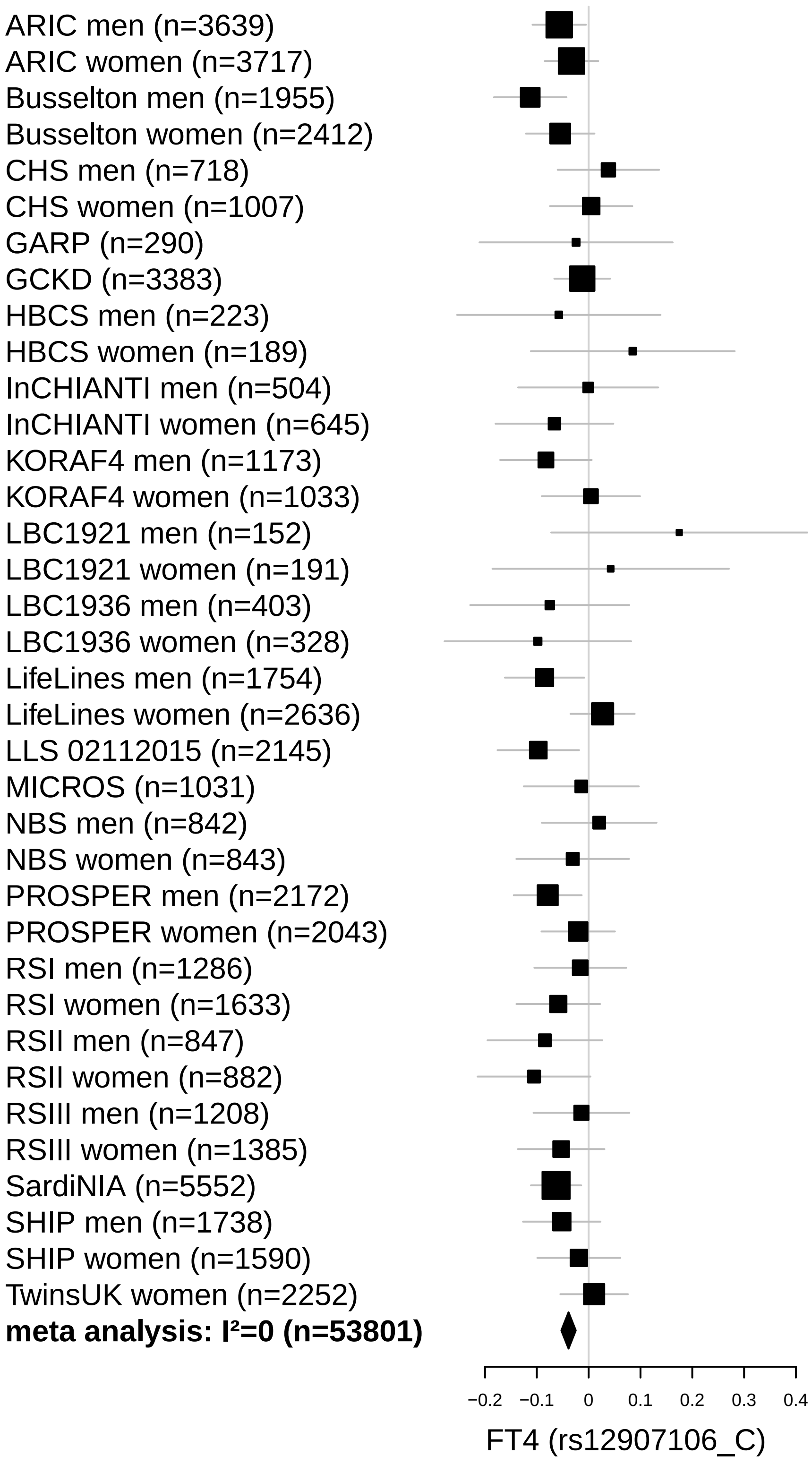
ARIC men (n=3639)
ARIC women (n=3717)
Busselton men (n=1955)
Busselton women (n=2412)
CHS men (n=718)
CHS women (n=1007)
GARP (n=290)
GCKD (n=3383)
HBCS men (n=223)
HBCS women (n=189)
InCHIANTI men (n=504)
InCHIANTI women (n=645)
KORAF4 men (n=1173)
KORAF4 women (n=1033)
LBC1921 men (n=152)
LBC1921 women (n=191)
LBC1936 men (n=403)
LBC1936 women (n=328)
LifeLines men (n=1754)
LifeLines women (n=2636)
LLS 02112015 (n=2145)
MICROS (n=1031)
NBS men (n=842)
NBS women (n=843)
PROSPER men (n=2172)
PROSPER women (n=2043)
RSI men (n=1286)
RSI women (n=1633)
RSII men (n=847)
RSII women (n=882)
RSIII men (n=1208)
RSIII women (n=1385)
SardiNIA (n=5552)
SHIP men (n=1738)
SHIP women (n=1590)
TwinsUK women (n=2252)
meta analysis: I²=0 (n=53801)
−0.2
−0.1
0
0.1
0.2
0.3
0.4
FT4 (rs12907106_C)

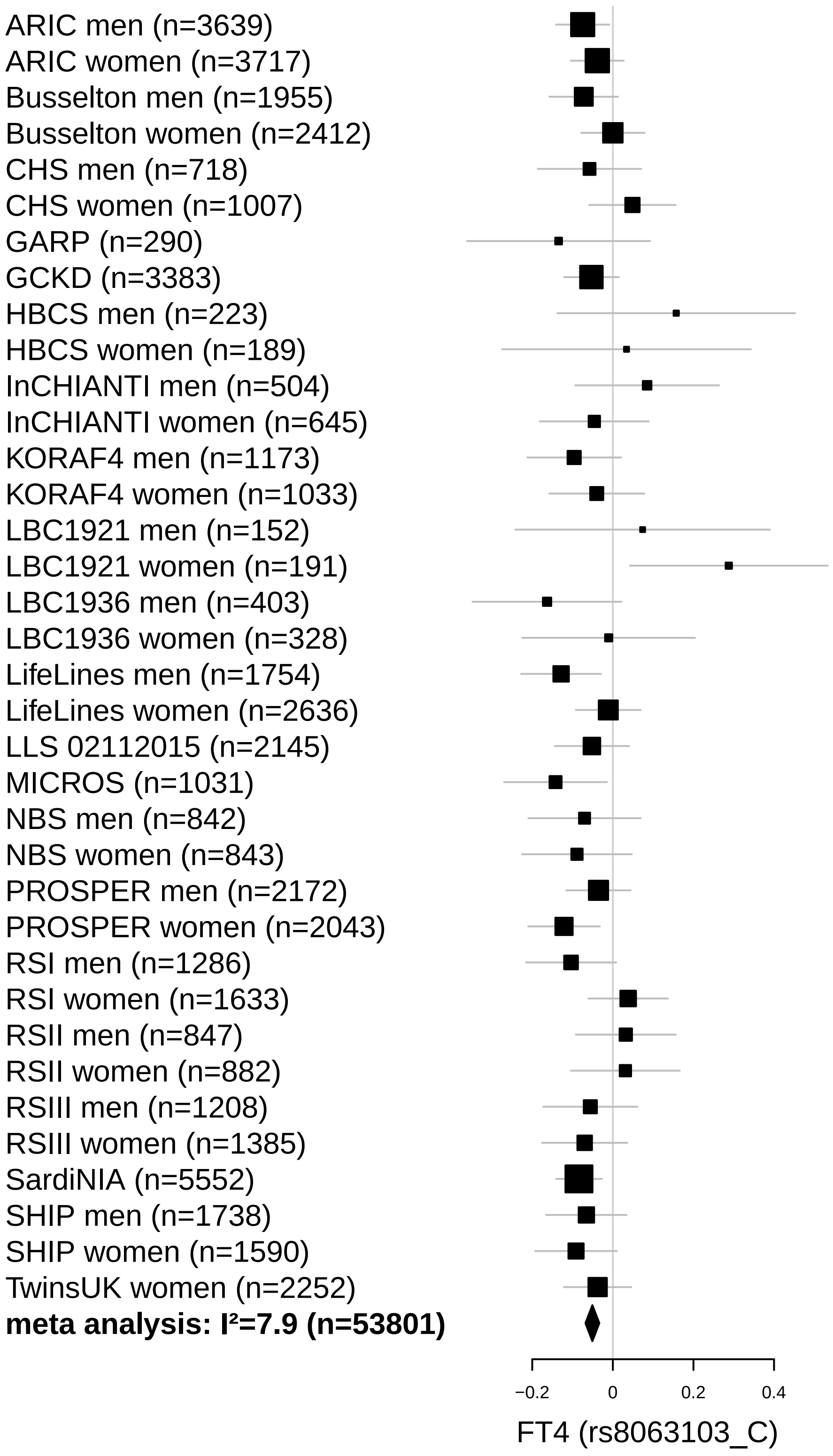

ARIC men (n=3639)
ARIC women (n=3717)
Busselton men (n=1955)
Busselton women (n=2412)
CHS men (n=718)
CHS women (n=1007)
GARP (n=290)
GCKD (n=3383)
HBCS men (n=223)
HBCS women (n=189)
InCHIANTI men (n=504)
InCHIANTI women (n=645)
KORAF4 men (n=1173)
KORAF4 women (n=1033)
LBC1921 men (n=152)
LBC1921 women (n=191)
LBC1936 men (n=403)
LBC1936 women (n=328)
LifeLines men (n=1754)
LifeLines women (n=2636)
LLS 02112015 (n=2145)
MICROS (n=1031)
NBS men (n=842)
NBS women (n=843)
PROSPER men (n=2172)
PROSPER women (n=2043)
RSI men (n=1286)
RSI women (n=1633)
RSII men (n=847)
RSII women (n=882)
RSIII men (n=1208)
RSIII women (n=1385)
SardiNIA (n=5552)
SHIP men (n=1738)
SHIP women (n=1590)
TwinsUK women (n=2252)
meta analysis: I²=7.9 (n=53801)
−0.2
0
0.2
0.4
FT4 (rs8063103_C)

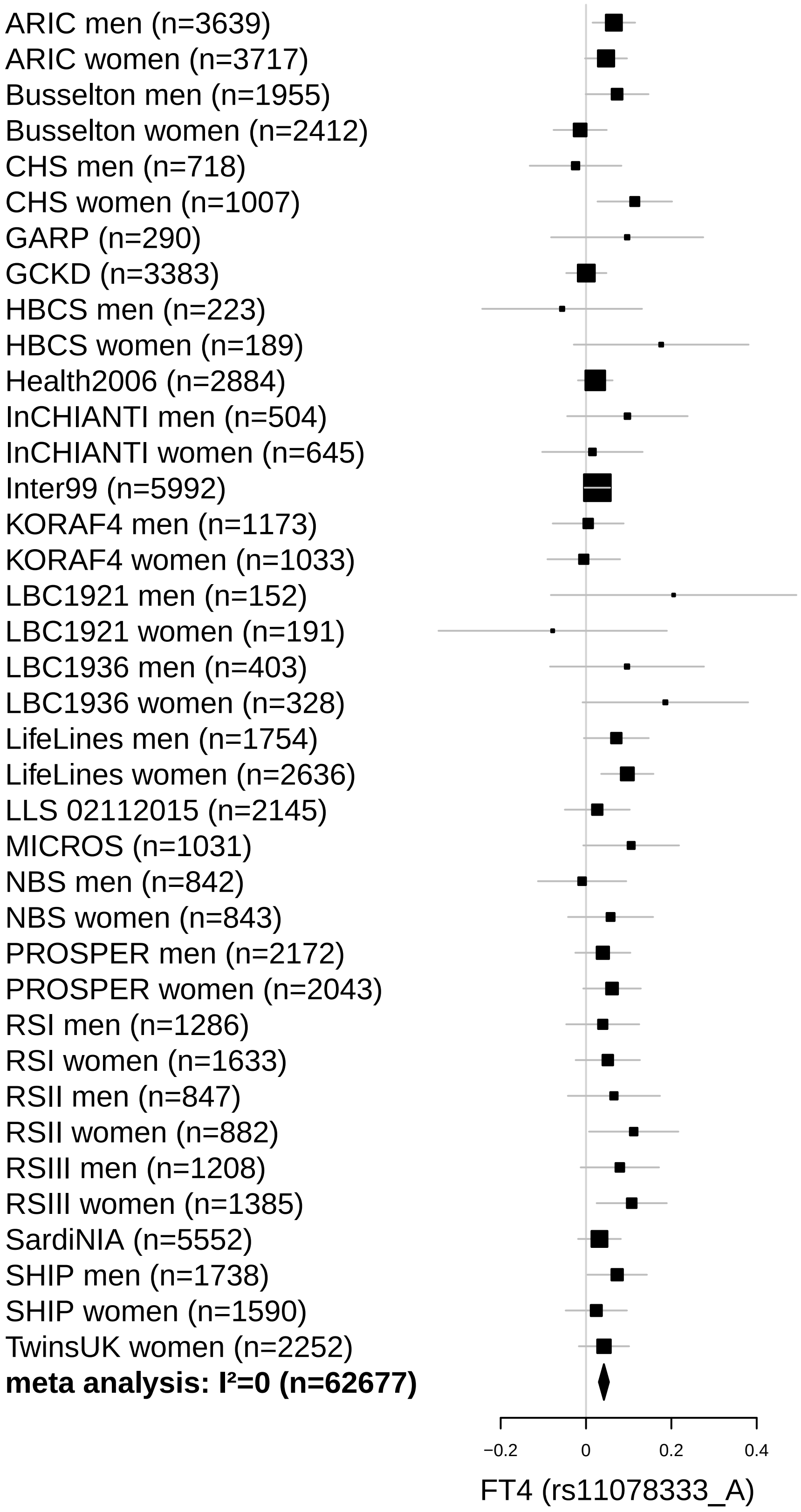
ARIC men (n=3639)
ARIC women (n=3717)
Busselton men (n=1955)
Busselton women (n=2412)
CHS men (n=718)
CHS women (n=1007)
GARP (n=290)
GCKD (n=3383)
HBCS men (n=223)
HBCS women (n=189)
Health2006 (n=2884)
InCHIANTI men (n=504)
InCHIANTI women (n=645)
Inter99 (n=5992)
KORAF4 men (n=1173)
KORAF4 women (n=1033)
LBC1921 men (n=152)
LBC1921 women (n=191)
LBC1936 men (n=403)
LBC1936 women (n=328)
LifeLines men (n=1754)
LifeLines women (n=2636)
LLS 02112015 (n=2145)
MICROS (n=1031)
NBS men (n=842)
NBS women (n=843)
PROSPER men (n=2172)
PROSPER women (n=2043)
RSI men (n=1286)
RSI women (n=1633)
RSII men (n=847)
RSII women (n=882)
RSIII men (n=1208)
RSIII women (n=1385)
SardiNIA (n=5552)
SHIP men (n=1738)
SHIP women (n=1590)
TwinsUK women (n=2252)
meta analysis: I²=0 (n=62677)
−0.2
0
0.2
0.4
FT4 (rs11078333_A)

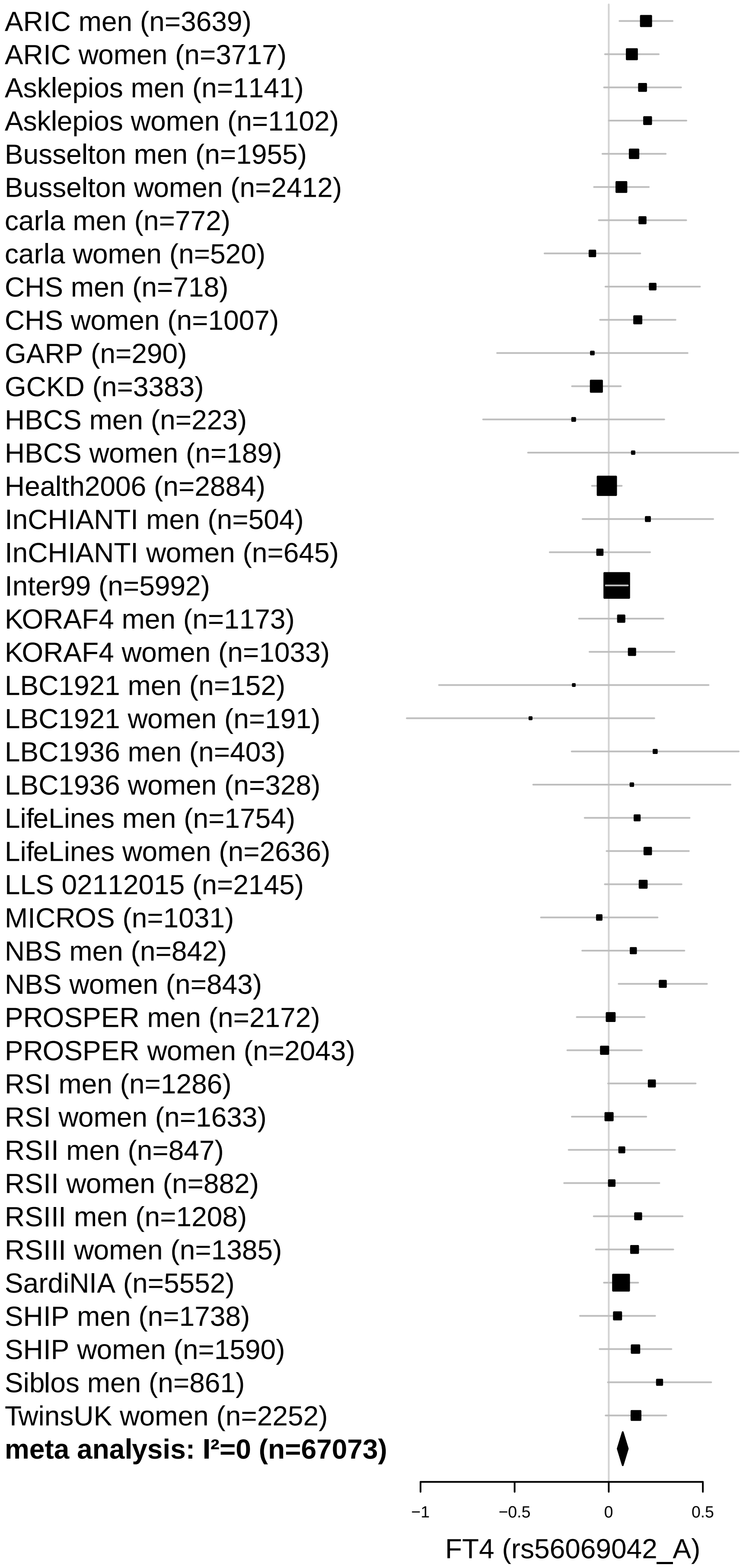
ARIC men (n=3639)
ARIC women (n=3717)
Asklepios men (n=1141)
Asklepios women (n=1102)
Busselton men (n=1955)
Busselton women (n=2412)
carla men (n=772)
carla women (n=520)
CHS men (n=718)
CHS women (n=1007)
GARP (n=290)
GCKD (n=3383)
HBCS men (n=223)
HBCS women (n=189)
Health2006 (n=2884)
InCHIANTI men (n=504)
InCHIANTI women (n=645)
Inter99 (n=5992)
KORAF4 men (n=1173)
KORAF4 women (n=1033)
LBC1921 men (n=152)
LBC1921 women (n=191)
LBC1936 men (n=403)
LBC1936 women (n=328)
LifeLines men (n=1754)
LifeLines women (n=2636)
LLS 02112015 (n=2145)
MICROS (n=1031)
NBS men (n=842)
NBS women (n=843)
PROSPER men (n=2172)
PROSPER women (n=2043)
RSI men (n=1286)
RSI women (n=1633)
RSII men (n=847)
RSII women (n=882)
RSIII men (n=1208)
RSIII women (n=1385)
SardiNIA (n=5552)
SHIP men (n=1738)
SHIP women (n=1590)
Siblos men (n=861)
TwinsUK women (n=2252)
meta analysis: I²=0 (n=67073)
−1
−0.5
0
0.5
FT4 (rs56069042_A)

Forest plots of the 16 index SNPs of replicated susceptibility loci for FT4 in the combined discovery and replication samples. Effects and their 95% confidence intervals are based on inverse normalized FT4 values. I^2 is the percentage of total variation across studies that is due to heterogeneity.

Supplementary Figure 6: Manhattan plots for GWAS meta-analyses in the sex-stratified samples

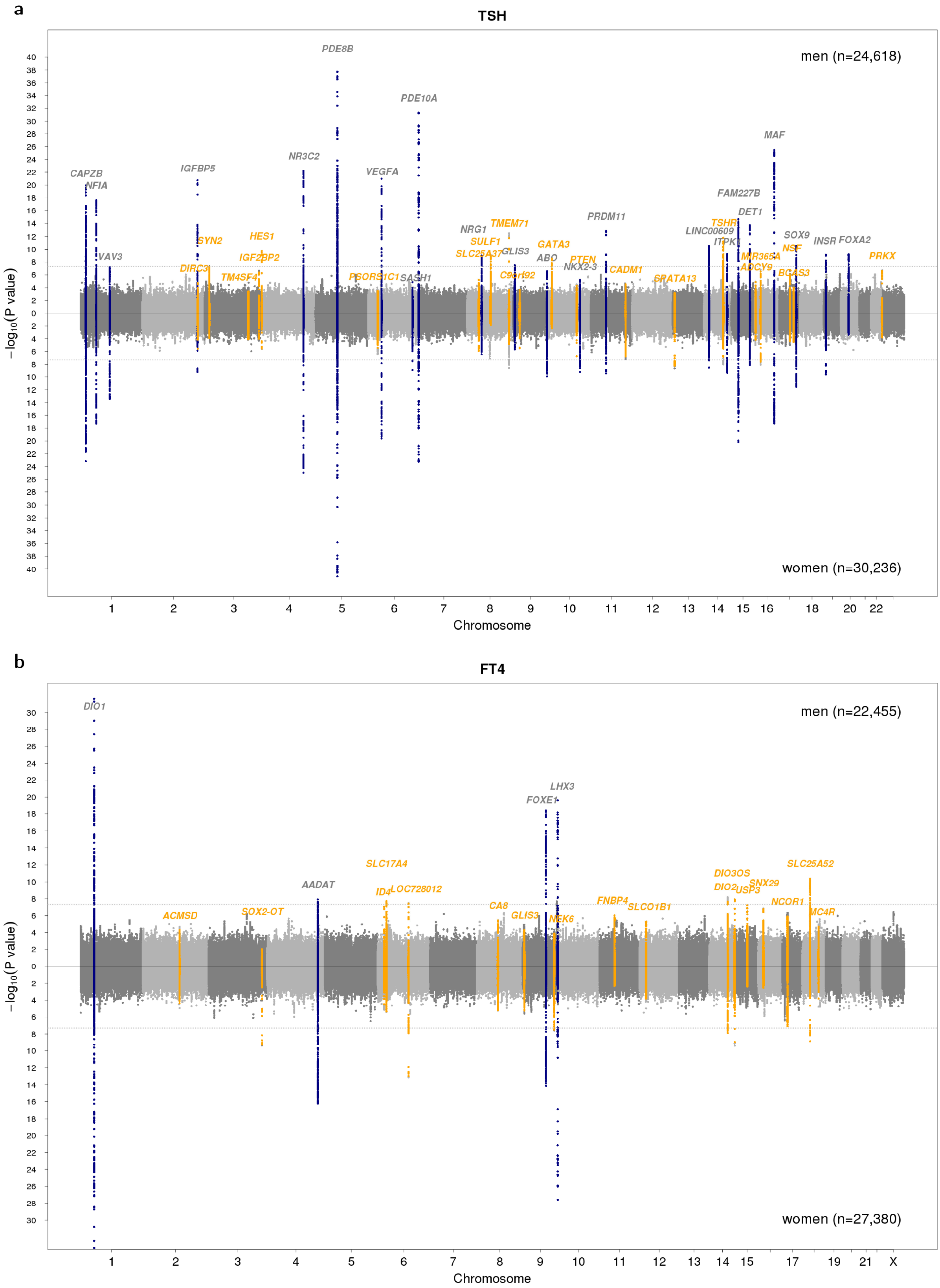
a
TSH
men (n=24,618)
women (n=30,236)
−log10(P value)
Chromosome
PDE8B
PDE10A
MAF
CAPZB
NFIA
IGFBP5
NR3C2
VEGFA
FAM227B
DET1
PRDM11
TSHR
LINC00609
ITPK1
SOX9
INSR
FOXA2
NRG1
TMEM71
SULF1
SLC25A37
GLIS3
GATA3
ABO
PTEN
NKX2-3
CADM1
SPATA13
MIR365A
ADCY9
NSF
BCAS3
PRKX
VAV3
SYN2
HES1
IGF2BP2
DIRC3
TM4SF4
PSORS1C1
SASH1
C9orf92
b
FT4
men (n=22,455)
women (n=27,380)
−log10(P value)
Chromosome
DIO1
LHX3
FOXE1
AADAT
SLC17A4
ID4
LOC728012
ACMSD
SOX2-OT
CA8
GLIS3
NEK6
FNBP4
SLCO1B1
DIO3OS
DIO2
USP3
SNX29
NCOR1
SLC25A52
MC4R

Manhattan plots of the GWAS meta-analysis results for (a) TSH and (b) FT4 contrasted for the men vs. women stratum. SNPs are plotted on the x-axis according to their position on each chromosome with the $-\log_{10}$(p-value) of the association test on the y-axis. The upper solid horizontal line indicates the threshold for genome-wide significance, 5×10^{-8}. Genomic loci previously known to contain trait-associated variants are colored in blue, new findings in orange (based on the overall sample).

Supplementary Figure 7: Genes related to abnormal thyroid morphology in genetically manipulated mice are enriched for SNPs significantly associated with TSH concentrations in humans

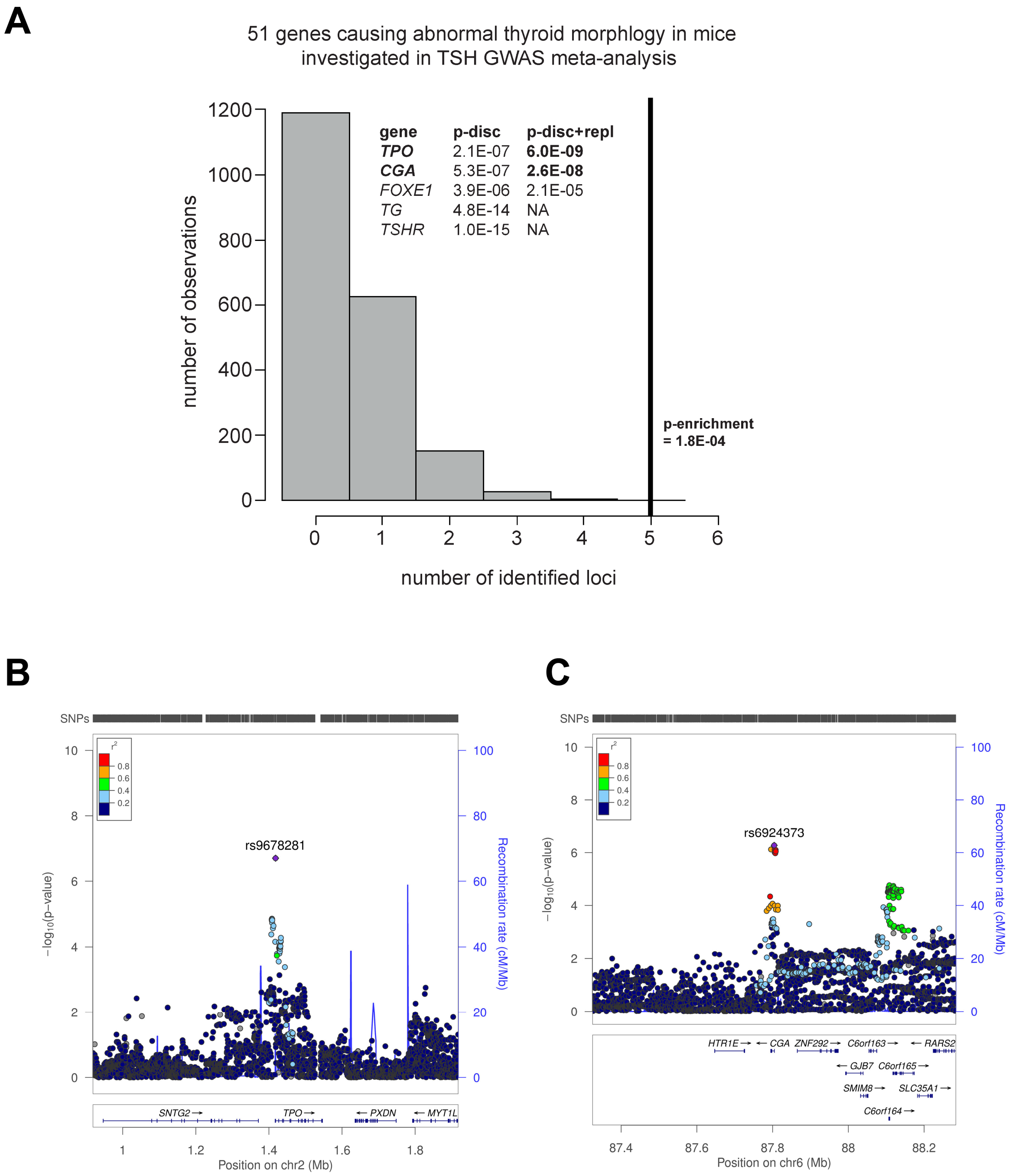

The line indicates the number of observed genes that contain significant SNPs in the TSH meta-analysis based on the human orthologs of 51 genes that cause abnormal thyroid morphology in mice. The histogram depicts the distribution of genes that contain significant SNPs in the TSH meta-analysis based on 2,000 randomly drawn lists of 51 genes. The inset shows the 5 genes that contained significantly associated SNPs in the TSH discovery meta-analysis (p-disc: association test p-value of the index SNP) as well as their evidence for replication in independent samples for SNPs (p-disc+repl: association test p-value in the combined discovery and replication sample) that did not achieve genome-wide significance in the GWAS (Panel A). Panels B and C show the regional association plots for the SNPs that reached genome-wide significance based on the candidate gene approach.

Supplementary Figure 8: Manhattan plot for GWAS meta-analyses for hypo- vs. hyperthyroidism

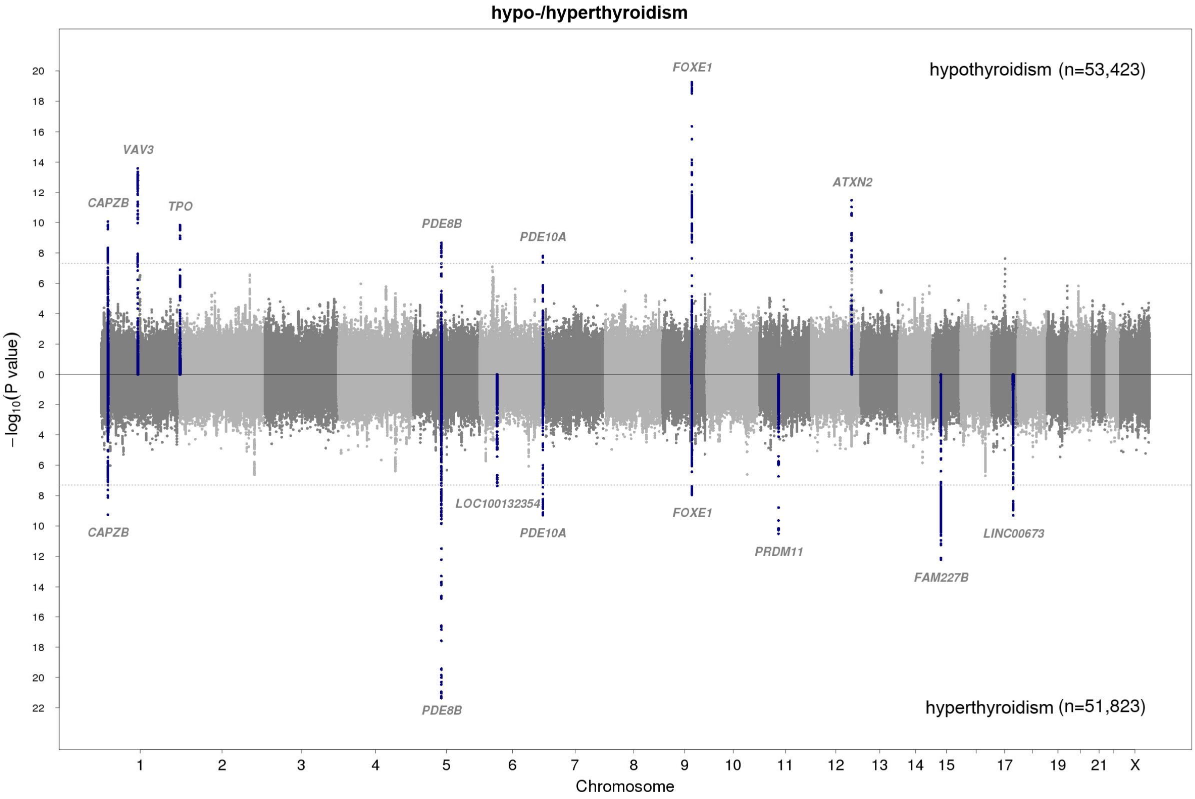

Manhattan plot of the GWAS meta-analysis results for hypo- and hyperthyroidism contrasted with each other. SNPs are plotted on the x-axis according to their position on each chromosome with the -$\log_{10}$(p-value) of the association test on the y-axis. The upper solid horizontal line indicates the threshold for genome-wide significance, $5x10^{-8}$. Genomic loci previously known to contain trait-associated variants are colored in blue.

Supplementary Figure 9: Thyroid hormone transport by MCT8 in comparison to hSLC17A4

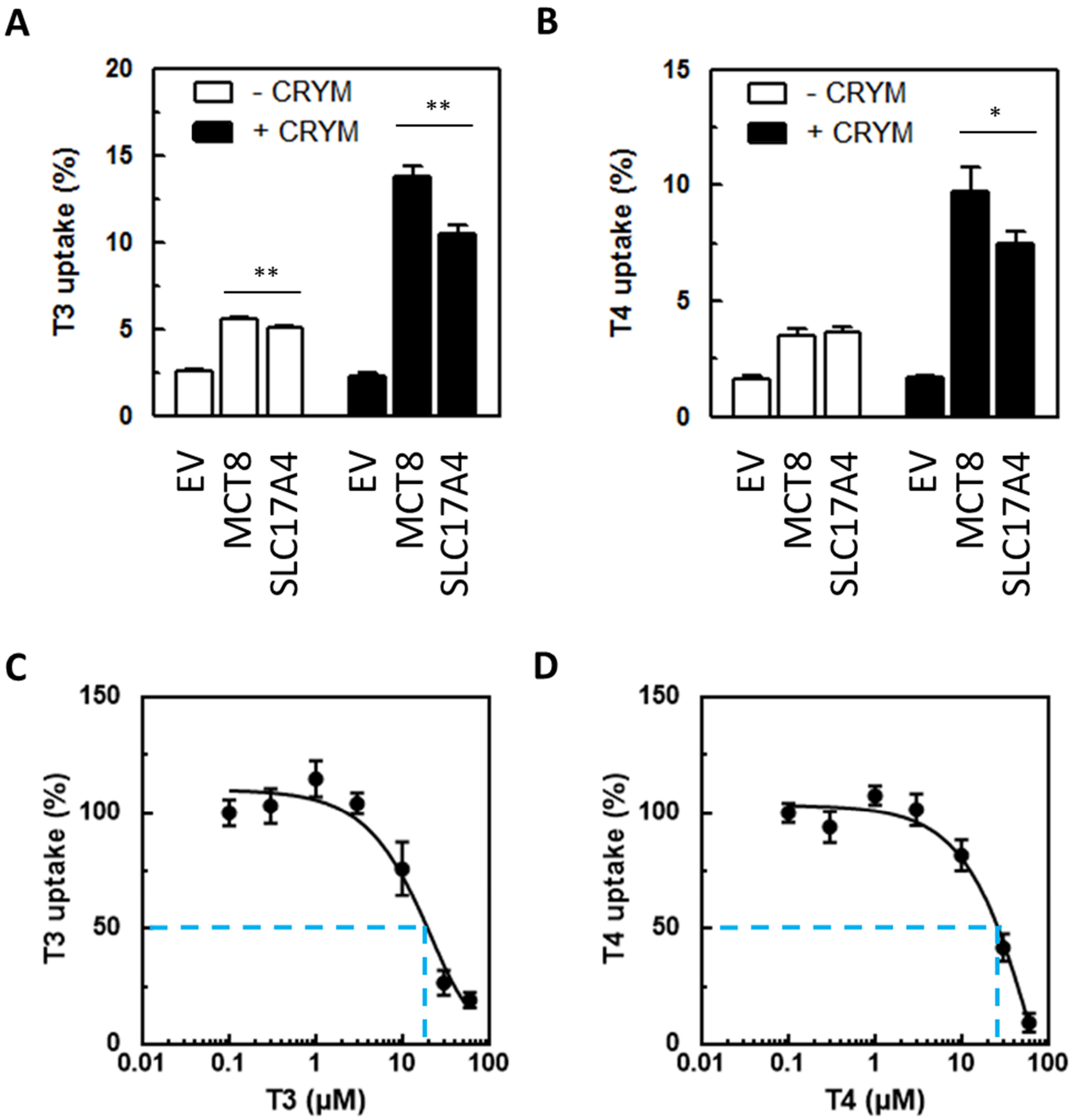

Cellular T3 (Panel A) and T4 (Panel B) accumulation in COS-1 cells, transiently transfected with empty vector (EV), wild-type hMCT8 or wild-type hSLC17A4 in the absence (white bars) or presence (black bars) of the intracellular thyroid hormone binding protein CRYM, after 10 minutes incubation at 37°C. All uptake levels are expressed relative to the amount of radio-labeled T3 or T4 added to the cells at the start of the incubation (1 nM (5x10E4 cpm) [^{125}I]-T3 or [^{125}I]-T4). All results are presented as means ± SEM (n=3). In the presence and absence of CRYM, T3 and T4 accumulation in MCT8 and hSLC17A4 transfected cells was significantly higher compared to empty vector control cells (one-way ANOVA with a Bonferroni corrected post hoc test, P<0.001). In the presence and absence of CRYM, T3 accumulation in hMCT8-transfected cells was slightly, but significantly, higher than in hSLC17A4-transfected cells; which was also the case for T4 uptake in the presence of CRYM. T3 (Panel C) and T4 (Panel D) saturation curves in COS-1 cells transiently transfected with hMCT8 in the absence of CRYM. All data points are corrected for background thyroid hormone uptake in control cells and presented relatively to the amount of internalized thyroid hormone in the presence of the lowest substrate concentration (0.1 µM for T3 and T4). Apparent IC_{50} values were determined by standard second order polynomial regression analyses implemented in GraphPad Prism (La Jolla, USA). * P<0.05; ** P<0.01.

Supplementary Figure 10: QQ plots for all GWAS meta-analyses in the overall samples

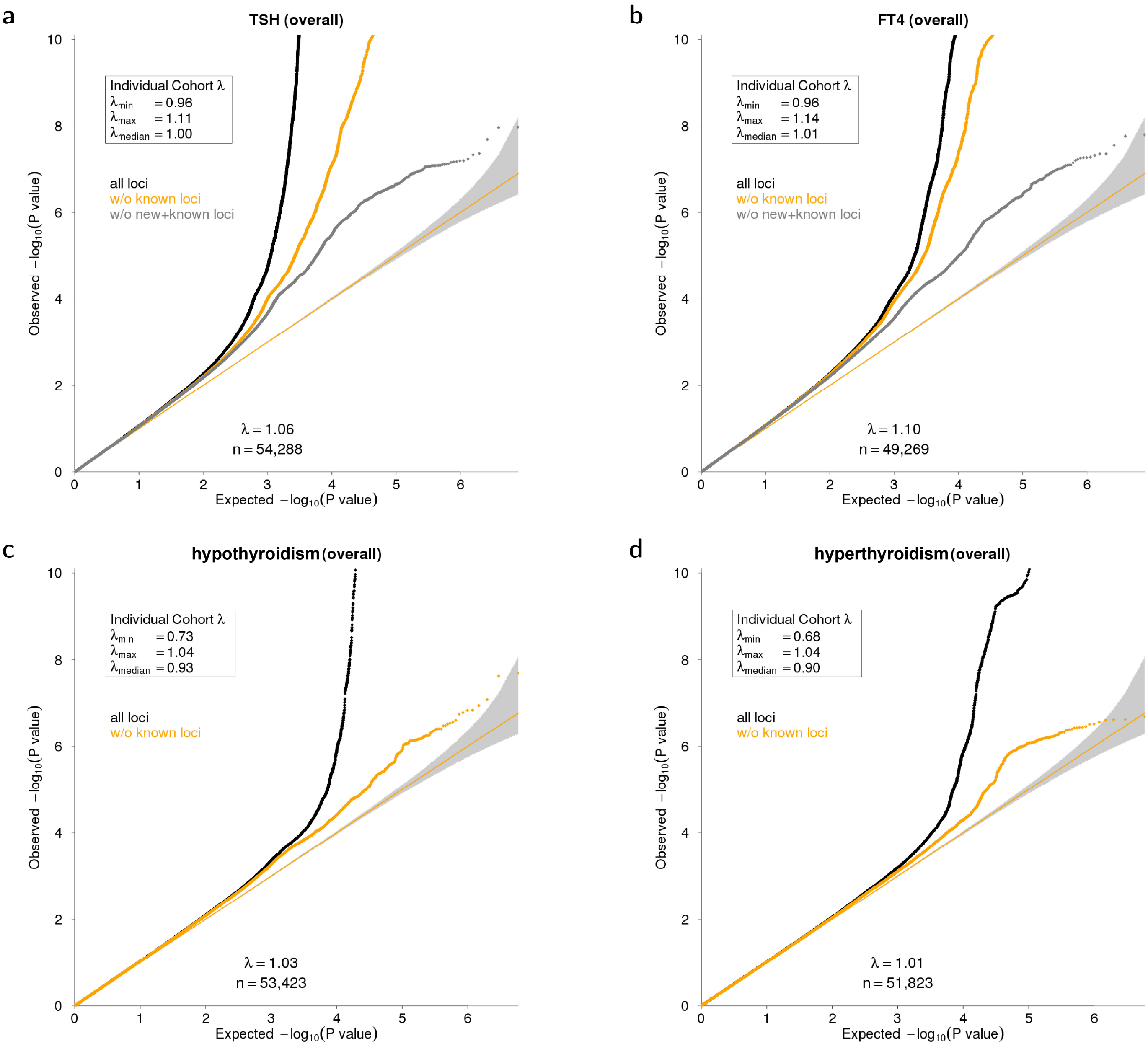

Quantile-quantile (QQ) plots of the GWAS meta-analysis results for (a) TSH, (b) FT4, (c) hypothyroidism, and (d) hyperthyroidism in the overall sample. The observed p-values of the association test are plotted on the y-axis against their expected distribution under the null hypothesis of no association on the x-axis. Results for all SNPs are shown in black, results after removal of loci previously known to contain trait-associated variants are shown in yellow, and results after removal of known and novel loci are shown in gray. Gray bands represent 95% confidence intervals. λ: lambda, genomic control parameter; n: sample size.

Supplementary Figure 11: QQ plots for all GWAS meta-analyses in the sex-stratified samples

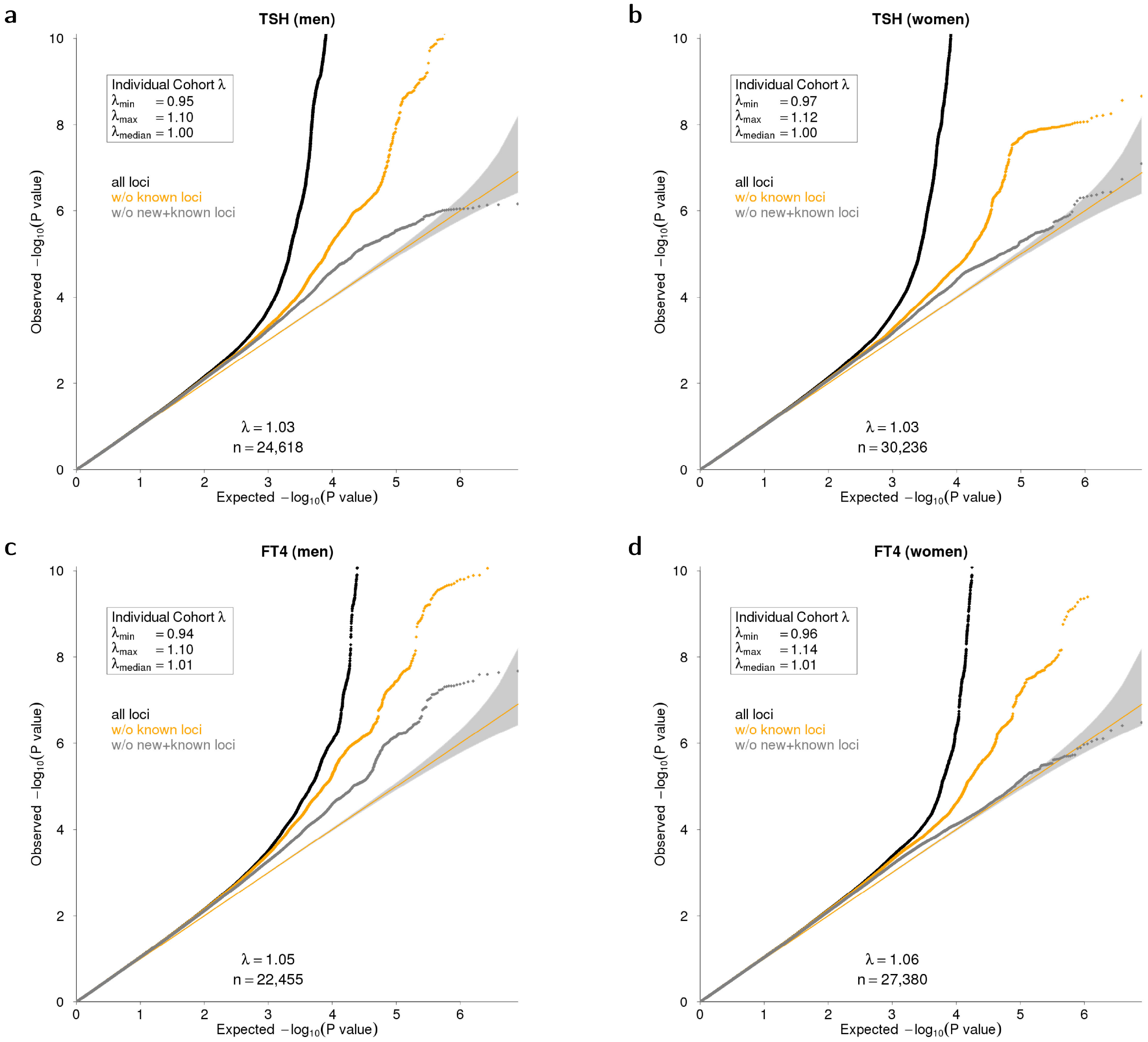

Quantile-quantile (QQ) plots of the GWAS meta-analysis results for TSH in the (a) men and (b) women, and for FT4 in the (c) men and (d) women stratum. The observed p-values of the association test are plotted on the y-axis against their expected distribution under the null hypothesis of no association on the x-axis. Results for all SNPs are shown in black, results after removal of loci previously known to contain trait-associated variants are shown in yellow, and results after removal of known and novel loci are shown in gray. Gray bands represent 95% confidence intervals. λ: lambda, genomic control parameter; n: sample size.

Supplementary Figure 12: Results of pathway analyses

A

Canonical Pathway	TSH	FT4	hyperthyroidism	hypothyroidism
G Protein Signaling Mediated by Tubby	1.21E-05		3.79E-03	
Ephrin Receptor Signaling	1.30E-05			
Cardiac Beta-adrenergic Signaling	3.26E-04		4.47E-03	
Protein Kinase A Signaling	3.33E-04			
Relaxin Signaling	3.49E-04			
Thyronamine and Iodothyronamine Metabolism		1.90E-05		5.89E-03
Thyroid Hormone Metabolism I (via Deiodination)		1.90E-05		5.89E-03
Thyroid Hormone Biosynthesis				5.89E-03
TR/RXR Activation		6.48E-05		
2-amino-3-carboxymuconate Semialdehyde Degradation to Glytaryl-CoA		2.56E-03		
Thyroid Hormone Metabolism II (via conjugation and/or degradation)		3.58E-03		
cAMP-mediated signaling			2.16E-03	
Gustation Pathway			3.43E-03	
tRNA Splicing			3.45E-03	2.16E-03
T Cell Receptor Signaling				9.17E-04

B

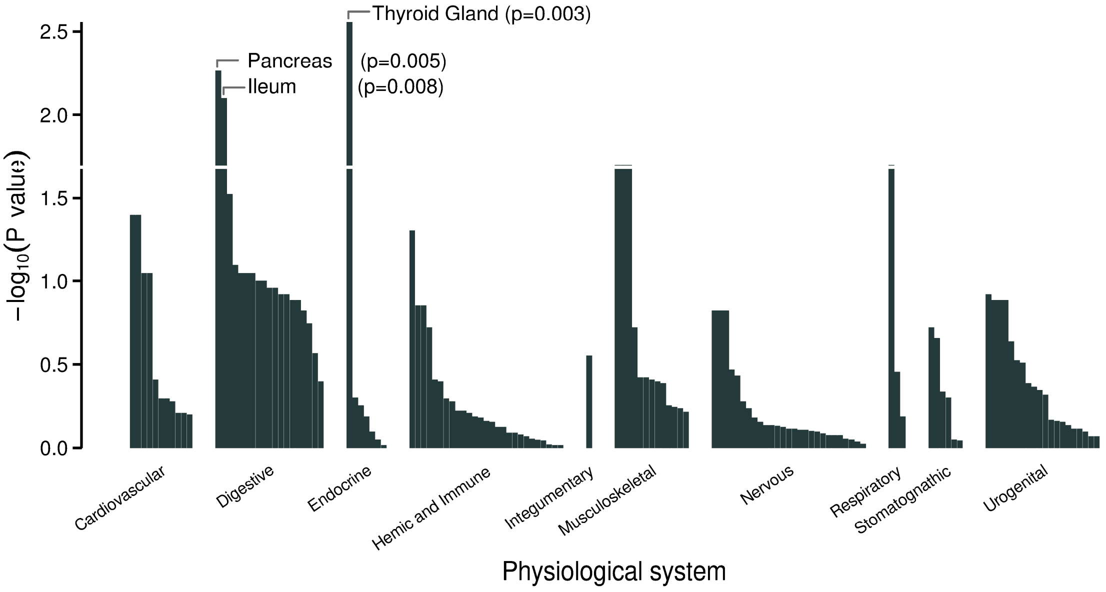

Panel A shows the top canonical pathways of the respective GWAS obtained by the IPA tool. The p-value for each pathway is calculated using a right-tailed Fisher Exact test. Tissue enrichment results based on the TSH GWAS (overall sample) obtained by the DEPICT software are shown in panel B. The nominal p-values <0.01 of the corresponding tissues are provided in the figure.

Supplementary Table 1: Genome-wide significant index SNPs for TSH after final stage

SNP	Chr	Position	Locus	A1	A2	AF1	Effect	SE	P	I2	N total	P GxSex	P hyperthyroidism	P hypothyroidism	P FT4
known loci															
rs12089835*	1	19,771,438	*CAPZB*	t	c	0.35	0.073	0.007	1.3E-28	5.3	51018	0.734	**7.7E-04**	**1.5E-06**	0.015
rs10917469	1	19,843,576	*CAPZB*	a	g	0.84	0.111	0.009	4.0E-39	26.5	51018	0.793	**1.0E-08**	**1.9E-10**	0.001
rs74804879*	1	19,862,320	*CAPZB*	t	c	0.65	0.050	0.007	1.2E-14	5.3	51018	0.287	**6.8E-04**	**3.8E-04**	0.034
rs334725	1	61,610,049	*NFIA*	a	g	0.95	0.174	0.015	2.4E-32	39.2	51018	0.750	**1.6E-04**	**6.7E-05**	**1.1E-04**
rs17020122	1	108,357,391	*VAV3*	t	c	0.09	0.104	0.011	5.3E-20	11.6	51018	0.093	1.1E-01	**6.4E-14**	0.142
rs16856540*	2	217,580,413	*IGFBP5*	t	c	0.84	-0.055	0.008	7.8E-11	0.0	52047	0.805	**6.8E-04**	8.0E-01	0.603
rs13015993	2	217,625,523	*IGFBP5*	a	g	0.73	0.082	0.007	4.5E-32	0.0	52047	0.034	**3.5E-07**	5.4E-02	0.043
rs1663070	3	12,239,852	*SYN2*	t	c	0.74	-0.046	0.007	3.5E-11	0.0	54288	0.243	1.3E-01	3.1E-03	0.889
rs6535624*	4	149,587,905	*NR3C2*	a	g	0.44	0.042	0.006	1.6E-11	0.0	52047	0.441	3.3E-03	2.8E-02	0.387
rs11732089	4	149,665,602	*NR3C2*	t	c	0.80	0.115	0.008	1.7E-51	8.4	52047	0.673	**1.7E-06**	**2.8E-05**	0.563
rs62362610*	5	76,439,961	*PDE8B*	c	g	0.08	0.073	0.012	7.7E-10	0.0	53074	0.205	1.3E-02	7.5E-02	0.156
rs1119208*	5	76,488,613	*PDE8B*	t	c	0.37	0.046	0.006	6.6E-13	13.6	53074	0.835	**4.8E-05**	9.5E-01	0.150
rs139424329*	5	76,495,539	*PDE8B*	a	g	0.01	-0.200	0.032	5.1E-10	0.0	45981	0.640	1.4E-02	5.5E-01	0.574
rs2127387	5	76,532,571	*PDE8B*	a	g	0.41	0.144	0.006	1.1E-117	25.0	53074	0.012	**5.5E-22**	**4.2E-09**	**2.4E-06**
rs7702192*	5	76,554,807	*PDE8B*	a	c	0.47	0.070	0.006	2.6E-30	0.0	53074	0.177	**2.7E-07**	3.6E-03	6.1E-02
rs113974964*	5	76,652,403	*PDE8B*	t	c	0.05	-0.124	0.015	2.1E-17	41.5	53074	0.656	**4.2E-04**	2.7E-01	0.224
rs139149784*	5	76,660,193	*PDE8B*	a	g	0.03	0.156	0.029	5.0E-08	2.3	49018	0.552	1.0E-01	7.9E-03	0.979
rs182873197*	5	76,773,148	*PDE8B*	t	c	0.05	-0.080	0.014	1.7E-08	14.4	53074	0.667	2.5E-01	1.9E-01	0.408
rs744103	6	43,805,362	*VEGFA/LOC100132354*	a	t	0.69	0.092	0.007	6.7E-41	40.9	54288	0.802	**3.7E-05**	**5.7E-05**	**3.3E-05**
rs9381266*	6	43,905,037	*VEGFA/LOC100132354*	t	c	0.74	0.073	0.007	1.8E-25	27.1	54288	0.390	**1.5E-07**	3.3E-03	5.6E-03
rs9497965	6	148,521,292	*SASH1*	t	c	0.40	0.044	0.006	9.8E-13	21.9	54288	0.069	3.5E-01	**1.6E-05**	0.327
rs73022105*	6	165,973,757	*PDE10A*	t	c	0.95	0.105	0.016	1.2E-11	0.0	52424	0.087	2.2E-02	2.4E-01	0.406
rs1079418	6	166,047,034	*PDE10A*	a	g	0.69	0.101	0.007	8.2E-53	20.9	52424	0.206	**3.6E-09**	**4.3E-08**	0.019
rs2439301	8	32,433,013	*NRG1*	a	g	0.23	-0.059	0.008	8.2E-15	13.9	54288	0.177	7.9E-03	3.5E-02	0.308
rs10814915	9	4,290,544	*GLIS3*	t	c	0.44	0.042	0.006	5.1E-12	6.4	54288	0.547	**3.2E-04**	2.5E-03	**5.6E-05**
rs8176645	9	136,149,098	*ABO*	a	t	0.34	0.052	0.006	3.9E-16	51.2	54288	0.383	2.1E-02	**2.2E-04**	0.806
rs200574439	10	101,283,330	*NKX2-3*	a	c	0.58	-0.047	0.006	3.7E-13	13.8	54288	0.311	**3.2E-05**	**1.4E-04**	0.007
rs12284404	11	45,228,686	*PRDM11*	a	g	0.27	-0.067	0.007	2.5E-22	0.0	54288	0.250	**6.0E-11**	9.5E-01	0.414
rs398745	14	36,536,181	*MBIP*	a	c	0.59	-0.052	0.006	4.0E-17	0.0	54288	0.236	1.7E-03	2.2E-02	**1.8E-04**
rs2254613*	14	36,713,154	*MBIP*	t	g	0.55	-0.035	0.006	3.4E-08	27.0	54288	0.754	2.4E-02	4.2E-01	0.008
rs8015085	14	93,585,331	*ITPK1*	a	g	0.21	0.067	0.008	2.4E-18	16.9	54288	0.875	**3.5E-04**	4.5E-03	**2.5E-05**
rs17477923	15	49,711,185	*FAM227B/FGF7*	t	c	0.74	0.083	0.007	2.6E-33	40.3	54288	0.503	**6.1E-13**	1.6E-02	**1.2E-06**
rs11639111*	15	49,749,735	*FAM227B/FGF7*	t	c	0.41	0.045	0.006	3.6E-13	11.0	54288	0.858	9.7E-04	1.3E-01	0.047
rs13329353	15	89,113,877	*DET1*	t	c	0.68	0.061	0.007	5.2E-21	19.2	54288	0.110	3.4E-03	7.9E-01	**3.6E-06**
rs17767491	16	79,745,487	*MAF*	a	g	0.68	0.088	0.007	3.4E-42	40.5	54288	0.069	**7.0E-07**	5.6E-03	0.054
rs1042673	17	70,121,339	*SOX9*	a	g	0.52	-0.055	0.006	3.6E-19	0.0	54288	0.563	**3.2E-06**	8.7E-01	0.672
rs963384*	17	70,369,758	*SOX9*	t	c	0.46	0.035	0.006	2.8E-08	0.0	54288	0.370	**2.7E-08**	2.5E-01	0.643
rs4804413	19	7,222,655	*INSR*	t	c	0.44	0.053	0.006	8.6E-18	0.6	51942	0.865	9.7E-03	1.8E-03	0.030
rs1203944	20	22,596,879	*FOXA2*	t	c	0.23	-0.051	0.007	2.4E-12	0.0	54288	0.040	3.8E-02	**6.2E-05**	0.145
novel loci															
rs6724073	2	218,236,786	*DIRC3*	t	c	0.74	0.051	0.008	1.3E-10	6.8	52047	0.690	1.1E-01	6.3E-02	0.005
rs28502438	3	149,220,109	*TM4SF4*	t	c	0.57	0.034	0.006	3.7E-08	0.0	54288	0.803	7.6E-01	7.2E-02	0.183
rs13100823	3	185,514,088	*IGF2BP2*	t	c	0.31	-0.041	0.007	6.8E-10	9.5	54288	0.367	8.5E-04	**1.7E-04**	0.003
rs59381142	3	193,916,181	*HES1*	a	g	0.24	-0.058	0.008	1.7E-14	0.0	52048	0.225	1.8E-03	2.4E-02	0.353
rs1265091	6	31,108,129	*PSORS1C1*	t	c	0.20	0.057	0.009	3.2E-11	38.5	47651	0.483	3.0E-01	3.0E-02	0.047
rs56009477	8	23,356,964	*SLC25A37*	a	g	0.84	0.052	0.008	3.7E-10	0.0	54288	0.869	9.2E-03	3.5E-02	0.085
rs10957494	8	70,365,025	*SULF1*	a	g	0.69	-0.040	0.007	1.1E-09	24.1	54288	0.008	3.5E-02	2.5E-01	0.221
rs118039499	8	133,771,635	*TG*	a	c	0.98	0.184	0.024	2.0E-14	37.8	48736	0.065	**1.8E-12**	4.0E-01	0.401
rs2739067*	8	133,951,991	*TG*	a	g	0.60	-0.042	0.006	2.4E-11	0.0	54288	0.125	1.2E-01	1.3E-01	0.026
rs9298749	9	16,214,340	*C9orf92*	a	c	0.59	-0.039	0.006	8.8E-10	16.7	54288	0.524	9.0E-01	6.9E-03	0.039
rs11255790	10	8,682,180	*GATA3*	t	c	0.30	-0.041	0.007	6.8E-10	0.0	54288	0.016	1.3E-02	7.9E-01	0.358
rs4933466	10	89,849,519	*PTEN*	a	g	0.60	0.040	0.006	5.1E-10	28.6	54288	0.557	2.6E-01	6.6E-03	0.028
rs4445669	11	115,045,237	*CADM1*	t	c	0.46	-0.040	0.006	5.8E-11	0.0	54288	0.600	5.1E-02	9.4E-02	0.158
rs7329958	13	24,782,080	*SPATA13*	t	c	0.35	-0.044	0.007	1.1E-11	0.0	54288	0.138	6.1E-01	4.5E-03	0.137
rs11159482*	14	81,490,842	*TSHR*	t	c	0.09	0.085	0.013	6.3E-11	0.0	54288	0.397	1.9E-01	**1.8E-04**	0.210
rs59334515*	14	81,594,143	*TSHR*	t	c	0.22	-0.054	0.007	1.1E-13	25.7	54288	0.697	1.4E-02	3.1E-03	0.082
rs12893151	14	81,619,945	*TSHR*	a	c	0.22	-0.062	0.008	1.0E-15	28.2	54288	0.124	**3.2E-04**	**1.9E-04**	0.931
rs1045476	16	4,015,313	*ADCY9*	a	g	0.18	0.049	0.008	2.4E-09	0.0	54288	0.660	1.7E-01	4.6E-03	0.745
rs30227	16	14,405,428	*MIR365A*	t	c	0.61	-0.047	0.006	7.6E-14	11.1	54288	0.955	2.6E-02	1.1E-01	0.087
rs77819282	17	44,762,589	*NSF*	a	g	0.24	0.045	0.007	1.1E-09	0.0	54288	0.419	5.2E-01	7.1E-03	0.715
rs1157994	17	59,338,574	*BCAS3*	a	g	0.05	-0.090	0.016	5.3E-09	25.4	50232	0.990	1.8E-01	4.1E-01	0.003
rs12390237	X	3,612,081	*PRKX*	a	g	0.62	-0.046	0.007	1.7E-11	0.0	36501	0.894	1.0E-02	6.6E-01	0.705

Effects, standard errors (SE), p-values (P) and N total are provided for the discovery stage. (*) additional independent hits of a locus. A1: effect allele; AF1: allele frequency of A1; I2: percentage of total variation across studies that is due to heterogeneity; P GxSex: p-values of gene-by-sex interaction. Bold values of the increased and decreased TSH p-values indicate significance after Bonferroni correction for the 61 independent TSH-associated SNPs tested (p<8.2E-4). Bold values of the FT4 p-values indicate significance after Bonferroni correction for the 91 independent FT4+TSH SNPs tested (p<5.5E-4).

Supplementary Table 2: Genome-wide significant index SNPs for FT4 after final stage

SNP	Chr	Position	Locus	A1	A2	AF1	Effect	SE	P	I2	N total	P GxSex	P TSH
known loci													
rs145019385*	1	54,252,139	*DIO1*	t	c	0.98	0.181	0.032	1.1E-08	0.0	42463	0.799	0.708
rs12033572*	1	54,369,674	*DIO1*	c	g	0.05	0.115	0.017	1.4E-11	0.0	49269	0.371	0.377
rs2235544	1	54,375,570	*DIO1*	a	c	0.52	0.139	0.007	4.2E-101	0.0	49269	0.950	0.062
rs954878*	1	54,578,401	*DIO1*	a	g	0.45	-0.058	0.007	4.8E-19	0.0	49269	0.468	0.799
rs6854291	4	170,992,760	*AADAT*	a	g	0.10	0.117	0.011	1.3E-24	4.5	47314	0.257	0.030
rs10739496	9	100,552,559	*FOXE1*	t	c	0.66	0.078	0.007	4.2E-30	20.1	49269	0.181	**4.8E-07**
rs10984606*	9	100,739,117	*FOXE1*	t	g	0.50	-0.040	0.007	1.2E-09	0.0	49269	0.405	0.030
rs4842131	9	139,092,679	*LHX3*	t	c	0.45	-0.104	0.008	7.7E-44	4.4	44811	0.285	0.315
rs55679545*	9	139,122,363	*LHX3*	a	g	0.27	0.044	0.008	8.4E-09	0.0	49269	0.428	0.321
rs1080094*	18	29,173,795	*SLC25A52*	a	g	0.60	-0.042	0.007	4.1E-10	14.5	49269	0.008	0.272
rs113107469	18	29,306,737	*SLC25A52*	t	c	0.03	0.200	0.022	1.0E-19	34.3	49269	0.431	0.510
novel loci													
rs4954192	2	135,632,981	*ACMSD*	t	c	0.43	-0.041	0.007	8.4E-09	0.0	44902	0.826	0.333
rs6785807	3	181,718,601	*SOX2-OT*	a	g	0.15	-0.059	0.009	2.5E-10	14.4	49269	0.026	0.297
rs10946313	6	19,381,386	*ID4*	t	c	0.63	0.046	0.007	2.3E-11	0.0	49269	0.243	0.002
rs9356988	6	25,777,481	*SLC17A4*	a	g	0.27	-0.051	0.007	3.6E-12	0.0	49269	0.273	0.802
rs137964359*	6	26,001,742	*SLC17A4*	t	c	0.99	-0.200	0.032	2.1E-10	0.0	49269	0.175	0.335
rs17185536	6	100,620,931	*LOC728012*	t	c	0.24	0.073	0.008	1.9E-19	0.0	49269	0.368	0.707
rs67583169	8	61,212,179	*CA8*	c	g	0.87	0.061	0.010	1.0E-10	0.0	49269	0.718	0.169
rs10119187	9	4,223,660	*GLIS3*	t	c	0.81	0.050	0.009	4.1E-09	15.9	49269	0.933	**2.2E-06**
rs10818937	9	127,015,440	*NEK6*	t	c	0.31	-0.048	0.007	1.3E-11	0.0	49269	0.388	6.3E-04
rs11039355	11	47,737,501	*FNBP4*	t	c	0.34	-0.039	0.007	3.5E-08	20.9	49269	0.080	0.764
rs4149056	12	21,331,549	*SLCO1B1*	t	c	0.84	-0.051	0.009	1.3E-08	5.2	49269	0.432	0.094
rs150816132*	14	80,464,293	*DIO2*	a	g	0.01	-0.220	0.040	3.5E-08	24.1	38640	0.453	0.435
rs978055*	14	80,534,869	*DIO2*	a	t	0.38	0.038	0.007	1.1E-08	10.5	49269	0.350	0.642
rs225014	14	80,669,580	*DIO2*	t	c	0.64	0.054	0.007	1.8E-15	0.0	49269	0.759	0.006
rs12323871*	14	101,852,075	*DIO3OS*	t	c	0.82	-0.047	0.008	1.4E-08	25.7	49269	0.829	0.144
rs11626434	14	101,998,443	*DIO3OS*	c	g	0.36	0.058	0.007	4.1E-17	40.7	49269	0.908	0.340
rs12907106	15	63,873,658	*USP3*	c	g	0.27	-0.041	0.007	3.7E-08	0.7	49269	0.038	0.494
rs8063103	16	12,703,395	*SNX29*	c	g	0.85	-0.052	0.009	1.6E-08	10.4	49269	0.064	0.934
rs11078333	17	16,049,626	*NCOR1*	a	t	0.53	0.051	0.007	9.9E-13	0.0	49269	0.956	0.746
rs56069042	18	57,914,644	*MC4R*	a	g	0.96	0.106	0.019	1.2E-08	0.0	49269	0.516	0.499

Effects, standard errors (SE), p-values (P) and N total are provided for the discovery stage. (*) additional independent hits of a locus. A1: effect allele; AF1: allele frequency of A1; I2: percentage of total variation across studies that is due to heterogeneity; P GxSex: p-values of gene-by-sex interaction. Bold values of the TSH p-values indicate significance after Bonferroni correction for the 91 independent FT4+TSH SNPs tested ($p<5.5E-4$).

Supplementary Table 3: Distribution of sex and age group for hypo- and hyperthyroidism GWAS cases and controls

hypothyroidism				hyperthyroidism			
age	sex	cases	controls	age	sex	cases	controls
0-19	male	0.0%	0.7%	0-19	male	0.0%	0.7%
0-19	female	0.0%	0.8%	0-19	female	0.0%	0.8%
20-39	male	0.2%	6.9%	20-39	male	0.2%	7.1%
20-39	female	0.4%	7.7%	20-39	female	0.3%	8.0%
40-59	male	0.6%	17.5%	40-59	male	0.6%	18.0%
40-59	female	1.3%	17.7%	40-59	female	0.7%	18.2%
60-79	male	1.0%	20.0%	60-79	male	0.7%	20.6%
60-79	female	2.1%	18.6%	60-79	female	0.8%	19.2%
80+	male	0.2%	1.5%	80+	male	0.1%	1.6%
80+	female	0.3%	2.3%	80+	female	0.1%	2.4%

The % values correspond to the total number of samples in the hypo- and hyperthyroidism analysis, respectively.

Supplementary Table 4: Genome-wide significant index SNPs for hypo- and hyperthyroidism

SNP	Chr	Position	Locus	A1	A2	AF1	Effect	SE	P	I2	N total	P TSH	Reference
hypothyroidism													
rs7032019*	9	100,548,144	*FOXE1*	a	g	0.66	0.248	0.027	5.5E-20	11.8	53241	0.000001229	1, 2
rs78495697	1	108,355,719	*VAV3*	t	c	0.09	0.316	0.041	2.6E-14	12.6	53241	**9.278E-20**	2
rs597808	12	111,973,358	*ATXN2/SH2B3*	a	g	0.51	0.180	0.026	3.5E-12	21.9	53241	2.29E-06	2
rs75491569	1	19,836,221	*CAPZB*	t	c	0.16	-0.238	0.037	8.7E-11	0.0	53241	**7.805E-38**	1
rs11675342	2	1,407,628	*TPO*	t	c	0.43	0.160	0.025	1.5E-10	0.0	53241	7.88E-06	3
rs1382879	5	76,521,868	*PDE8B*	t	c	0.59	-0.153	0.026	2.1E-09	0.0	48969	**1.51E-115**	1
rs2983514	6	166,050,119	*PDE10A*	a	g	0.67	0.154	0.027	1.6E-08	15.5	51313	**4.745E-48**	1
rs12449792**	17	43,302,259	*FMNL1*	t	c	0.46	0.157	0.028	2.4E-08	0.0	46314	**4.619E-08**	
hyperthyroidism													
SNP	Chr	Position	Locus	A1	A2	AF1	Effect	SE	P	I2	N total	P TSH	Reference
rs2046045	5	76,535,811	*PDE8B*	t	g	0.60	0.329	0.034	4.4E-22	57.3	50420	**3.72E-117**	1
rs17477923	15	49,711,185	*FAM227B/FGF7*	t	c	0.72	-0.245	0.034	6.1E-13	28.7	48508	**2.572E-33**	1
rs11038357	11	45,237,858	*PRDM11*	a	t	0.28	0.235	0.035	3.1E-11	42.1	48508	**3.244E-22**	1
rs8077245	17	70,376,177	*SOX9*	t	g	0.58	0.205	0.033	5.0E-10	0.0	48508	**6.231E-14**	1
rs2983514	6	166,050,119	*PDE10A*	a	g	0.65	-0.199	0.032	5.2E-10	37.7	49745	**4.745E-48**	1
rs12138950	1	19,839,115	*CAPZB*	a	c	0.85	-0.258	0.042	5.5E-10	64.3	51668	**5.789E-38**	1
rs925488*	9	100,546,391	*FOXE1*	a	g	0.65	0.190	0.033	1.1E-08	66.0	48508	6.482E-07	1, 2, 3
rs66760320	6	43,906,255	*VEGFA/LOC100132354*	t	c	0.27	0.192	0.035	4.4E-08	22.6	51668	**1.011E-24**	3

(*) FT4 locus (p=3E-29). (**) discovered also in TSH, but did not replicate. SE: standard erros; A1: effect allele; AF1: allele frequency of A1; I2: percentage of total variation across studies that is due to heterogeneity. Bold values of TSH indicate genome-wide significance.
References: 1) Porcu et al. 2012 2) Eriksson et al. 2012 3) Gudmundsson et al. 2012

Supplementary Table 5: Lookup of TSH associated SNPs in Graves' disease and TPOab positivity

Graves' disease							
SNP	Chr	Position	Nearest gene	A1	A2	Effect direction	P
rs12089835	1	19,771,438	*CAPZB*	t	c	-	0.090
rs10917469	1	19,843,576	*CAPZB*	a	g	-	0.005
rs74804879	1	19,862,320	*CAPZB*	t	c	-	0.823
rs334725	1	61,610,049	*NFIA*	a	g	+	0.206
rs17020122	1	108,357,391	*VAV3*	t	c	-	0.112
rs16856540	2	217,580,413	*IGFBP5*	t	c	+	0.021
rs13015993	2	217,625,523	*IGFBP5*	a	g	-	0.008
rs6724073	2	218,236,786	*DIRC3*	t	c	+	0.503
rs1663070	3	12,239,852	*SYN2*	t	c	-	0.035
rs28502438	3	149,220,109	*TM4SF4*	t	c	-	0.423
rs13100823	3	185,514,088	*IGF2BP2*	t	c	+	0.018
rs59381142	3	193,916,181	*HES1*	a	g	-	0.924
rs6535624	4	149,587,905	*NR3C2*	a	g	-	0.736
rs11732089	4	149,665,602	*NR3C2*	t	c	-	0.934
rs1119208	5	76,488,613	*PDE8B*	t	c	+	0.407
rs2127387	5	76,532,571	*PDE8B*	a	g	-	0.192
rs7702192	5	76,554,807	*PDE8B*	a	c	-	0.599
rs1265091	6	31,108,129	*PSORS1C1*	t	c	-	**1.3E-12**
rs744103	6	43,805,362	*VEGFA*	a	t	+	0.332
rs9381266	6	43,905,037	*LOC100132354*	t	c	-	0.290
rs9497965	6	148,521,292	*SASH1*	t	c	+	0.048
rs73022105	6	165,973,757	*PDE10A*	t	c	-	0.707
rs1079418	6	166,047,034	*PDE10A*	a	g	+	0.854
rs56009477	8	23,356,964	*SLC25A37*	a	g	-	0.269
rs2439301	8	32,433,013	*NRG1*	a	g	+	0.321
rs10957494	8	70,365,025	*SULF1*	a	g	-	0.287
rs2739067	8	133,951,991	*TG*	a	g	-	0.148
rs10814915	9	4,290,544	*GLIS3*	t	c	+	0.284
rs9298749	9	16,214,340	*C9orf92*	a	c	+	0.377
rs8176645	9	136,149,098	*ABO*	a	t	-	0.722
rs11255790	10	8,682,180	*GATA3*	t	c	+	0.110
rs4933466	10	89,849,519	*PTEN*	a	g	+	0.420
rs12284404	11	45,228,686	*PRDM11*	a	g	+	0.986
rs4445669	11	115,045,237	*CADM1*	t	c	+	0.055
rs7329958	13	24,782,080	*SPATA13*	t	c	-	0.223
rs398745	14	36,536,181	*LINC00609*	a	c	+	0.350
rs2254613	14	36,713,154	*MBIP*	t	g	+	0.570
rs11159482	14	81,490,842	*TSHR*	t	c	+	0.215
rs59334515	14	81,594,143	*TSHR*	t	c	+	0.017
rs12893151	14	81,619,945	*TSHR*	a	c	-	0.193
rs8015085	14	93,585,331	*ITPK1*	a	g	+	0.912
rs17477923	15	49,711,185	*FAM227B*	t	c	+	0.764
rs11639111	15	49,749,735	*FAM227B*	t	c	-	0.089
rs13329353	15	89,113,877	*DET1*	t	c	-	0.795
rs1045476	16	4,015,313	*ADCY9*	a	g	+	0.344
rs30227	16	14,405,428	*MIR365A*	t	c	+	0.966
rs17767491	16	79,745,487	*MAF*	a	g	-	0.001
rs77819282	17	44,762,589	*NSF*	a	g	-	0.867
rs1042673	17	70,121,339	*SOX9*	a	g	+	0.651
rs963384	17	70,369,758	*LINC00673*	t	c	+	0.647
rs4804413	19	7,222,655	*INSR*	t	c	-	0.230
rs1203944	20	22,596,879	*FOXA2*	t	c	+	0.060

TPOab positivity							
SNP	Chr	Position	Nearest gene	A1	A2	Effect direction	P
rs12091047	1	19,771,925	CAPZB	t	c	-	0.187
rs10917469	1	19,843,576	CAPZB	a	g	-	0.836
rs334725	1	61,610,049	NFIA	a	g	-	0.788
rs17020122	1	108,357,391	VAV3	t	c	+	**7.4E-04**
rs16856540	2	217,580,413	IGFBP5	t	c	+	0.313
rs13015993	2	217,625,523	IGFBP5	a	g	-	0.052
rs6724073	2	218,236,786	TNS1	t	c	-	0.003
rs1663070	3	12,239,852	SYN2	t	c	+	0.821
rs9828967	3	149,226,442	TM4SF4	a	t	+	0.319
rs6767484	3	185,520,578	IGF2BP2	a	g	+	0.916
rs4578973	3	193,918,634	HES1	t	c	+	0.524
rs6535624	4	149,587,905	NR3C2	a	g	+	0.017
rs11732089	4	149,665,602	NR3C2	t	c	+	0.089
rs1119208	5	76,488,613	PDE8B	t	c	-	0.957
rs6885099	5	76,530,349	PDE8B	a	g	-	0.736
rs7702192	5	76,554,807	PDE8B	a	c	+	0.219
rs12189200	5	76,658,640	PDE8B	t	c	-	0.080
rs6869902	5	76,783,252	WDR41	a	c	-	0.810
rs1063646	6	31,107,648	PSORS1C1	t	c	+	0.528
rs744103	6	43,805,362	VEGFA	a	t	-	0.991
rs9381266	6	43,905,037	C6orf223	t	c	-	0.460
rs9497965	6	148,521,292	SASH1	t	c	+	0.122
rs4709949	6	165,966,099	PDE10A	t	c	-	0.447
rs1079418	6	166,047,034	PDE10A	a	g	-	0.128
rs17770799	8	23,347,851	ENTPD4	t	c	+	0.766
rs2439300	8	32,433,104	NRG1	t	c	+	0.115
rs7011706	8	70,362,479	SULF1	t	c	-	0.077
rs2739068	8	133,952,435	TG	t	g	-	0.055
rs10758594	9	4,295,583	GLIS3	a	g	+	0.447
rs9298749	9	16,214,340	BNC2	a	c	-	0.385
rs644234	9	136,142,217	ABO	t	g	+	0.069
rs11255790	10	8,682,180	GATA3	t	c	-	0.287
rs4933466	10	89,849,519	PTEN	a	g	+	0.989
rs7077040	10	101,281,014	NKX2-3	c	g	-	0.286
rs17723470	11	45,227,567	PRDM11	t	c	+	0.112
rs4445669	11	115,045,237	CADM1	t	c	+	0.849
rs4770628	13	24,782,064	SPATA13	a	g	-	**1.1E-04**
rs398745	14	36,536,181	BRMS1L	a	c	+	0.291
rs2254613	14	36,713,154	MBIP	t	g	+	0.656
rs2268476	14	81,592,717	TSHR	a	g	+	0.192
rs9672082	14	93,572,855	ITPK1	t	c	+	0.114
rs17477923	15	49,711,185	C15orf33	t	c	-	0.013
rs11639111	15	49,749,735	C15orf33	t	c	-	0.095
rs13329353	15	89,113,877	DET1	t	c	+	0.338
rs2072346	16	4,027,423	ADCY9	t	c	+	0.930
rs30227	16	14,405,428	MKL2	t	c	+	0.932
rs17767491	16	79,745,487	MAF	a	g	-	**2.2E-05**
rs183211	17	44,788,310	NSF	a	g	+	0.963
rs17597856	17	59,314,176	BCAS3	c	g	-	0.215
rs9915657	17	70,127,536	SOX9	t	c	+	0.283
rs759563	17	70,370,888	SOX9	t	c	+	0.287
rs4804416	19	7,223,848	INSR	t	g	+	0.100
rs1203944	20	22,596,879	FOXA2	t	c	+	0.623

A1: effect allele. Bold values indicate significance ($p<0.05$) after Bonferroni correction for the number of SNPs per trait tested.

Supplementary Table 6: Association of *SLC17A4* and *AADAT* hits with T3 and T3/T4 ratio

SNP	Locus	A1	A2	Effect	SE	P	I^2	N total	N cohorts
FT3									
rs9356988	SLC17A4	a	g	0.022	0.015	0.143	0	10803	3
rs137964359	SLC17A4	t	c	0.012	0.062	0.849	23.5	10803	3
rs6854291	AADAT	a	g	-0.068	0.023	*0.003*	0	10803	3
TT3									
rs9356988	SLC17A4	a	g	0.019	0.016	0.238	0	9083	2
rs137964359	SLC17A4	t	c	-0.114	0.074	0.125	0	9083	2
rs6854291	AADAT	a	g	-0.049	0.025	0.047	64.2	9083	2
T3*									
rs9356988	SLC17A4	a	g	0.021	0.011	0.06	0	19886	5
rs137964359	SLC17A4	t	c	-0.04	0.048	0.401	14.4	19886	5
rs6854291	AADAT	a	g	-0.059	0.017	***4.46E-04***	10.6	19886	5
FT3/FT4 ratio									
rs9356988	SLC17A4	a	g	0.008	0.003	*0.004*	54.9	10800	3
rs137964359	SLC17A4	t	c	0.016	0.012	0.204	0	10800	3
rs6854291	AADAT	a	g	-0.028	0.004	***1.15E-10***	3.8	10800	3
TT3/FT4 ratio									
rs9356988	SLC17A4	a	g	0.009	0.003	*0.007*	0	9082	2
rs137964359	SLC17A4	t	c	0.019	0.016	0.221	55.6	9082	2
rs6854291	AADAT	a	g	-0.018	0.005	***7.77E-04***	0	9082	2
T3/FT4 ratio*									
rs9356988	SLC17A4	a	g	0.009	0.002	***8.42E-05***	11.4	19882	5
rs137964359	SLC17A4	t	c	0.017	0.01	0.079	0	19882	5
rs6854291	AADAT	a	g	-0.024	0.003	***1.24E-12***	25.5	19882	5

SE: standard erros; A1: effect allele; AF1: allele frequency of A1. Bold p values of TSH indicate statistical significance ($p<0.05$) after Bonferroni correction. FT3: free T3; TT3: total T3;
* indicates a combined analysis of FT3 and TT3 of disjunct samples.

Supplementary Table 7: GRS association with hypo- and hyperthyroidism

GRS	outcome:quantile	Effect	SE	p	OR
TSH	hypothyroidism:1	reference			
TSH	hypothyroidism:2	0.272	0.087	1.82E-03	1.31
TSH	hypothyroidism:3	0.566	0.084	1.26E-11	1.76
TSH	hypothyroidism:4	0.929	0.079	6.76E-32	2.53
TSH	hyperthyroidism:1	reference			
TSH	hyperthyroidism:2	-0.746	0.101	1.34E-13	0.47
TSH	hyperthyroidism:3	-1.090	0.112	1.45E-22	0.34
TSH	hyperthyroidism:4	-1.649	0.143	9.81E-31	0.19
FT4	hypothyroidism:1	reference			
FT4	hypothyroidism:2	-0.034	0.075	0.649	0.97
FT4	hypothyroidism:3	0.041	0.074	0.580	1.04
FT4	hypothyroidism:4	0.034	0.074	0.642	1.03
FT4	hyperthyroidism:1	reference			
FT4	hyperthyroidism:2	-0.064	0.114	0.575	0.94
FT4	hyperthyroidism:3	-0.064	0.114	0.575	0.94
FT4	hyperthyroidism:4	0.035	0.112	0.752	1.04
TSH	hypothyroidism:Genescore	0.100	0.007	4.47E-45	1.11
TSH	hyperthyroidism:Genescore	-0.179	0.011	7.90E-60	0.84
FT4	hypothyroidism:Genescore	0.004	0.006	0.535	1.00
FT4	hyperthyroidism:Genescore	0.004	0.009	0.642	1.00

Association results of the genetic risk score (GRS) based on TSH and FT4 with hypo- and hyperthyroidism contrasting the respective quartile of the GRS distribution against the lowest quartile (1). Genescore corresponds to the association results on the continuous GRS scale (0-100). SE: standard error; OR: odds ratio

Supplementary Table 8: Association results of TSH-based genetic risk score

Trait	#SNPs in GRS	Effect (SE)	P	FDR	Reference
Graves' disease	52	-0.443 (0.104)	2.00E-05	**8.50E-05**	
hypothyroidism	61	1.203 (0.060)	6.20E-89	**1.10E-87**	
hyperthyroidism	61	-1.957 (0.074)	4.00E-156	**1.40E-154**	
TPO antibody (case/control)	53	-0.137 (0.081)	8.90E-02	2.00E-01	Medici et al. (2014)
goiter	53	-1.190 (0.110)	3.90E-27	**3.40E-26**	Teumer et al. (2011)
thyroid volume	53	-0.262 (0.020)	1.30E-37	**1.60E-36**	Teumer et al. (2011)
anxiety (case/control)	53	-0.116 (0.071)	1.00E-01	2.10E-01	Otowa et al. (2016)
anxiety (factor score)	52	0.002 (0.013)	8.80E-01	9.30E-01	Otowa et al. (2016)
type 2 diabetes (DIAGRAM)	50	-0.110 (0.045)	1.60E-02	5.00E-02	Morris et al. (2012)
forearm BMD (GEFOS)	59	-0.051 (0.037)	1.70E-01	3.10E-01	Zheng et al. (2015)
femoral neck BMD (GEFOS)	60	-0.010 (0.018)	5.70E-01	7.40E-01	Zheng et al. (2015)
lumbar spine BMD (GEFOS)	60	-0.004 (0.021)	8.50E-01	9.30E-01	Zheng et al. (2015)
body mass index (GIANT)	51	0.006 (0.009)	5.00E-01	7.20E-01	Locke et al. (2015)
waist circumference adjBMI (GIANT)	51	-0.022 (0.010)	3.30E-02	8.80E-02	Shungin et al. (2015)
waist-hip-ratio adjBMI (GIANT)	51	4.8E-04 (0.010)	9.60E-01	9.80E-01	Shungin et al. (2015)
body height (GIANT)	51	-0.050 (0.007)	2.00E-11	**1.40E-10**	Wood et al. (2014)
obesity class 1 (GIANT)	50	0.026 (0.032)	4.20E-01	6.30E-01	Berndt et al. (2013)
obesity class 2 (GIANT)	50	0.032 (0.049)	5.10E-01	7.20E-01	Berndt et al. (2013)
obesity class 3 (GIANT)	50	0.046 (0.092)	6.20E-01	7.70E-01	Berndt et al. (2013)
gout (GUGC)	50	0.149 (0.078)	5.50E-02	1.40E-01	Köttgen et al. (2013)
uric acid (GUGC)	50	0.021 (0.014)	1.20E-01	2.40E-01	Köttgen et al. (2013)
IGF-I	50	-4.2E-04 (0.022)	9.80E-01	9.80E-01	Teumer et al. (2016)
IGFBP3	50	-0.045 (0.026)	8.00E-02	1.90E-01	Teumer et al. (2016)
HDL (GLGC)	48	0.011 (0.012)	3.60E-01	6.00E-01	Willer et al. (2013)
LDL (GLGC)	48	0.035 (0.013)	4.90E-03	**1.90E-02**	Willer et al. (2013)
total cholesterol (GLGC)	48	0.054 (0.012)	1.10E-05	**6.50E-05**	Willer et al. (2013)
triglyceride (GLGC)	48	-0.004 (0.011)	7.50E-01	9.00E-01	Willer et al. (2013)
fasting glucose (MAGIC)	50	0.005 (0.009)	5.70E-01	7.40E-01	Dupuis et al. (2010)
HbA1C (MAGIC)	52	0.010 (0.009)	2.40E-01	4.20E-01	Soranzo et al. (2010)
bipolar disorder (PGC)	49	-0.050 (0.060)	4.10E-01	6.30E-01	PGC BD WG (2011)
major depressive disorder (PGC)	34	-0.011 (0.068)	8.70E-01	9.30E-01	PGC MDD WG (2011)
kidney function eGFR (CKDGen)	48	-0.010 (0.002)	1.40E-05	**7.20E-05**	Pattaro et al. (2016)
kidney function UACR (CKDGen)	48	-0.035 (0.016)	3.00E-02	8.80E-02	Teumer et al. (2015)
schizophrenia (PGC)	61	-0.064 (0.025)	1.10E-02	**3.80E-02**	Schizophrenia PGC (2011)
education years (SSGAC)	49	-0.009	8.10E-01	9.30E-01	Rietveld et al. (2013)

Bold values indicate significant associations of GRS with the respective trait (false discovery rate < 0.05). Effect provides the change of the unit of the corresponding trait (or log of odd ratio for binary traits) per increase in standard deviation of TSH.

Supplementary Table 9: Association results of FT4-based genetic risk score

Trait	#SNPs in GRS	Effect (SE)	P	FDR	Reference
Graves' disease	25	-0.225 (0.151)	1.37E-01	3.49E-01	
hypothyroidism	31	0.078 (0.084)	3.52E-01	5.60E-01	
hyperthyroidism	31	0.179 (0.105)	8.79E-02	3.42E-01	
goiter	27	0.417 (0.157)	7.90E-03	**4.61E-02**	Teumer et al. (2011)
thyroid volume	27	0.084 (0.029)	3.84E-03	**3.36E-02**	Teumer et al. (2011)
TPO antibody (case/control)	27	0.004 (0.113)	9.72E-01	9.72E-01	Medici et al. (2014)
anxiety (case/control)	27	-0.238 (0.099)	1.64E-02	8.19E-02	Otowa et al. (2016)
anxiety (factor score)	27	-0.012 (0.018)	4.78E-01	6.69E-01	Otowa et al. (2016)
type 2 diabetes (DIAGRAM)	27	-0.056 (0.065)	3.90E-01	5.69E-01	Morris et al. (2012)
forearm BMD (GEFOS)	30	0.055 (0.053)	2.98E-01	5.48E-01	Zheng et al. (2015)
femoral neck BMD (GEFOS)	31	0.012 (0.025)	6.37E-01	7.50E-01	Zheng et al. (2015)
lumbar spine BMD (GEFOS)	31	0.044 (0.029)	1.29E-01	3.49E-01	Zheng et al. (2015)
body mass index (GIANT)	27	-0.039 (0.013)	2.74E-03	**3.19E-02**	Locke et al. (2015)
waist circumference adjBMI (GIANT)	27	-0.001 (0.015)	9.42E-01	9.72E-01	Shungin et al. (2015)
waist-hip-ratio adjBMI (GIANT)	27	-0.019 (0.015)	1.94E-01	4.54E-01	Shungin et al. (2015)
body height (GIANT)	27	0.039 (0.011)	2.92E-04	**5.50E-03**	Wood et al. (2014)
obesity class 1 (GIANT)	27	-0.044 (0.045)	3.31E-01	5.51E-01	Berndt et al. (2013)
obesity class 2 (GIANT)	27	-0.014 (0.069)	8.45E-01	9.24E-01	Berndt et al. (2013)
obesity class 3 (GIANT)	27	0.008 (0.126)	9.50E-01	9.72E-01	Berndt et al. (2013)
gout (GUGC)	27	0.173 (0.117)	1.40E-01	3.49E-01	Köttgen et al. (2013)
uric acid (GUGC)	27	-0.029 (0.020)	1.32E-01	3.49E-01	Köttgen et al. (2013)
IGF-I	27	0.036 (0.031)	2.41E-01	4.68E-01	Teumer et al. (2016)
IGFBP3	27	0.008 (0.037)	8.19E-01	9.24E-01	Teumer et al. (2016)
HDL (GLGC)	27	0.020 (0.016)	2.17E-01	4.68E-01	Willer et al. (2013)
LDL (GLGC)	27	-0.063 (0.017)	3.14E-04	**5.50E-03**	Willer et al. (2013)
total cholesterol (GLGC)	27	-0.047 (0.017)	6.07E-03	**4.25E-02**	Willer et al. (2013)
triglyceride (GLGC)	27	-0.010 (0.016)	5.32E-01	7.16E-01	Willer et al. (2013)
fasting glucose (MAGIC)	27	-0.013 (0.013)	3.27E-01	5.51E-01	Dupuis et al. (2010)
HbA1C (MAGIC)	27	0.015 (0.012)	2.32E-01	4.68E-01	Soranzo et al. (2010)
bipolar disorder (PGC)	27	-0.162 (0.085)	5.57E-02	2.44E-01	PGC BD WG (2011)
major depressive disorder (PGC)	10	-0.086 (0.098)	3.78E-01	5.69E-01	PGC MDD WG (2011)
kidney function eGFR (CKDGen)	25	0.002 (0.003)	6.11E-01	7.50E-01	Pattaro et al. (2016)
kidney function UACR (CKDGen)	26	-0.034 (0.023)	1.36E-01	3.49E-01	Teumer et al. (2015)
schizophrenia (PGC)	31	-0.020 (0.035)	5.68E-01	7.37E-01	Schizophrenia PGC (2011)
education years (SSGAC)	27	-0.006 (0.013)	6.43E-01	7.50E-01	Rietveld et al. (2013)

Bold values indicate significant associations of GRS with the respective trait (false discovery rate < 0.05). Effect provides the change of the unit of the corresponding trait (or log of odd ratio for binary traits) per increase in standard deviation of FT4.

Supplementary Table 10: Replication results of GWAS SNPs

SNP	Chr	Position	Locus	A1	A2	AF1	Effect discovery	P discovery	Effect replication	P replication	N replication	P combined	N combined	proxy SNP (R2)
TSH														
rs12893151	14	81,619,945	*TSHR*	a	c	0.22	-0.062	1.0E-15	-0.025	1.7E-01	9011	2.3E-15	63299	
rs59381142	3	193,916,181	*HES1*	a	g	0.24	-0.058	1.7E-14	-0.036	3.5E-02	9011	3.6E-15	61059	
rs118039499	8	133,771,635	*TG*	a	c	0.98	0.184	2.0E-14	0.188	2.5E-08	17879	2.9E-21	66615	rs114322847 (0.87)
rs30227	16	14,405,428	*MIR365A*	t	c	0.61	-0.047	7.6E-14	-0.042	6.1E-05	17879	2.3E-17	72167	rs30229 (0.91)
rs7329958	13	24,782,080	*SPATA13*	t	c	0.35	-0.044	1.1E-11	-0.047	1.6E-03	9011	7.1E-14	63299	
rs12390237	X	3,612,081	*PRKX*	a	g	0.62	-0.046	1.7E-11	NA	NA	0	1.7E-11	36501	
rs1265091	6	31,108,129	*PSORS1C1*	t	c	0.20	0.057	3.2E-11	0.060	3.3E-05	16772	5.0E-15	64423	rs1063646 (0.89)
rs4445669	11	115,045,237	*CADM1*	t	c	0.46	-0.040	5.8E-11	-0.034	1.8E-02	9011	3.6E-12	63299	
rs6724073	2	218,236,786	*DIRC3*	t	c	0.74	0.051	1.3E-10	0.019	2.6E-01	9011	3.1E-10	61058	
rs56009477	8	23,356,964	*SLC25A37*	a	g	0.84	0.052	3.7E-10	0.034	7.7E-02	9011	1.1E-10	63299	
rs4933466	10	89,849,519	*PTEN*	a	g	0.60	0.040	5.1E-10	0.024	1.0E-01	9011	2.2E-10	63299	
rs13100823	3	185,514,088	*IGF2BP2*	t	c	0.31	-0.041	6.8E-10	-0.050	1.3E-03	9011	4.1E-12	63299	
rs11255790	10	8,682,180	*GATA3*	t	c	0.30	-0.041	6.8E-10	-0.026	9.7E-02	9011	2.5E-10	63299	
rs9298749	9	16,214,340	*C9orf92*	a	c	0.59	-0.039	8.8E-10	-0.029	5.4E-02	9011	1.6E-10	63299	
rs10957494	8	70,365,025	*SULF1*	a	g	0.69	-0.040	1.1E-09	-0.012	4.4E-01	9011	3.6E-09	63299	
rs77819282	17	44,762,589	*NSF*	a	g	0.24	0.045	1.1E-09	0.030	1.1E-01	7904	4.3E-10	62192	
rs1045476	16	4,015,313	*ADCY9*	a	g	0.18	0.049	2.4E-09	0.042	3.3E-03	17879	3.2E-11	72167	rs2531992 (0.82)
rs1157994	17	59,338,574	*BCAS3*	a	g	0.05	-0.090	5.3E-09	-0.047	1.6E-01	9011	4.0E-09	59243	
rs28133*	19	8.327,502	*CERS4*	t	c	0.08	0.072	1.1E-08	0.014	6.0E-01	9011	6.0E-08	58713	
rs55706568*	17	43,292,035	*FMNL1*	c	g	0.55	-0.038	1.1E-08	0.002	9.2E-01	9011	2.5E-07	63299	
rs28502438	3	149,220,109	*TM4SF4*	t	c	0.57	0.034	3.7E-08	0.041	5.2E-03	9011	7.3E-10	63299	
rs17398234*	8	66,934,902	*DNAJC5B*	t	g	0.97	-0.100	4.3E-08	-0.027	5.0E-01	9011	1.4E-07	63299	
FT4														
rs17185536	6	100,620,931	*LOC728012*	t	c	0.24	0.073	1.9E-19	0.053	3.5E-02	4532	2.7E-20	53801	
rs11626434	14	101,998,443	*DIO3OS*	c	g	0.36	0.058	4.1E-17	0.020	2.6E-01	5826	1.7E-16	55095	
rs225014	14	80,669,580	*DIO2*	t	c	0.64	0.054	1.8E-15	0.033	1.5E-03	14702	4.6E-17	63971	
rs11078333	17	16,049,626	*NCOR1*	a	t	0.53	0.051	9.9E-13	0.021	4.4E-02	13408	2.0E-12	62677	rs3815038 (0.96)
rs9356988	6	25,777,481	*SLC17A4*	a	g	0.27	-0.051	3.6E-12	-0.056	4.2E-03	5827	5.7E-14	55096	
rs10818937	9	127,015,440	*NEK6*	t	c	0.31	-0.048	1.3E-11	-0.018	9.3E-02	14702	4.9E-11	63971	
rs10946313	6	19,381,386	*ID4*	t	c	0.63	0.046	2.3E-11	0.032	7.9E-02	5827	6.2E-12	55096	
rs67583169	8	61,212,179	*CA8*	c	g	0.87	0.061	1.0E-10	0.064	2.3E-02	4532	7.1E-12	53801	
rs6785807	3	181,718,601	*SOX2-OT*	a	g	0.15	-0.059	2.5E-10	-0.041	8.5E-02	5827	6.9E-11	55096	
rs10119187	9	4,223,660	*GLIS3*	t	c	0.81	0.050	4.1E-09	0.037	6.0E-02	7667	8.0E-10	56936	
rs4954192	2	135,632,981	*ACMSD*	t	c	0.43	-0.041	8.4E-09	-0.018	6.0E-02	17778	9.3E-09	62680	rs6430552 (0.96)
rs56069042	18	57,914,644	*MC4R*	a	g	0.96	0.106	1.2E-08	0.067	8.0E-02	8928	3.7E-09	58197	
rs4149056	12	21,331,549	*SLCO1B1*	t	c	0.84	-0.051	1.3E-08	-0.042	1.0E-03	17822	6.3E-11	67091	
rs8063103	16	12,703,395	*SNX29*	c	g	0.85	-0.052	1.6E-08	-0.038	2.0E-01	4532	7.8E-09	53801	
rs73405691*	6	34,468,767	*PACSIN1*	a	g	0.87	0.056	1.6E-08	-0.002	9.2E-01	7673	2.1E-07	56942	
rs72783371*	10	13,436,195	*BEND7*	a	c	0.92	0.067	2.8E-08	-0.017	5.7E-01	7596	7.9E-07	56865	
rs11039355	11	47,737,501	*FNBP4*	t	c	0.34	-0.039	3.5E-08	-0.038	5.7E-04	13408	7.9E-11	62677	rs1064608 (0.95)
rs12907106	15	63,873,658	*USP3*	c	g	0.27	-0.041	3.7E-08	-0.020	4.0E-01	4532	3.7E-08	53801	
rs951366*	1	205,685,352	*NUCKS1*	t	c	0.62	0.037	4.4E-08	0.015	2.9E-01	8950	6.1E-08	58219	

(*) did not replicate because not all criteria specified in the methods were fulfilled. SE: standard erros; A1: effect allele; AF1: allele frequency of A1.
The proxy SNP and its R2 are provided in case a proxy SNP was used (applies for the Health2006 and Inter99 studies only).

Supplementary References

1. Rietzschel, E.-R. *et al.* Rationale, design, methods and baseline characteristics of the Asklepios Study. *Eur. J. Cardiovasc. Prev. Rehabil.* **14,** 179–91 (2007).
2. Ikram, M. A. *et al.* The Rotterdam Study: 2018 update on objectives, design and main results. *Eur. J. Epidemiol.* **32,** 807–850 (2017).
3. Kreiner-Møller, E., Medina-Gomez, C., Uitterlinden, A. G., Rivadeneira, F. & Estrada, K. Improving accuracy of rare variant imputation with a two-step imputation approach. *Eur. J. Hum. Genet.* **23,** 395–400 (2015).
4. Estrada, K. *et al.* GRIMP: a web- and grid-based tool for high-speed analysis of large-scale genome-wide association using imputed data. *Bioinformatics* **25,** 2750–2 (2009).
5. Pruim, R. J. *et al.* LocusZoom: regional visualization of genome-wide association scan results. *Bioinformatics* **26,** 2336–7 (2010).
