# Supplementary material for: Genome-wide analyses identify a role for SLC17A4 and AADAT in thyroid hormone regulation
Source: Nat Commun. 2018 Oct 26;9:4455. doi: 10.1038/s41467-018-06356-1 (PMC6203810; doi:10.1038/s41467-018-06356-1)
Supplement: Supplementary file 3 — Description of Additional Supplementary Files [file 41467_2018_6356_MOESM3_ESM.pdf]

Description of Additional Supplementary Files

File Name: Supplementary Data 1
Description: Characteristics of the study populations.

File Name: Supplementary Data 2
Description: Genes associated in mice with abnormal thyroid physiology or morphology and which are significant in the TSH and FT4 GWAS after multiple testing correction.

File Name: Supplementary Data 3
Description: eQTL lookup results.

File Name: Supplementary Data 4
Description: Co-localization results for all gene-tissue-phenotype combinations assessed.

File Name: Supplementary Data 5
Description: Pleiotropic effects of the genome-wide significant index SNPs or their proxies.

File Name: Supplementary Data 6
Description: : Description of new loci.
